# Chromosome-level assembly of the mustache toad genome using third-generation DNA sequencing and Hi-C analysis

**DOI:** 10.1093/gigascience/giz114

**Published:** 2019-09-23

**Authors:** Yongxin Li, Yandong Ren, Dongru Zhang, Hui Jiang, Zhongkai Wang, Xueyan Li, Dingqi Rao

**Affiliations:** 1 State Key Laboratory of Genetic Resources and Evolution, Kunming Institute of Zoology, Chinese Academy of Sciences, Kunming 650223, China; 2 Center for Ecological and Environmental Sciences, Northwestern Polytechnical University, Xi'an 710072, China; 3 National Engineering Laboratory of Marine Germplasm Resources Exploration and Utilization, Zhejiang Ocean University, Zhoushan 316022, China

**Keywords:** mustache toad, genome assembly, evolution, PacBio, Hi-C

## Abstract

**Background:**

The mustache toad, *Vibrissaphora ailaonica*, is endemic to China and belongs to the Megophryidae family. Like other mustache toad species, *V. ailaonica* males temporarily develop keratinized nuptial spines on their upper jaw during each breeding season, which fall off at the end of the breeding season. This feature is likely result of the reversal of sexual dimorphism in body size, with males being larger than females. A high-quality reference genome for the mustache toad would be invaluable to investigate the genetic mechanism underlying these repeatedly developing keratinized spines.

**Findings:**

To construct the mustache toad genome, we generated 225 Gb of short reads and 277 Gb of long reads using Illumina and Pacific Biosciences (PacBio) sequencing technologies, respectively. Sequencing data were assembled into a 3.53-Gb genome assembly, with a contig N50 length of 821 kb. We also used high-throughput chromosome conformation capture (Hi-C) technology to identify contacts between contigs, then assembled contigs into scaffolds and assembled a genome with 13 chromosomes and a scaffold N50 length of 412.42 Mb. Based on the 26,227 protein-coding genes annotated in the genome, we analyzed phylogenetic relationships between the mustache toad and other chordate species. The mustache toad has a relatively higher evolutionary rate and separated from a common ancestor of the marine toad, bullfrog, and Tibetan frog 206.1 million years ago. Furthermore, we identified 201 expanded gene families in the mustache toad, which were mainly enriched in immune pathway, keratin filament, and metabolic processes.

**Conclusions:**

Using Illumina, PacBio, and Hi-C technologies, we constructed the first high-quality chromosome-level mustache toad genome. This work not only offers a valuable reference genome for functional studies of mustache toad traits but also provides important chromosomal information for wider genome comparisons.

## Data Description

**Figure 1:**
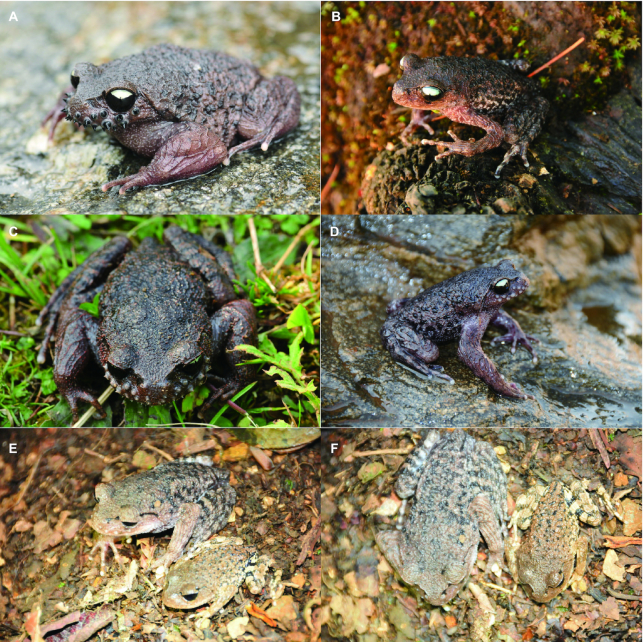
The mustache toad, *Vibrissaphora ailaonica*. (**A**) The adult male individual with spines in the upper jaw. (**B**) The adult female individual. (**C**) The adult male individual during the process of spines shedding from the upper jaw. (**D**) The adult male individual without spines (after spine have been shed) in the upper jaw. (**E**) The body size of the mustache toad, side view: male (left) and female (right). (**F**) The body size of mustache toad, top view: male (left) and female (right).

The mustache toad, *Vibrissaphora ailaonica* (NCBI:txid428466), is an amphibian belonging to the Megophryidae family that is endemic to China (including the China–Vietnam border) [1–3]. This mustache toad species exhibits many interesting features, including unique keratinized spines along the upper jaw [1, 4–6]. These spines grow repeatedly in sexually mature males during the breeding season, and fall off at the end of this process [5–8] (Fig. 1). This morphological difference between males and females is further highlighted by their sexual dimorphism in body size (males are significantly larger than females). The spines (and body size) may be used as a weapon for sexually mature individuals to compete for nests and mating opportunities [7, 9, 10]. Another unique aspect of the mustache toad is that breeding occurs during the cold season, whereas most frogs and toads breed in the warmer months [1]. However, despite the importance of the mustache toad in terms of dynamic spine development and sexual dimorphism in body size, few genomic resources exist for this species. In fact, to date, no next-generation sequencing data have been reported in the *Vibrissaphora* genus. The lack of genome sequence and transcriptome data for *V. ailaonica* has hindered identification of functional genes related to their attractive and dynamic appearance (e.g., spine and body size). The shortage of amphibian genomes represented in the Genome 10K/Vertebrate Genomes Projects makes it necessary to analyze other important genomes to study phylogenetic relationships in amphibians on a larger scale [11].

In the present study, we combined genomic sequencing data from Illumina short reads, Pacific Biosciences (PacBio) long reads, and Hi-C data to generate the first chromosome-level reference genome for the mustache toad. The completeness and continuity of the genome were comparable with those of other important amphibian species. The high-quality reference genome generated in this study will facilitate research on population genetic traits and functional gene identification related to important characteristics of the mustache toad.

## Analyses and Methods

### Sampling and sequencing

During the breeding season (in February), a male mustache toad (*V. ailaonica*) with keratinized nuptial spines on its upper jaw was caught for sequencing from Ailao Mountain (Fig. 1). To obtain sufficient high-quality DNA for the PacBio Sequel platform (Pacific Biosciences, USA), the mustache toad was dissected, and fresh liver tissue was used for DNA extraction using phenol/chloroform extraction. DNA quality was checked by agarose gel electrophoresis, and high-integrity DNA molecules were obtained. Other tissues, including spines, brain, stomach, intestine, liver, lung, spleen, blood, and tongue, were snap-frozen in liquid nitrogen for 10 min. These 9 organs/tissues were stored at −80°C for RNA-sequencing (RNA-seq) analysis. Isolated total RNA was used to isolate intact poly (A) + RNA using the NEBnext Ultra-Directional RNA Library Prep kit (NEB, protocol B) for library construction. The messenger RNA (mRNA) was further fragmented and randomly primed during first-strand synthesis by reverse transcription. This procedure was followed by second-strand synthesis with DNA polymerase I to create double-stranded complementary DNA fragments using Transcriptor First Strand cDNA Synthesis Kit (Roche).

For the Hi-C experiments, collected blood was used for library construction. The blood sample (150 µL) was cross-linked for 10 min with formaldehyde (1% final concentration), after which glycine (0.2 M final concentration) was added for 5 min to stop the cross-linking process. The sample was then stored until required for further analysis.

Extracted DNA was sequenced using the Illumina and PacBio Sequel platforms. Short reads generated from the Illumina platform were used to estimate genome size and to correct errors in the assembled genome, and the PacBio long reads were used for genome assembly. To this end, 5 libraries with insertion lengths of 220 or 500 bp were sequenced on an Illumina HiSeq 2500 platform, generating 150-bp paired-end reads. A 20-kb library was constructed using the PacBio platform, according to the manufacturer's protocols. Finally, we obtained 225.03 Gb of Illumina short reads and 277.15 Gb of PacBio long reads (Table 1, Additional Tables S1 and S2). The mean N50 length of subreads was 14.78 kb, providing ultra-long genomic sequences for the following assembly and analysis (Additional Table S2).

**Table 1:** Sequencing data used for mustache toad genome assembly and annotation

Sequencing type	Platform	Library size (bp)	Clean data (Gb)	Application
Genome long reads	PacBio Sequel	20,000	277.15	Contig assembly
Genome short reads	Illumina HiSeq 2500	250	225.03	Genome survey, genome base correction, and genome assessment
Genome Hi-C reads	Illumina HiSeq X-Ten	250	378.78	Chromosome construction
Transcriptome short reads	Illumina HiSeq 4000	250	14.18	Genome annotation and assessment

RNA-seq samples were obtained by mixing an equal amount of RNA extracted from each tissue that had been stored and used for library construction. After sequencing on the Illumina HiSeq 4000 platform, we obtained 14.18 Gb of sequencing data (Table 1, Additional Table S3). Four Hi-C libraries were constructed using the same sample with same parameters, and sequenced on the Illumina Hiseq X-ten platform, which generated 378.78 Gb of clean data (Table 1, Additional Table S4).

### Genome characteristic estimation

Illumina short reads were filtered for quality as follows. First, adaptors were removed from the sequencing reads. Then, read pairs were excluded if any 1 read had >10% “N,” and read pairs with >50% low-quality bases were removed. Finally, PCR duplicates produced during library construction were removed.

**Figure 2:**
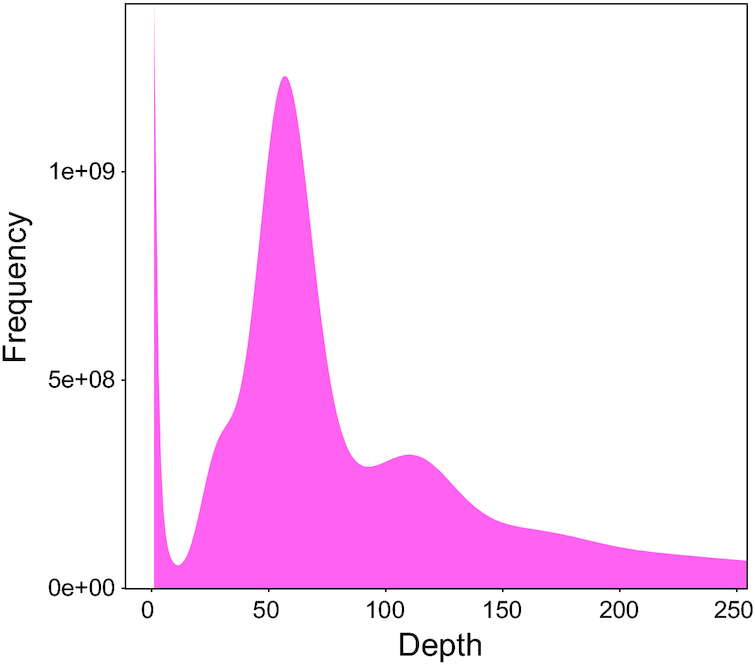
17-mer analysis of *Vibrissaphora ailaonica* genome characteristics.

Filtered reads were used to estimate genome size and other characteristics. Using the *k*-mer method, we calculated the 17-mer depth frequency distribution in the mustache toad. Genome size was estimated as follows:

$$\begin{equation*}G = {\rm{TK}}{{\rm{N}}_{{\rm{17 - mer}}}}{\rm{/ PKF}}{{\rm{D}}_{{\rm{17 - mer}}}},\end{equation*}$$

where TKN_17-mer_ is the total *k*-mer number and PKFD_17-mer_ is the peak *k*-mer frequency depth of 17-mer. We estimated a genome size of 3.52 Gb (peak = 54) and found heterozygous, repeated sequence peaks, suggesting that the mustache toad genome exhibits complex genome assembly (Fig. 2).

### Genome assembly using PacBio long reads and Hi-C data

Based on 38 single-molecule real-time cells, and using the PacBio Sequel platform, we generated 277.15 Gb of subreads (Table 1, Additional Table S2). The mean and N50 length of subreads was 9.65 and 14.78 kb, respectively (Additional Table S2). All long reads were assembled using wtdbg software [12] (WTDBG, RRID: SCR_01 7225). As a result, we obtained a 3.95-Gb genome assembly, with a contig N50 length of 739.54 kb. However, although the size of the genome assembly was comparable to the estimated *k*-mer result, the end result was a slightly larger. This may be associated with the complexity of the mustache toad genome (which has a high rate of heterozygosity and repetitive sequences). Redundancy in the genome assembly was removed using Redundans software (v0.13c) [13], with an identity of 0.7 and overlap of 0.7. This resulted in a genome assembly of 3.58 Gb and a contig N50 length of 834.90 kb. To ensure that all contigs removed were not real sequences, we used BUSCO [14] and the mapping ratio of Illumina reads in both the raw genome and the redundancy-filtered genome. Results of these checks indicated that the parameters used in the redundancy-filtered step were appropriate for this study (Additional Table S5). To further improve the quality and accuracy of our genome assembly, Illumina short reads were used to polish the genome using Pilon software (Pilon, RRID: SCR_01 4731, v1.21) [15] at the single-base level.

**Figure 3:**
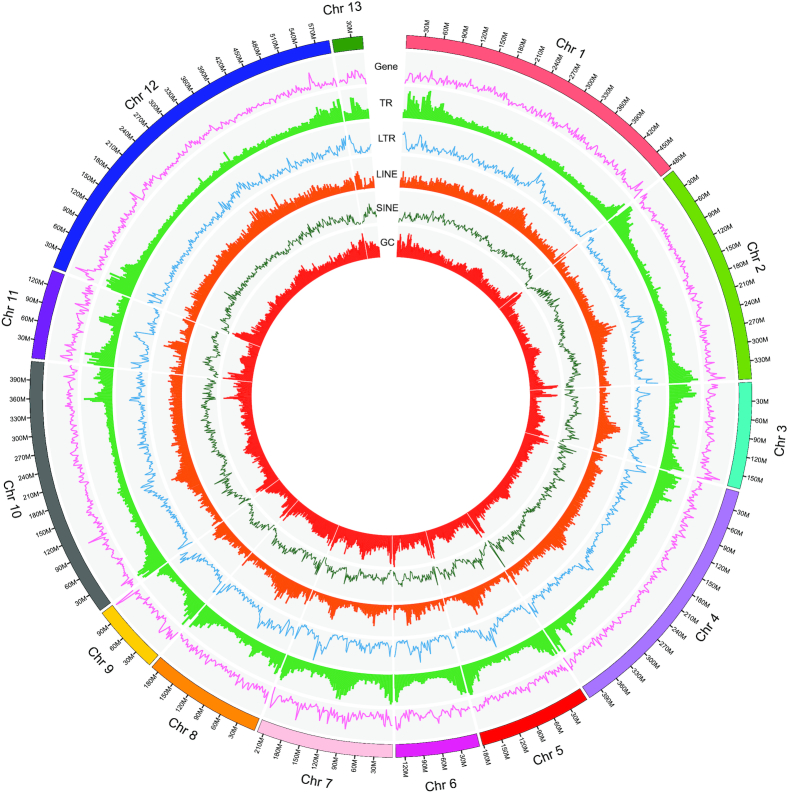
Circos graph showing characteristics of the mustache toad genome. From outer circle to inner ring: gene distribution, tandem repeats (TR), long tandem repeats (LTR), long interspersed nuclear elements (LINE), short interspersed nuclear elements (SINE), and guanine-cytosine (GC) content.

Hi-C data were used to improve the connection integrity of the contigs (15,899 contigs). We obtained 378.78 Gb of Hi-C sequencing data, which was first filtered using Hic-Pro (v2.10.0) [16] (Table 1, Additional Table S4) and then mapped to the polished mustache toad genome [17]. The locations and directions of the contigs were determined by 3D *de novo* assembly (3d-DNA) software (v180419) [18], with default parameters. Most contigs were then successfully clustered and anchored in 13 groups (Fig. 3) [19]. Finally, we obtained the first chromosome-level, high-quality mustache toad assembly (3.53 Gb) with a scaffold N50 length of 412.42 Mb, which provides a solid genomic resource to assist further study of the mustache toad (Table 2).

**Table 2:** Assembly data for the mustache toad genome

Term	Wtdbg contig		Hi-C scaffold	
Size (bp)	No.	Size (bp)	No.
N90	153,029	4,866	134,864,763	11
N80	301,658	3,285	181,461,513	8
N70	456,829	2,334	220,042,448	6
N60	624,716	1,671	359,321,214	5
N50	821,125	1,180	412,424,790	4
Maximum length (bp)	9,978,207		592,710,058	
Total size (bp)	3,530,531,046		3,535,795,546	
Total No. (>100 bp)	15,899		5,370	

Note: These data pertain to genome assembly. Wtdbg contig was the genome assembled by wtdbg and 2-round pilon error correction. Hi-C scaffold was the genome finished by Hi-C assembly.

### Genome assembly evaluation

The quality of a genome assembly is directly related to the accuracy and completeness of protein-coding gene prediction. Therefore, we evaluated the assembled mustache toad genome using 3 methods. First, the assembled genome was compared against the core gene set in BUSCO (BUSCO, RRID:SCR_015008, v2.0) [14]. We found 245 (80.8%) and 833 (85.1%) conserved core genes in the mustache toad genome using the eukaryote and metazoan databases, respectively (Table 3). When we further considered the fragmented BUSCO genes found in the genome, there were 272 (89.7%) and 881 (90.1%) conserved core genes in the eukaryote and metazoan databases, respectively. These results indicated that the assembled mustache toad genome is comparable with published amphibian genomes (Table 3).

**Table 3:** Assessment of genome assembly and annotation completeness of the mustache toad and other amphibian genomes, using BUSCO

Library	*V. ailaonica* (eukaryota)	*V. ailaonica* (metazoa)	*Nanorana parkeri* (eukaryota)	*Xenopus tropicalis* (eukaryota)	*Rhinella marina* (eukaryota)	*Rana catesbeiana* (eukaryota)	*Ambystoma mexicanum* (eukaryota)
Complete genes (%)	80.8	85.1	90.1	90.1	90.4	58.0	24.4
Complete and single-copy genes (%)	78.2	83.6	87.8	88.1	86.1	55.4	23.4
Complete and duplicated genes (%)	2.6	1.5	2.3	2.0	4.3	2.6	1.0
Fragmented genes (%)	8.9	4.9	3.6	2.0	3.3	20.8	24.4
Missing genes (%)	10.3	10.0	6.3	7.9	6.3	21.2	51.2

Note: Both “eukaryote” and “metazoan” are 2 core gene sets in the BUSCO database.

Second, all filtered short reads generated from the Illumina platform were aligned to the genome using BWA software (BWA, RRID:SCR_010910, v0.7.12) [20]; 1,778 million clean reads could be mapped to the genome, accounting for 97.78% of total clean reads (Additional Table S6).

Third, RNA-seq reads were *de novo* assembled using Bridger software (Bridger, RRID:SCR_017039, version: r2014–12-01) [21], with redundant transcripts removed by TGICL [22]. This resulted in 19,876 transcripts (Additional Table S7). These transcripts were then aligned to the genome, with 17,878 transcripts (89.95%) found in the assembled genome, and 94.52% of transcripts being longer than 1 kb (Additional Table S8). Analysis of N50 length and BUSCO results revealed that the mustache toad genome was comparable to that of other published amphibian genomes (Tables 2–4), indicating that our assembled mustache toad genome exhibited high completeness and accuracy.

**Table 4:** Quality data for several published amphibian genomes

Species	Contig N50 (bp)	Scaffold N50 (bp)	Genome size (bp)	Genome BUSCO (eukaryota) (%)
*Nanorana parkeri*	32,798	1,069,101	2,053,867,363	90.1
*Xenopus tropicalis*	71,041	135,134,832	1,440,398,454	90.1
*Rhinella marina*	166,489	167,498	2,551,759,918	90.4
*Rana catesbeiana*	5,415	39,363	6,250,353,185	58.0
*Ambystoma mexicanum*	216,366	3,052,786	32,393,605,577	24.4

The GC distribution of the mustache toad genome, and that of other vertebrate species, was calculated using the slide window method. GC distributions were similar, with a mean GC content of 43.68% in the mustache toad, and 36.60–44.49% in other species (Additional Figure S1).

### Genome annotation

Tandem Repeats Finder (TRF, v4.04) [23] was used to identify repetitive elements, and RepeatModeler software (RepeatModeler, RRID:SCR_015027, v1.0.4) was used to detect transposable elements (TEs) in the mustache toad genome. Then, the *de novo* library of repeats produced by RepeatModeler analysis and the repbase (RepBase16.02) database were used for RepeatMasker (RepeatMasker, RRID:SCR_012954, version: open-4.0) [24] analysis to identify homologous repeats. RepeatProteinMask was used to query the TE protein database at the protein level. Last, we identified 2.45 Gb of repeat sequences, accounting for 69.48% of the estimated genome size (Additional Table S9). Among these repeat sequences, 60.87% (2.15 Gb) was predicted by the *de novo* method (Table 5).

**Table 5:** D*e novo*–annotated repeat sequences in the mustache toad genome

Type	Length (bp)	Percentage in genome (%)
DNA	350,793,270	9.94
LINE	297,954,803	8.45
SINE	11,009,363	0.31
LTR	307,317,539	8.71
Other	43,867,330	1.24
Satellite	9,696,790	0.27
Simple repeat	125,397,072	3.55
Unknown	1,114,326,962	31.59
Total	2,147,505,764	60.87

**Figure 4:**
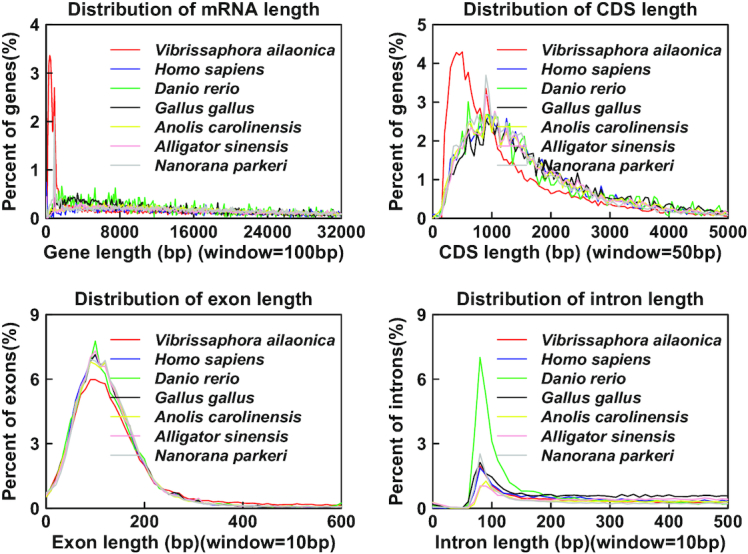
Length distributions of annotated protein-coding genes in *Vibrissaphora ailaonica, Homo sapiens, Danio rerio, Gallus gallus, Anolis carolinensis, Alligator sinensis*, and *Nanorana parkeri*.

After repeat sequence annotation, we masked all repeats, except for the tandem repeat sequences, for protein-coding gene annotation. Augustus software (Augustus, RRID:SCR_008417, v2.5.5) [25] was used to *de novo–*predict coding genes using a zebrafish (*Danio rerio*) dataset as the training species. For the homology-based method, protein sequences of chordate species, including *D. rerio* (GCF_0 00002035.6) [26], *Nanorana parkeri* (GCF_000 935625.1) [27], *Homo sapiens* (GCF_0 00001405.38) [28], *Gallus gallus* (GCF_0 00002315.5) [29], *Pelodiscus sinensis* (GCF_000 230535.1) [30], *Xenopus laevis* (GCF_0 016 63975.1) [31], and *Petromyzon marinus* [32], were downloaded and aligned against the mustache toad genome using the TBLASTN module (TBLASTN, RRID:SCR_011822, BLAST version: 2.3.0). The transcripts assembled by RNA-seq reads were first translated into amino acids and then aligned to the genome using TBLASTN software for gene annotation. EVidenceModeler (EVidenceModeler, RRID:SCR_014659, version: r2012–06-25) [33] was used to integrate results from the 3 methods, and genes with poor transcriptome evidence support were filtered out. Finally, 26,227 high-quality protein-coding genes were predicted in the mustache toad genome. The distributions of mRNA, coding sequences, and exon and intron lengths were comparable to those of closely related species (Fig. 4).

Gene functional annotation can help to elucidate gene function. Thus, we aligned all 26,227 protein-coding genes to protein databases, including InterProScan, KEGG, SwissProt, and TrEMBL. Results showed that most of the genes obtained could be annotated from these functional databases (Table 6).

**Table 6:** Functional annotation for protein-coding genes in the mustache toad genome

Database	Annotated gene No. (%)
Interpro	12,997 (49.56)
KEGG	10,035 (38.26)
SwissProt	12,410 (47.32)
Trembl	17,916 (68.31)

### Phylogenetic tree and divergence time analysis

**Figure 5:**
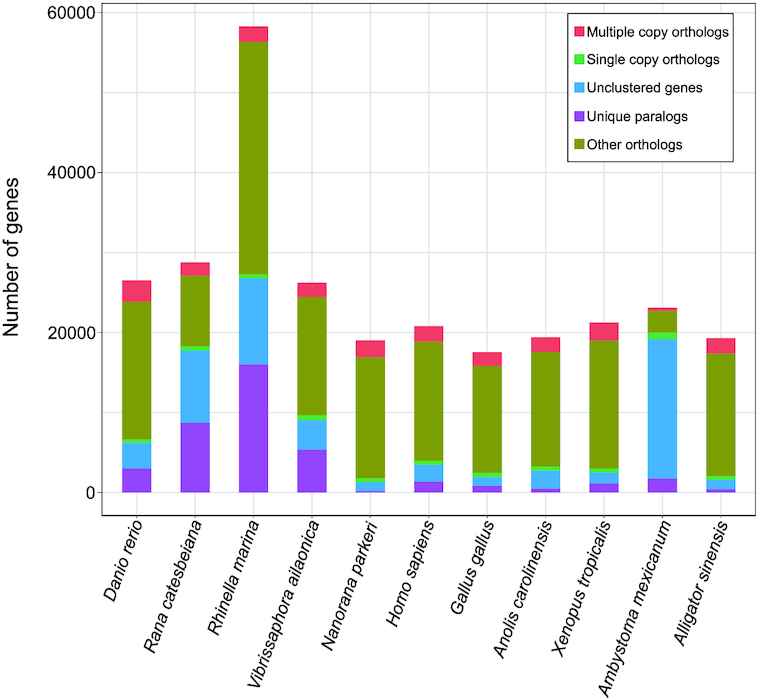
The statistics of gene family among 11 species including *Danio rerio, Rana catesbeiana, Rhinella marina, Vibrissaphora ailaonica, Nanorana parkeri, Homo sapiens, Gallus gallus, Anolis carolinensis, Xenopus tropicalis, Ambystoma mexicanum*, and *Alligator sinensis*.

To reveal phylogenetic relationships between the mustache toad and other closely related species, we identified the single-copy genes among these species. First, protein sequences, including those of *D. rerio* (GCF_0 00002035.6) [26], *N. parkeri* (GCF_000 935625.1) [27], *H. sapiens* (GCF_0 00001405.38) [28], *G. gallus* (GCF_0 00002315.5) [29], *Anolis carolinensis* (GCF_00 0090745.1) [34], *Xenopus tropicalis* (GCF_0 00004195.3) [31], *Rhinella marina* (GigaDB) [35], *Rana catesbeiana* (GCA_0 022 84835.2) [36], *Ambystoma mexicanum* [37, 38], and *Alligator sinensis* (GCF_000 455745.1) [39], were downloaded from the NCBI. The longest transcript of each gene in each species was selected. BLASTP (BLASTP, RRID:SCR_001010, BLAST version: 2.2.24) was then used to align these protein sequences from the 11 species (including the mustache toad), with an e-value of 1e−5. Homology relationships (including orthologs and paralogs) were then determined using OrthoMCL software (v1.4) [40]. Genes with only 1 copy in the species were identified as single-copy genes. In total, 238 genes were identified (Fig. 5). Detailed statistics about gene families are shown in Additional Table S10.

**Figure 6:**
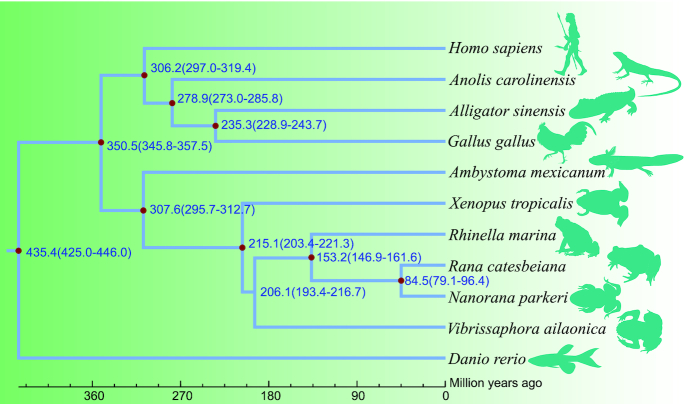
The phylogenetic relationships among these species. The species including *Danio rerio, Rana catesbeiana, Rhinella marina, Vibrissaphora ailaonica, Nanorana parkeri, Homo sapiens, Gallus gallus, Anolis carolinensis, Xenopus tropicalis, Ambystoma mexicanum*, and *Alligator sinensis*. Blue numbers represent divergence time with 95% confidence interval. The red dot represents the fossil record used in the node.

The 238 single-copy genes were aligned using MUSCLE software (MUSCLE, RRID:SCR_011812, v3.8.31) [41, 42] and concatenated to supergenes for maximum-likelihood–based phylogenetic analyses. We performed phylogenetic analysis, with zebrafish as the outgroup, using RAxML software (RAxML, RRID:SCR_006086, v8.2.3) [43], with the parameter “-m” for PROTGAMMAAUTO. Results indicated that the mustache toad has a close relationship with the ancestor of the marine toad (*R. marina*), bullfrog (*R. catesbeiana*), and Tibetan frog (*N. parkeri*), with topological relationships in other clades found to be the same as reported previously (Fig. 6). To further investigate the divergence time of these species, especially toads and frogs, the MCMCTREE model (part of the PAML software package; PAML, RRID:SCR_014932, v4.8) [44] was used with 3 datasets (4-fold degenerate sites [4dTVs], first-codon sites, and second-codon sites) extracted from the single-copy genes as the input file. Fossil records were downloaded from the TIMETREE website [45] and used to calibrate the results. Results from the 3 different datasets were very similar, showing that the mustache toad diverged from the common ancestor of the marine toad, bullfrog, and Tibetan frog ∼206.1 million years ago (Fig. 6; Additional Figs S2 and S3).

### Gene family expansion and contraction

We performed gene family expansion and contraction analysis using CAFÉ software (CAFÉ, RRID:SCR_005983, v4.0) [46] and found 201 and 326 expanded and contracted gene families in the mustache toad (*P* < 0.05), respectively. Using the Gene Ontology (GO) and KEGG databases, functional enrichment analysis of expanded gene families revealed 210 GO terms (adjusted *P* < 0.05) and 9 KEGG pathways (*q* < 0.05) to be significantly enriched (Additional Tables S11 and S12). The expanded gene families were mainly related to metabolic processes, intermediate filament terms, enzyme activities, and immune terms. For example, cellular metabolic process (adjusted *P* = 6.06E−14), intermediate filament (adjusted *P* = 3.42E−15), keratin filament (adjusted *P* = 2.94E−13), endoribonuclease activity (adjusted *P* = 9.19E−08), and immune response (*q* = 8.36E−03) were enriched (Additional Tables S11 and S12). In addition, for the contracted gene families, 220 GO terms (adjusted *P* < 0.05) and 9 KEGG pathways (*q* < 0.05) were enriched, respectively (Additional Tables S13 and S14). These enriched terms were mainly involved in ion binding and transporter activity, including neurotransmitter transporter activity (adjusted *P* = 1.89E−11), sodium ion transmembrane transporter activity (adjusted *P* = 3.33E−06), and secondary active transmembrane transporter activity (adjusted *P* = 1.86E−08) (Additional Tables S13 and S14). Thus, these biological processes may be related to the special characteristics of the mustache toad.

### Relative evolutionary rate of species

The evolutionary rate of species can reflect its evolutionary history and status. The relative evolutionary rate of the mustache toad to other closely related species was analyzed using LINTRE [47] and MEGA (MEGA, RRID:SCR_000667, v7.0.26) software. Two-cluster analysis was applied to test the molecular evolution of multiple sequences in a phylogenetic context, based on concatenated supergenes (protein sequences) using "tpcv" (a module in LINTRE software). Concatenated supergenes were also used for Tajima's relative rate test. We used zebrafish as the outgroup in both methods and found that, except for the axolotl, the mustache toad had a relatively faster evolutionary rate than its closely related species (e.g., *X. tropicalis, R. marina, R. catesbeiana*, and *N. parkeri*) (Additional Tables S15 and S16). The crocodile had a slower evolutionary rate, relative to its closely related species, which is consistent with previous work [48] (Additional Tables S15 and S16).

## Discussion

Using Illumina, PacBio, and Hi-C sequencing technologies, we report the first chromosome-level genome assembly of the mustache toad. We successfully annotated the high-quality protein-coding genes by integrating results from 3 different methods. Phylogenetic analysis indicated that the mustache toad is closely related to the marine toad, bullfrog, and Tibetan frog. Analysis showed that the mustache toad had a faster evolutionary rate, relative to most other closely related species studied. Analysis of the expansion and contraction of gene families identified several biological processes and pathways, such as metabolism and intermediate filaments, suggesting that these terms may relate to the special adaptations of the mustache toad to its habitat. This work not only offers a valuable chromosome-level genomic data for comparative genomics analysis but also provides important genomic data for studying the mustache toad traits.

## Supplementary Material

giz114_GIGA-D-19-00099_Original_Submission

giz114_GIGA-D-19-00099_Revision_1

giz114_GIGA-D-19-00099_Revision_2

giz114_Response_to_Reviewer_Comments_Original_Submission

giz114_Response_to_Reviewer_Comments_Revision_1

giz114_Reviewer_1_Report_Original_Submission Michael Hiller -- 5/4/2019 Reviewed

giz114_Reviewer_1_Report_Revision_1 Michael Hiller -- 6/19/2019 Reviewed

giz114_Reviewer_2_Report_Original_Submission Taejoon Kwon -- 5/6/2019 Reviewed

giz114_Supplementary_Files

## Availability of supporting data and materials

Raw sequencing data were deposited in the NCBI database under accession number PRJNA523649. Genome assembly and annotation results are available via the *GigaScience* repository, GigaDB [49].

## Additional files

**Additional Figure S1:** The GC content in these genomes. The species include *Gallus gallus, Vibrissaphora ailaonica, Alligator sinensis, Nanorana parkeri, Homo sapiens, Anolis carolinensis, Xenopus tropicalis*, and *Danio rerio*.

**Additional Figure S2:** The divergence time of these species (using first-codon sites). The species include *Danio rerio, Rana catesbeiana, Rhinella marina, Vibrissaphora ailaonica, Nanorana parkeri, Homo sapiens, Gallus gallus, Anolis carolinensis, Xenopus tropicalis, Ambystoma mexicanum*, and *Alligator sinensis*.

**Additional Figure S3:** The divergence time of these species (using second-codon sites). The species include *Danio rerio, Rana catesbeiana, Rhinella marina, Vibrissaphora ailaonica, Nanorana parkeri, Homo sapiens, Gallus gallus, Anolis carolinensis, Xenopus tropicalis, Ambystoma mexicanum*, and *Alligator sinensis*.

**Additional Table S1:** Illumina sequencing clean data.

**Additional Table S2:** PacBio Sequel sequencing data.

**Additional Table S3:** RNA-sequencing clean data.

**Additional Table S4:** Hi-C sequencing clean data.

**Additional Table S5:** Comparison of the BUSCO and Illumina read mapping results between the raw genome and redundancy-filtered genome.

**Additional Table S6:** Illumina read mapping ratio to the assembled genome.

**Additional Table S7:** The statistics of assembled transcripts by Bridger software. The redundant transcripts were removed by TGICL software.

**Additional Table S8:** Transcript mapping ratio to the assembled genome.

**Additional Table S9:** Annotated repeat sequences in our assembled genome.

**Additional Table S10:** Gene families among these species. The species include *Danio rerio, Rana catesbeiana, Rhinella marina, Vibrissaphora ailaonica, Nanorana parkeri, Homo sapiens, Gallus gallus, Anolis carolinensis, Xenopus tropicalis, Ambystoma mexicanum*, and *Alligator sinensis*.

**Additional Table S11:** Gene Ontology (GO) enrichment analysis of expanded gene families.

**Additional Table S12:** KEGG enrichment analysis of expanded gene families.

**Additional Table S13:** Gene Ontology (GO) enrichment analysis of contracted gene families.

**Additional Table S14:** KEGG enrichment analysis of contracted gene families.

**Additional Table S15:** Two-cluster analysis of mustache toad and other species.

**Additional Table S16:** The relative evolutionary rate of mustache toad and other species analyzed by Tajima's test.

## Abbreviations

BLAST: Basic Local Alignment Search Tool; bp: base pairs; BUSCO: Benchmarking Universal Single-Copy Orthologs; BWA: Burrows–Wheeler Aligner; Gb: gigabase pairs; GC: guanine-cytosine; GO: Gene Ontology; Hi-C: high-throughput chromosome conformation capture; kb: kilobase pairs; KEGG: Kyoto Encyclopedia of Genes and Genomes; LINE: long interspersed nuclear element; LTR: long tandem repeat; Mb: megabase pairs; mRNA: messenger RNA; NCBI: National Center for Biotechnology Information; PAML: Phylogenetic Analysis by Maximum Likelihood; RAxML: Randomized Axelerated Maximum Likelihood; RNA-seq: RNA-sequencing; SINE: short interspersed nuclear element; TR: tandem repeat; TrEMBL: Translation of European Molecular Biology Laboratory.
