# Supplementary material for: Chromosome-level assembly of the mustache toad genome using third-generation DNA sequencing and Hi-C analysis
Source: Gigascience. 2019 Sep 23;8(9):giz114. doi: 10.1093/gigascience/giz114 (PMC6755253; doi:10.1093/gigascience/giz114)
Supplement: giz114_GIGA-D-19-00099_Original_Submission [file giz114_giga-d-19-00099_original_submission.pdf]

GigaScience

Chromosomal-level assembly of the mustache toad genome using third-generation DNA sequencing and Hi-C analysis

--Manuscript Draft--

**Manuscript Number:**	GIGA-D-19-00099
**Full Title:**	Chromosomal-level assembly of the mustache toad genome using third-generation DNA sequencing and Hi-C analysis
**Article Type:**	Data Note
**Funding Information:**	National Key Research and Development Program of China (2017YFC0505202) — Dr. Dingqi Rao<br>National Natural Science Foundation of China (30270175) — Dr. Dingqi Rao<br>National Natural Science Foundation of China (30870278) — Dr. Dingqi Rao<br>National Natural Science Foundation of China (31372165) — Dr. Dingqi Rao

Abstract:

Background

The mustache toad, Vibrissaphora ailaonica, is an endemic species to China belonging to the Megophryidae family. Like other mustache toad species, V. ailaonica males develop temporary keratinized nuptial spines on their upper jaw during each breeding season and fall off when the breeding season ends, which probably lead to the reverse of the sexual size dimorphism, namely the size of the male get larger than female. To investigate the genetic mechanism of the repeatedly develop the keratinized spines, a high-quality reference genome of mustache toad would be a valuable resource.

Findings

For genome construction, we generated 225 Gb of short reads and 277 Gb of long reads using Illumina and Pacific Biosciences (PacBio) sequencing, respectively. The sequencing data were assembled into a 3.53 Gb genome assembly with a contig N50 length of 821 Kb. Additionally, we applied Hi-C technology to identify contacts among contigs, then assembled contigs into scaffolds and identified a genome assembly with 13 chromosomes and a scaffold N50 length of 412.42 Mb. Based on the 26,227 protein-coding genes annotated in the genome, we analyzed the phylogenetic relationships of the mustache toad with other chordate species. Results showed that the mustache toad has a relatively higher evolutionary rate and separated from the marine toad, bull frog, and Tibetan frog ancestor 194.8 million years ago. Furthermore, we identified 349 expanded gene families in the mustache toad, which were mainly enriched in immune pathway, keratin filament, and metabolic processes.

Conclusions

Using Illumina, PacBio, and Hi-C technologies, we constructed the first high-quality chromosomal-level mustache toad genome. This work not only offers a valuable reference genome for functional studies of mustache toad traits, but also provides important chromosome information for wider genome comparisons.

**Corresponding Author:**	Dingqi Rao, Ph.D<br>Kunming Institute of Zoology Chinese Academy of Sciences<br>Kunming, Yunnan CHINA
**Corresponding Author Secondary Information:**	
**Corresponding Author's Institution:**	Kunming Institute of Zoology Chinese Academy of Sciences

Powered by Editorial Manager® and ProduXion Manager® from Aries Systems Corporation

Corresponding Author's Secondary Institution:	
First Author:	Yongxin Li
First Author Secondary Information:	
Order of Authors:	Yongxin Li
	Yandong Ren
	Dongru Zhang
	Hui Jiang
	Zhongkai Wang
	Xueyan Li
	Dingqi Rao, Ph.D
Order of Authors Secondary Information:	
Additional Information:	
Question	Response
Are you submitting this manuscript to a special series or article collection?	No
**Experimental design and statistics**<br><br>Full details of the experimental design and statistical methods used should be given in the Methods section, as detailed in our Minimum Standards Reporting Checklist. Information essential to interpreting the data presented should be made available in the figure legends.<br><br>Have you included all the information requested in your manuscript?	Yes
**Resources**<br><br>A description of all resources used, including antibodies, cell lines, animals and software tools, with enough information to allow them to be uniquely identified, should be included in the Methods section. Authors are strongly encouraged to cite Research Resource Identifiers (RRIDs) for antibodies, model organisms and tools, where possible.<br><br>Have you included the information requested as detailed in our Minimum	Yes

Standards Reporting Checklist?	
**Availability of data and materials**<br><br>All datasets and code on which the conclusions of the paper rely must be either included in your submission or deposited in publicly available repositories (where available and ethically appropriate), referencing such data using a unique identifier in the references and in the “Availability of Data and Materials” section of your manuscript.<br><br>Have you have met the above requirement as detailed in our Minimum Standards Reporting Checklist?	Yes

Manuscript
Click here to access/download;Manuscript;manuscript.doc
Click here to view linked References

Chromosomal-level assembly of the mustache toad genome using third-generation DNA sequencing and Hi-C analysis

Yongxin Li[1,2,†], Yandong Ren[2,†], Dongru Zhang[1,†], Hui Jiang[3], Zhongkai Wang[2], Xueyan Li[1], Dingqi Rao[1,*]

1. State Key Laboratory of Genetic Resources and Evolution, Kunming Institute of Zoology, Chinese Academy of Sciences, Kunming 650223, China

2. Center for Ecological and Environmental Sciences, Northwestern Polytechnical University, Xi'an 710072, China

3. National Engineering Laboratory of Marine Germplasm Resources Exploration and Utilization, Zhejiang Ocean University, Zhoushan 316022, China

[†]These authors have the equal contribution.

[*]Corresponding author: Dingqi Rao (kizar@mail.kiz.ac.cn).

Abstract

Background: The mustache toad, *Vibrissaphora ailaonica*, is an endemic species to China belonging to the Megophryidae family. Like other mustache toad species, *V. ailaonica* males develop temporary keratinized nuptial spines on their upper jaw during each breeding season and fall off when the breeding season ends, which probably lead to the reverse of the sexual size dimorphism, namely the size of the male get larger than female. To investigate the genetic mechanism of the repeatedly develop the keratinized spines, a high-quality reference genome of mustache toad would be a valuable resource. **Findings:** For genome construction, we generated 225 Gb of short reads and 277 Gb of long reads using Illumina and Pacific Biosciences (PacBio) sequencing, respectively. The sequencing data were assembled into a 3.53 Gb genome assembly with a contig N50 length of 821 Kb. Additionally, we applied Hi-C technology to identify contacts among contigs, then assembled contigs into scaffolds and identified a genome assembly with 13 chromosomes and a scaffold N50 length of 412.42 Mb. Based on the 26,227 protein-coding genes annotated in the genome, we analyzed the phylogenetic relationships of the mustache toad with other chordate species. Results showed that the mustache toad has a relatively higher evolutionary rate and separated from the marine toad, bull frog, and Tibetan frog ancestor 194.8 million years ago. Furthermore, we identified 349 expanded gene families in the mustache toad, which were mainly enriched in immune pathway, keratin filament, and metabolic processes. **Conclusions:** Using Illumina, PacBio, and Hi-C technologies, we constructed the first high-quality chromosomal-level mustache toad genome. This work not only offers a valuable reference genome for functional studies of mustache toad traits, but also provides important chromosome information for wider genome

comparisons.

Keywords: Mustache toad; Genome assembly; Evolution; PacBio; Hi-C

Introduction

The mustache toad, *Vibrissaphora ailaonica* (NCBI Taxonomy ID: 428466), belongs to the Megophryidae family and is an endemic amphibian species to China (including the China-Vietnam border) [1-3]. This mustache toad species exhibits many interesting features, including unique keratinized spines along the upper jaw [1, 4-6]. These spines repeatedly grow in sexually mature males during the breeding season and fall off at the end of this process [5-8]. This morphological difference between males and females is further highlighted by their sexual dimorphism in body size (males are significantly larger than females) and may be used as a weapon for sexually mature individuals to compete for nests and mating opportunities [7, 9, 10]. Another unique aspect of the mustache toad is that breeding occurs during the cold season, unlike most frogs and toads which breed in the warmer months [1]. However, despite the importance of the mustache toad in spine dynamic development and sexual dimorphism in body size, the genomic resources for the species remain limited. To date, no next-generation sequencing (NGS) data have been reported in the *Vibrissaphora* genus. Therefore, this lack of genome sequence and transcriptome data for this important species (*V. ailaonica*) has hindered identification of genome-based functional genes related to their attractive dynamic body appearance (e.g., spine and body size). Besides, as there is such a shortage of amphibian genomes in Genome 10K project, and it is necessary to

analyze other important genomes to large-scale study the phylogenetic relationships in amphibian [11].

In this study, we combined genomic sequencing data from Illumina short reads, PacBio long reads, and Hi-C data to generate the first chromosomal-level reference genome for the mustache toad. The completeness and continuity of the genome were comparable with that of other important amphibian species. The high-quality reference genome generated in this study will facilitate research on population genetic traits and functional gene identification related to important characteristics of the mustache toad, which will, in turn, accelerate the development of more efficient body size control techniques and improve the artificial breeding industry for other economically important species.

Sampling and sequencing

A male mustache toad (*V. ailaonica*) with keratinized nuptial spines on its upper jaw was caught from the Ailao Mountain during the breeding season for sequencing (Figure 1). To obtain sufficient high-quality DNA for the PacBio Sequel platform (Pacific Biosciences, USA), the mustache toad was dissected, and fresh liver tissue was used for DNA extraction using phenol/chloroform extraction. DNA quality was checked by agarose gel electrophoresis, with excellent integrity DNA molecules were obtained. Other tissues, including spines, brain, stomach, intestine, liver, lung, spleen, blood, and tongue, were snap-frozen in liquid nitrogen for 10 min and then stored at −80℃ for subsequent use. In the Hi-C experiments, the collected blood was used for library construction. The blood sample (150 ul) was cross-linked for 10 min with formaldehyde (1% final concentration), after which glycine (0.2 M final

concentration) was added for 5 min to stop the cross-linking process, with the sample then stored until the further analysis.

Extracted DNA was sequenced using the Illumina and PacBio Sequel platforms. The short reads generated from the Illumina platform were used for estimation of genome size and error correction of the assembled genome, and the PacBio long reads were used for genome assembly. To this end, five libraries with insertion lengths of 220 bp or 500 bp were generated from the Illumina HiSeq 2500 platform and a 20 Kb library was constructed using the PacBio platform according to the manufacturers' protocols. Finally, we obtained 225.03 Gb of Illumina short reads and 277.15 Gb of PacBio long reads (Table 1; Additional File: Tables S1 and S2). The average subreads N50 length reached to 14.78 Kb, providing ultra-long genomic sequences for the following assembly and analysis (Additional File: Table S2). The RNA-seq samples were obtained by mixing an equal amount of RNA extracted from each tissue stored and used for library construction. After sequencing on the Illumina HiSeq 4000 platform, we obtained 14.18 Gb of sequencing data (Table 1; Additional File: Table S3). Four Hi-C libraries were constructed and sequenced on the Illumina Hiseq X-ten platform, which generated 378.78 Gb of clean data (Table 1; Additional File: Table S4).

Genome characteristics estimation

The Illumina short reads were quality filtered by the following steps: First, the adaptors were removed from the sequencing reads. Second, read pairs were excluded if any one read had more than 10% "N". Third, read pairs with low quality base more than 50% were removed. Fourth, the PCR duplicates produced during library construction in read pairs were removed.

The filtered reads were used for estimation of genome size and other characteristics. Using the k-mer method, we calculated the 17-mer depth frequency distribution in the mustache toad. Genome size was estimated by: $G = TKN_{17\text{-mer}} / PKFD_{17\text{-mer}}$, where $TKN_{17\text{-mer}}$ is the total kmer number and $PKFD_{17\text{-mer}}$ is the peak kmer frequency depth of 17-mer. We estimated a genome size of 3.52 Gb (peak = 54) and found a heterozygous and repeat sequences peak, suggesting that the mustache toad genome exhibits complex genome assembly (Figure 2).

Genome assembly by PacBio long reads and Hi-C data

Based on 38 single-molecular real-time cells by the PacBio Sequel platform, we generated 277.15 Gb of subreads (Table 1; Additional File: Table S2). The average and N50 length of subreads were 9.65 Kb and 14.78 Kb, respectively (Additional File: Table S2). All the long reads were used for genome assembly using wtdbg software (https://github.com/ruanjue/wtdbg-1.2.8). As a result, we obtained a 3.95 Gb genome assembly with a contig N50 length of 739.54 Kb. However, although the size of the genome assembly was comparable with the estimation k-mer result, it was a slightly larger. This may be due to the complexity of the mustache toad genome (high heterozygous rate and repeat sequences). Then the redundant sequences in the genome assembly were removed using Redundans software (v0.13c) [12] with an identity of 0.7 and overlap of 0.7, resulting in a genome assembly of 3.58 Gb and contig N50 length of 834.90 Kb. To further improve the quality and accuracy of our genome assembly, the Illumina short reads were used to polish the genome using Pilon software (RRID:SCR_014731, v1.21) [13] at the single-base level. The Hi-C data were used to improve the connection integrity of the contigs (15,899 contigs). We

obtained 378.78 Gb of Hi-C sequencing data, which were first filtered by Hic-Pro (v2.10.0) [14] (Table 1; Additional File: Table S4), and then mapped to the polished mustache toad genome [15]. The location and direction of the contigs were determined by 3D *de novo* assembly (3d-DNA) software (v180419) [16] with default parameters. Most contigs were then successfully clustered and anchored on 13 groups (Figure 3) [17]. Finally, we obtained the first chromosomal-level high quality mustache toad assembly (3.53 Gb) with a scaffold N50 length of 412.42 Mb, providing a solid genomic resource for further study of the mustache toad (Table 2).

Genome assembly evaluation

Genome assembly quality is directly related to the accuracy and completeness of protein-coding gene prediction. Therefore, we evaluated the assembled mustache toad genome using three methods. First, the assembled genome was compared with the core gene set in BUSCO software (RRID:SCR_015008, v2.0) [18]. We found 245 (80.8%) and 833 (85.1%) conserved core genes in the mustache toad genome using the eukaryote and metazoan databases, respectively (Table 3). When we further considered the fragmented BUSCO genes found in the genome, there were 272 (89.7%) and 881 (90.1%) conserved core genes found in the eukaryote and metazoan databases, respectively (Table 3). Second, we aligned all filtered short reads generated from the Illumina platform to the genome using BWA software (RRID:SCR_010910, v0.7.12) [19] and found 1,778 million clean reads that could be mapped to the genome, accounting for 97.78% of total clean reads (Additional File: Table S5). Third, the RNA-seq reads were *de novo* assembled using Bridger software

(RRID:SCR_017039, version: r2014-12-01) [20], with redundant transcripts removed by TGICL [21], resulting in 19,876 transcripts (Additional File: Table S6). These transcripts were then aligned to the genome, with 17,878 transcripts (89.95%) found in the assembled genome and 94.52% of transcripts longer than 1 Kb (Additional File: Table S7). Besides, we analyzed the N50 length and BUSCO results and found that the mustache toad genome was comparable with other published amphibian genomes (Table 2 and Table 3; Additional File: Table S8). The GC distribution of the mustache toad and closely related species was calculated using the slide window method. Results showed that their GC distributions were similar to each other, with an average GC content of 43.68% in the mustache toad and 36.60% to 44.49% in other species (Additional File: Figure S1). These results indicate that our assembled mustache toad genome exhibited high completeness and accuracy.

Genome annotation

We used Tandem Repeat Finder (TRF, v4.04) [22] to identify repetitive elements and RepeatModeler software (RRID:SCR_015027, v1.0.4) to detect transposable elements (TEs) in the mustache toad genome. Then, the *de novo* library of repeats produced by RepeatModeler analysis and the repbase (RepBase16.02) database were then used for RepeatMasker (RRID:SCR_012954, version: open-4.0) [23] analysis to identify homologous repeats. RepeatProteinMask was used to query the TE protein database at the protein level. Lastly, we identified 2.45 Gb of repeat sequences, which accounts for 69.48% of the estimated genome size (Additional File: Table S9). Among these repeat sequences, 60.87% (2.15 Gb) was predicted by the *de novo* method (Table 4).

After repeat sequence annotation, we masked all repeats, except for the tandem repeat sequences, for protein-coding gene annotation. Augustus software (RRID:SCR_008417, v2.5.5) [24] was used to *de novo* predict coding genes using a zebrafish (*Danio rerio*) dataset as the train species. For the homology-based method, protein sequences of closely related species, including *D. rerio* (GCF_000002035.6) [25], *Nanorana parkeri* (GCF_000935625.1) [26], *Homo sapiens* (GCF_000001405.38) [27], *Gallus gallus* (GCF_000002315.5) [28], *Pelodiscus sinensis* (GCF_000230535.1) [29], *Xenopus laevis* (GCF_001663975.1) [30], and *Petromyzon marinus* [31] (https://genomes.stowers.org/organism/Petromyzon/marinus), were downloaded and aligned against the mustache toad genome using the TBLASTN module (BLAST version: 2.3.0). The transcripts assembled by RNA-seq reads were first translated into amino acids and then aligned to the genome using TBLASTN software for gene annotation. EVidenceModeler (RRID:SCR_014659, version: r2012-06-25) [32] was used to integrate results from the three methods, and genes with poor transcriptome evidence support were filtered out. Finally, 26,227 high-quality protein-coding genes were predicted in the mustache toad genome. Moreover, the distributions of mRNA, CDS, exon, and intron lengths were comparable with closely related species (Figure 4).

Gene functional annotation can help to elucidate gene function. Thus, we aligned all 26,227 protein-coding genes to protein databases, including InterProScan, KEGG, SwissProt, and TrEMBL. Results showed that most obtained genes could be annotated in these functional databases (Table 5).

Phylogenetic tree and divergence time analysis

To reveal the phylogenetic relationships of the mustache toad with other closely related species, we identified the single-copy genes among the species. First, protein sequences, including those of *D. rerio* (GCF_000002035.6) [25], *N. parkeri* (GCF_000935625.1) [26], *H. sapiens* (GCF_000001405.38) [27], *G. gallus* (GCF_000002315.5) [28], *Anolis carolinensis* (GCF_000090745.1) [33], *Xenopus tropicalis* (GCF_000004195.3) [30], *Rhinella marina* (GigaDB) [34], *Rana catesbeiana* (GCA_002284835.2) [35], *Ambystoma mexicanum* (www.axolotl-omics.org) [36], and *Alligator sinensis* (GCF_000455745.1) [37], were downloaded from NCBI and the longest transcript of each gene in each species was selected. The BLASTP program (BLAST version: 2.2.24) was then used to align these protein sequences among these 11 species (including the mustache toad) with an e-value of 1e-5. The homolog relationships (including ortholog and paralog) were then determined using OrthoMCL software (v1.4) [38]. Genes with only one copy in the species were identified as single-copy genes. In total, 238 genes were identified (Figure 5), with the detailed results of gene family statistics shown in the supplementary information (Additional File: Table S10). The 238 single-copy genes were aligned using MUSCLE software (RRID:SCR_011812, v3.8.31) [39, 40] and concatenated to supergenes for maximum-likelihood-based phylogenetic analyses. We performed phylogenetic analysis, with zebrafish as the outgroup, using RAxML software (RRID:SCR_006086, v8.2.3) [41] with the parameter -m for PROTGAMMAAUTO. Results indicated that the mustache toad exhibited a close relationship with the ancestor of the marine toad (*R. marina*), bull frog (*R. catesbeiana*), and Tibetan frog (*N. parkeri*), with topological relationships in other clades found to be the same as reported previously (Figure 6). To further investigate the divergence time of these species, especially toad and frogs, the

MCMCTREE model in PAML software (RRID:SCR_014932, v4.8) [42] was used with the four-fold degenerate site (4dTV) extracted from the single-copy genes as the input file. Fossil records were downloaded from the TIMETREE website (www.timetree.org) and used to calibrate the results. We found that the mustache toad diverged with the common ancestor of the marine toad, bull frog, and Tibetan frog about 194.8 million years ago (Figure 6).

Gene family expansion and contraction

We performed gene family expansion and contraction analysis using CAFÉ software (RRID:SCR_005983, v4.0) [43], and found 349 and 2,607 expanded and contracted gene families in the mustache toad ($P < 0.05$), respectively. Using the GO/KEGG databases, functional enrichment analysis of the expanded gene families found 174 GO terms (adjusted P-value < 0.05) and 29 KEGG pathways (q-value < 0.05) to be significantly enriched (Additional File: Tables S11 and S12). The expanded gene families were mainly related to metabolic processes, intermediate filament terms, enzyme activities, and immune terms. For example, the cellular metabolic process (adjusted P-value = 2.37E-14), intermediate filament (adjusted P-value = 3.92E-13), keratin filament (adjusted P-value = 1.66E-12), endoribonuclease activity (adjusted P-value = 1.06E-07), and immune response (q-value = 4.81E-06) were enriched (Additional File: Tables S11 and S12). In addition, for the contracted gene families, 226 GO terms (adjusted P-value < 0.05) and 11 KEGG pathways (q-value < 0.05) were enriched, respectively (Additional File: Tables S13 and S14). These enriched terms were mainly involved in ion binding and transporter activity, including neurotransmitter transporter activity (adjusted P-value = 4.20E-09), sodium ion transmembrane transporter

activity (adjusted *P*-value = 1.55E-08), and secondary active transmembrane transporter activity (adjusted *P*-value = 6.37E-08) (Additional File: Tables S13 and S14). Thus, these biological processes may be related to the special characteristics of the mustache toad.

Relative evolutionary rate of species

The evolutionary rate of species can reflect its evolution history and status. The relative evolutionary rate of the mustache toad to other closely related species was analyzed using LINTRE [44] and MEGA (RRID:SCR_000667, v7.0.26) softwares. Two-cluster analysis was applied to test the molecular evolution of multiple sequences in a phylogenetic context based on the concatenated supergenes (protein sequences) using *tpcv* (a module in LINTRE software). The concatenated supergenes were also used for Tajima's relative rate test. We used zebrafish as the outgroup in both methods, and found that, except for the axolotl, the mustache toad had a relatively faster evolutionary rate than its closely related species (e.g., *X.tropicalis*, *R. marina*, *R. catesbeiana*, and *N. parkeri*) (Additional File: Tables S15 and S16). The crocodile had a slower evolutionary rate relative to its closely related species, and this result is consistent with previous study [45] (Additional File: Tables S15 and S16).

Conclusions

Using Illumina, PacBio, and Hi-C sequencing technologies, we reported on the first chromosomal-level genome assembly of the mustache toad. We successfully annotated 26,227 protein-coding genes by integrating the results of three different methods. The phylogenetic analysis results indicated that the mustache toad has a close relationship with the marine toad,

bull frog, and Tibetan frog, and diverged at 194.8 MYA with their common ancestor. Analysis showed that the mustache toad had a faster evolutionary rate relative to most other closely related species studied. Expansion and contraction of gene family analysis identified several biological processes and pathways, such as metabolism and intermediate filaments, suggesting that these terms may relate to the special adaptations of the mustache toad to its habitat.

Availability of supporting data

The raw sequencing data were deposited in the NCBI database under accession number PRJNA523649. The genome assembly and annotation results are available via the GigaScience repository GigaDB.

Additional files

Figure S1: The GC content in these genomes.

Table S1: The statistics of Illumina sequencing clean data.

Table S2: The statistics of PacBio Sequel sequencing data.

Table S3: The statistics of RNA-seq clean data.

Table S4: The statistics of Hi-C sequencing clean data.

Table S5: The statistics of Illumina reads mapping ratio to the assembled genome.

Table S6: The statistics of assembled transcripts by Bridger software.

Table S7: The statistics of transcripts mapping ratio to the assembled genome.

Table S8: The quality statistics of several published amphibian genomes.

Table S9: The statistics of the annotated repeat sequences in our assembled genome.

Table S10: The statistics of gene family among these species.

Table S11: The GO enrichment analysis of expanded gene families.

Table S12: The KEGG enrichment analysis of expanded gene families.

Table S13: The GO enrichment analysis of contracted gene families.

Table S14: The KEGG enrichment analysis of contracted gene families.

Table S15: Two cluster analysis of mustache toad and other species.

Table S16: The relative evolutionary rate of mustache toad and other species analyzed by Tajima's Test.

Abbreviations

BLAST: Basic Local Alignment Search Tool; BUSCO: Benchmarking Universal Single-Copy Orthologs; BWA: Burrows-Wheeler Aligner; CDS: Coding DNA Sequence; DNA: Deoxyribonucleic Acid; GO: Gene Ontology; Hi-C: High-throughput chromosome conformation capture; KEGG: Kyoto Encyclopedia of Genes and Genomes; MHC: Major Histocompatibility Complex; NCBI: National Center for Biotechnology Information; NR: Non-Redundant Protein Sequence Database; PCR: Polymerase Chain Reaction; RNA: Ribonucleic Acid; RNA-seq: RNA sequencing.

Conflicts of interest

The authors declare that they have no competing interests.

Funding

This work was supported by the National Key Research and Development Program of China (NO. 2017YFC0505202) and National Natural Science Foundation of China (NO. NSFC-30270175; NO. NSFC-30870278; NO. NSFC-31372165).

Author contributions

D.R. designed the project; D.R. and D.Z. collected the samples; Y.L. and Y.R. estimated the genome size and assembled the genome; Y.L. polished the assembled genome and employed the Hi-C analysis; H.J. performed the genome annotation; Y.L. and Z.W. assessed the quality of the genome assembly; Y.L. and Y.R. constructed the phylogenetic tree and determined divergence time, relative evolutionary rate of species, and expansion and contraction of gene families. Y.L., D.R., Y.R., and X.L. wrote the manuscript.

References

1. Liang F and Changyuan Y. Amphibians of China. Science Press: Beijing; 2016.
2. Matsui M, Hamidy A, Murphy RW, Khonsue W, Yambun P, Shimada T, et al. Phylogenetic relationships of megophryid frogs of the genus Leptobrachium (Amphibia, Anura) as revealed by mtDNA gene sequences. Molecular Phylogenetics & Evolution. 2010;56 1:259-72.
3. Matsui M. A New Leptobrachium (Vibrissaphora) from Laos (Anura: Megophryidae). Current Herpetology. 2013;32 2:182-9.
4. Liu C HS, Zhao EJAHS, Chengdu, Old Ser. Preliminary study of genus Vibrissaphora (Amphibia: Salientia) and discussion on problems of amphibian classification. Copeia. 1980;3:1-9.
5. Rao DQ and Wilkinson JA. Phylogenetic relationships of the mustache toads inferred from mtDNA sequences. Molecular Phylogenetics & Evolution. 2008;46 1:61-73.
6. Zheng Y, Li S and Fu J. A phylogenetic analysis of the frog genera Vibrissaphora and Leptobrachium, and the correlated evolution of nuptial spine and reversed sexual size dimorphism. Molecular Phylogenetics & Evolution. 2008;46 2:695-707.
7. Zheng Y, Deng D, Li S and Fu J. Aspects of the breeding biology of the Omei

mustache toad (*Leptobrachium boringii*): Polygamy and paternal care. Amphibia-Reptilia. 2010;31 2:183-94.

8. Zhang W, Guo Y, Li J, Huang L, Kazitsa EG and Wu H. Transcriptome analysis reveals the genetic basis underlying the seasonal development of keratinized nuptial spines in *Leptobrachium boringii*. Bmc Genomics. 2016;17 1:978.
9. Zheng Y, Rao D, Murphy RW and Zeng X. Reproductive Behavior and Underwater Calls in the Emei Mustache Toad, *Leptobrachium boringii*. Asian Herpetological Research. 2011;02 4:199-215.
10. Hudson CM, Xianjin HE and Jinzhong FU. Keratinized Nuptial Spines Are Used for Male Combat in the Emei Moustache Toad (*Leptobrachium boringii*). Asian Herpetological Research. 2011;02 3:142-8.
11. Genome 10K: a proposal to obtain whole-genome sequence for 10,000 vertebrate species. The Journal of heredity. 2009;100 6:659-74. doi:10.1093/jhered/esp086.
12. Pryszcz LP and Gabaldón T. Redundans: an assembly pipeline for highly heterozygous genomes. Nucleic Acids Research. 2016;44 12:e113-e.
13. Walker BJ, Abeel T, Shea T, Priest M, Abouelliel A, Sakthikumar S, et al. Pilon: an integrated tool for comprehensive microbial variant detection and genome assembly improvement. PLoS One. 2014;9 11:e112963. doi:10.1371/journal.pone.0112963.
14. Servant N, Varoquaux N, Lajoie BR, Viara E, Chen CJ, Vert JP, et al. HiC-Pro: an optimized and flexible pipeline for Hi-C data processing. Genome Biology. 2015;16 1:259.
15. Durand N, Shamim M, Machol I, Rao SP, Huntley M, Lander E, et al. Juicer Provides a One-Click System for Analyzing Loop-Resolution Hi-C Experiments. Cell Systems. 2016;3 1:95-8.
16. Dudchenko O, Batra SS, Omer AD, Nyquist SK, Hoeger M, Durand NC, et al. De novo assembly of the *Aedes aegypti* genome using Hi-C yields chromosome-length scaffolds. Science. 2017;356 6333:92.
17. Wilkinson JA. A New Species of the Genus Vibrissaphora (Anura: Megophryidae) from Yunnan Province, China. Herpetologica. 2006;62 1:90-5.
18. Simão FA, Waterhouse RM, Panagiotis I, Kriventseva EV and Zdobnov EM. BUSCO: assessing genome assembly and annotation completeness with single-copy orthologs. Bioinformatics. 2015;31 19:3210-2.
19. Li H and Durbin R. Fast and accurate short read alignment with Burrows-Wheeler transform. 2009.
20. Chang Z, Li G, Liu J, Zhang Y, Ashby C, Liu D, et al. Bridger: a new framework for de novo transcriptome assembly using RNA-seq data. Genome Biology. 2015;16 1:30.
21. Pertea G, Huang X, Liang F, Antonescu V, Sultana R, Karamycheva S, et al. TIGR Gene Indices clustering tools (TGICL): a software system for fast clustering of large EST datasets. Bioinformatics. 2003;19 5:651-2.
22. Benson G. Tandem repeats finder: a program to analyze DNA sequences. Nucleic Acids Res. 1999;27 2:573-80.
23. Bedell JA, Korf I, . and Gish W, . MaskerAid: a performance enhancement to RepeatMasker. Bioinformatics. 2000;16 11:1040-1.

24. Stanke M and Waack S. Gene prediction with a hidden Markov model and a new intron submodel. Bioinformatics. 2003;19 suppl_2:215--25.

25. Kerstin H, Clark MD, Torroja CF, James T, Camille B, Matthieu M, et al. The zebrafish reference genome sequence and its relationship to the human genome. Nature. 2013.

26. Yan-Bo S, Zi-Jun X, Xue-Yan X, Shi-Ping L, Wei-Wei Z, Xiao-Long T, et al. Whole-genome sequence of the Tibetan frog *Nanorana parkeri* and the comparative evolution of tetrapod genomes. Proc Natl Acad Sci U S A. 2015;11 112:E1257-E62.

27. Lander ES, Linton LM, Birren B, Nusbaum C, Zody MC, Baldwin J, et al. Initial sequencing and analysis of the human genome. Nature. 2001;409 6822:860-921. doi:10.1038/35057062.

28. Sequence and comparative analysis of the chicken genome provide unique perspectives on vertebrate evolution. Nature. 2004;432 7018:695-716. doi:10.1038/nature03154.

29. Wang Z, Pascual-Anaya J, Zadissa A, Li W, Niimura Y, Huang Z, et al. The draft genomes of soft-shell turtle and green sea turtle yield insights into the development and evolution of the turtle-specific body plan. Nat Genet. 2013;45 6:701-6. doi:10.1038/ng.2615.

30. Session AM, Uno Y, Kwon T, Chapman JA, Toyoda A, Takahashi S, et al. Genome evolution in the allotetraploid frog *Xenopus laevis*. Nature. 2016;538 7625:336-43. doi:10.1038/nature19840.

31. Smith JJ and Timoshevskaya N. The sea lamprey germline genome provides insights into programmed genome rearrangement and vertebrate evolution. 2018;50 2:270-7. doi:10.1038/s41588-017-0036-1.

32. Haas BJ, Salzberg SL, Zhu W, Pertea M, Allen JE, Orvis J, et al. Automated eukaryotic gene structure annotation using EVidenceModeler and the Program to Assemble Spliced Alignments. Genome Biology. 2008;9 1:R7.

33. Jessica AL, Federica DP, Manfred G, Christina W, Lesheng K, Evan M, et al. The genome of the green anole lizard and a comparative analysis with birds and mammals. Nature. 2011.

34. Nowoshilow S, Schloissnig S, Fei JF, Dahl A, Pang AWC, Pippel M, et al. The axolotl genome and the evolution of key tissue formation regulators. Nature. 2018;554 7690.

35. Hammond SA, Warren RL, Vandervalk BP, Kucuk E, Khan H, Gibb EA, et al. The North American bullfrog draft genome provides insight into hormonal regulation of long noncoding RNA. 2017;8 1:1433. doi:10.1038/s41467-017-01316-7.

36. Smith JJ, Timoshevskaya N, Timoshevskiy VA, Keinath MC, Hardy D and Voss SR. A chromosome-scale assembly of the axolotl genome. Genome Res. 2019;29 2:317-24. doi:10.1101/gr.241901.118.

37. Wan QH, Pan SK, Hu L, Zhu Y, Xu PW, Xia JQ, et al. Genome analysis and signature discovery for diving and sensory properties of the endangered Chinese alligator. Cell research. 2013;23 9:1091-105. doi:10.1038/cr.2013.104.

38. Li L, Jr SC and Roos DS. OrthoMCL: identification of ortholog groups for eukaryotic genomes. Genome Research. 2003;13 9:2178-89.

39. Edgar RC. MUSCLE: multiple sequence alignment with high accuracy and high throughput. Nucleic Acids Res. 2004;32 5:1792-7. doi:10.1093/nar/gkh340.
40. Edgar RC. MUSCLE: a multiple sequence alignment method with reduced time and space complexity. Bmc Bioinformatics. 2004.
41. Alexandros S. RAxML version 8: a tool for phylogenetic analysis and post-analysis of large phylogenies. Bioinformatics. 2014;30 9:1312-3.
42. Yang Z. PAML 4: phylogenetic analysis by maximum likelihood. Molecular biology and evolution. 2007;24 8:1586-91. doi:10.1093/molbev/msm088.
43. Tijl DB, Nello C, Demuth JP and Hahn MW. CAFE: a computational tool for the study of gene family evolution. Bioinformatics. 2006;22 10:1269-71.
44. Takezaki N, Rzhetsky A, and Nei M. Phylogenetic test of the molecular clock and linearized trees. Molecular Biology & Evolution. 1995;12 5:823-33.
45. Green RE, Braun EL, Joel A, Dent E, Ngan N, Glenn H, et al. Three crocodilian genomes reveal ancestral patterns of evolution among archosaurs. Science. 2014;346 6215:1254449.

Tables and Figures

Table 1: Sequencing data used for mustache toad genome assembly and annotation.

Sequencing type	Platform	Library size (bp)	Clean data (Gb)	Application
Genome long reads	PacBio Sequel	20,000	277.15	Contig assembly
Genome short reads	Illumina HiSeq 2500	250	225.03	Genome survey, genome base correction, and genome assessment
Genome Hi-C reads	Illumina HiSeq X-Ten	250	378.78	Chromosome construction
Transcriptome short reads	Illumina HiSeq 4000	250	14.18	Genome annotation and assessment

Table 2: Assembly statistics of the mustache toad genome.

	Wtdbg contig		Hi-C scaffold	
Term	Size (bp)	Number	Size (bp)	Number
N90	153,029	4,866	134,864,763	11
N80	301,658	3,285	181,461,513	8
N70	456,829	2,334	220,042,448	6
N60	624,716	1,671	359,321,214	5
N50	821,125	1,180	412,424,790	4
Max length (bp)	9,978,207		592,710,058	
Total size (bp)	3,530,531,046		3,535,795,546	
Total number (>100bp)	15,899		5,370	

Note: Statistics of genome assembly. Wtdbg contig was the genome assembled by wtdbg and 2-round pilon error-correction. Hi-C scaffold was the genome finished by Hi-C assembly.

Table 3: The BUSCO results of the mustache toad genome.

Library	eukaryota	metazoa
Complete BUSCOs (C)	245	833
Complete and single-copy BUSCOs (S)	237	818
Complete and duplicated BUSCOs (D)	8	15
Fragmented BUSCOs (F)	27	48
Missing BUSCOs (M)	31	97
Total BUSCO groups searched	303	978
Summarize	80.8%	85.1%

Table 4: The statistics of *de novo* annotated repeat sequences in mustache toad genome.

Type	Length (bp)	Percentage in genome (%)
DNA	350,793,270	9.943777
LINE	297,954,803	8.445989
SINE	11,009,363	0.312077
LTR	307,317,539	8.711390
Other	43,867,330	1.243487
Satellite	9,696,790	0.274870
Simple repeat	125,397,072	3.554574
Unknown	1,114,326,962	31.587320
Total	2,147,505,764	60.874369

Table 5: The functional annotation results of protein-coding genes in mustache toad.

Database	Annotated gene number	Percent (%)
Interpro	12,997	49.56
KEGG	10,035	38.26
SwissProt	12,410	47.32
Trembl	17,916	68.31

Figure 1: The mustache toad, *Vibrissaphora ailaonica*. (A) The adult male individual with spines in the upper jaw. (B) The adult female individual. (C) The adult male individual during the fall off process of spines in the upper jaw. (D) The adult male individual without spines (after fall off process of spines) in the upper jaw. (E) The body size of mustache toad in side view, male (left) and female (right). (F) The body size of mustache toad in top view, male (left) and female (right).

Figure 2: The 17-mer analysis of *Vibrissaphora ailaonica* genome characteristics.

Figure 3: The circos graph showing genome characteristics. From outer circle to inner ring are: gene distribution, tandem repeats (TR), long tandem repeats (LTR), long interspersed nuclear elements (LINE), short interspersed nuclear elements (SINE), and GC content.

Figure 4: The length distributions of annotated protein-coding genes in these species.

Figure 5: The statistics of gene family among these 11 species.

Figure 6: The phylogenetic relationships among these species. The blue numbers represent divergence time. The red dot represents the fossil record used in the node.

Figure 1

Click here to access/download;Figure;Figure 1.pdf

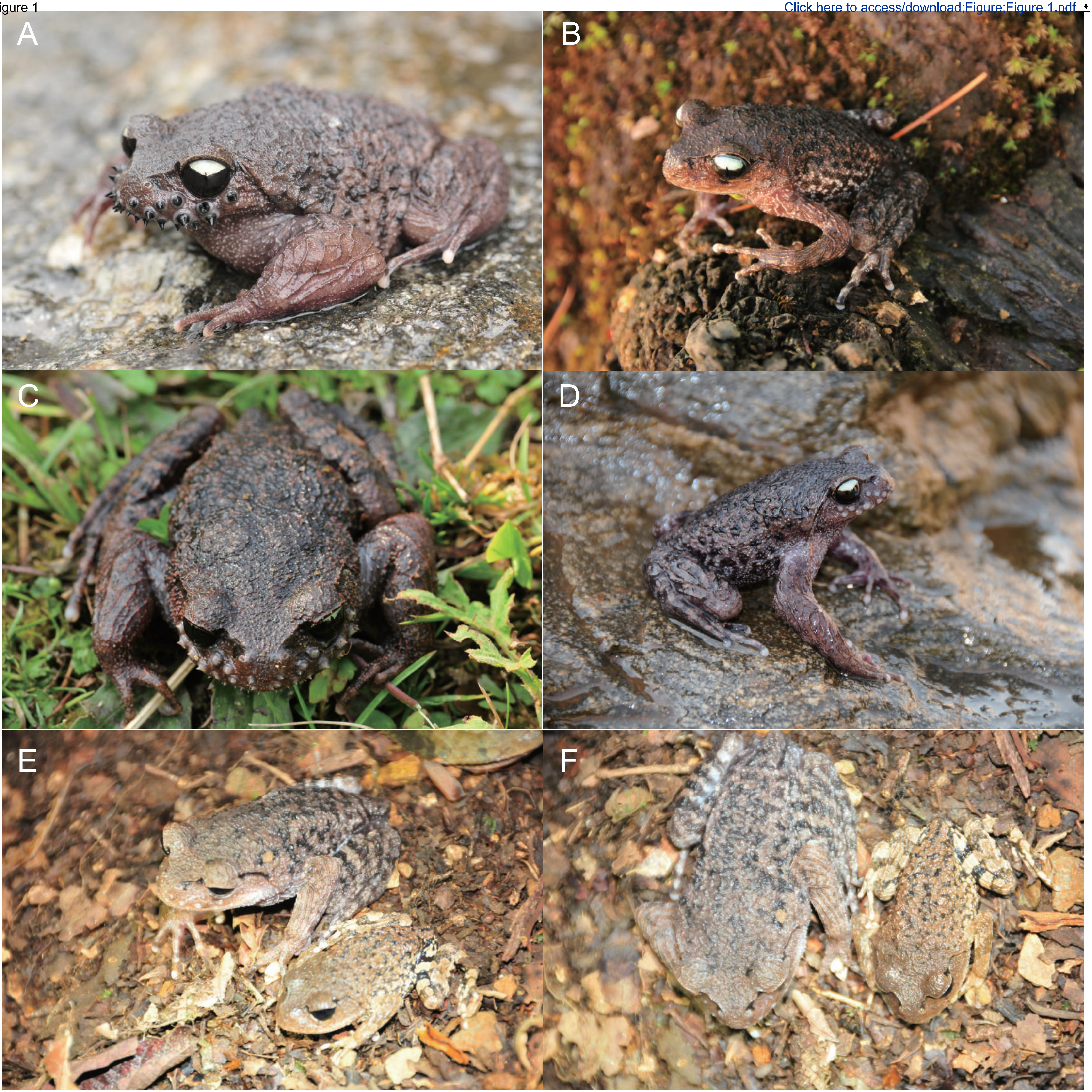

Figure 2

Click here to access/download;Figure;Figure 2.pdf

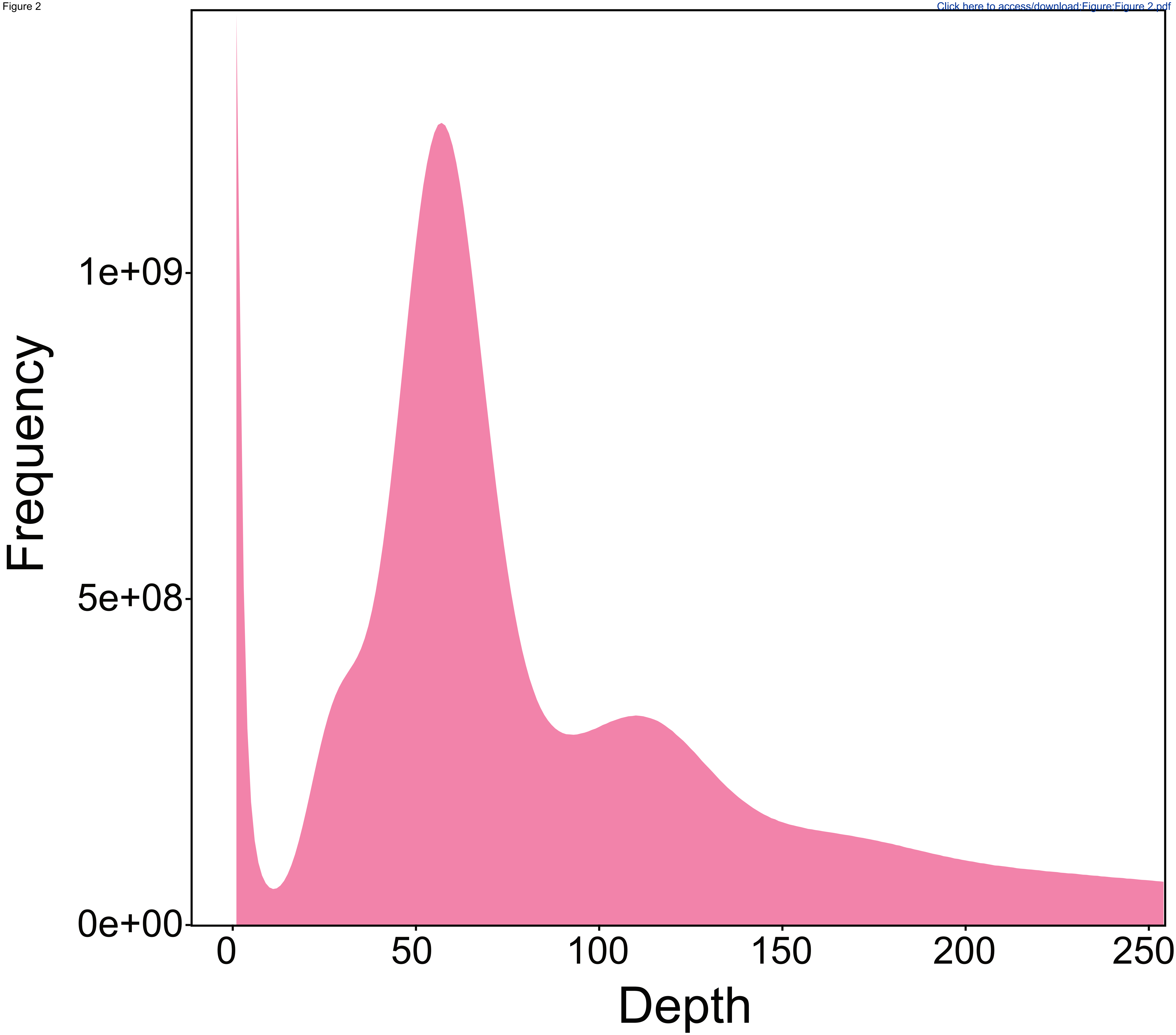

Figure 3

Click here to access/download;Figure;Figure 3.pdf

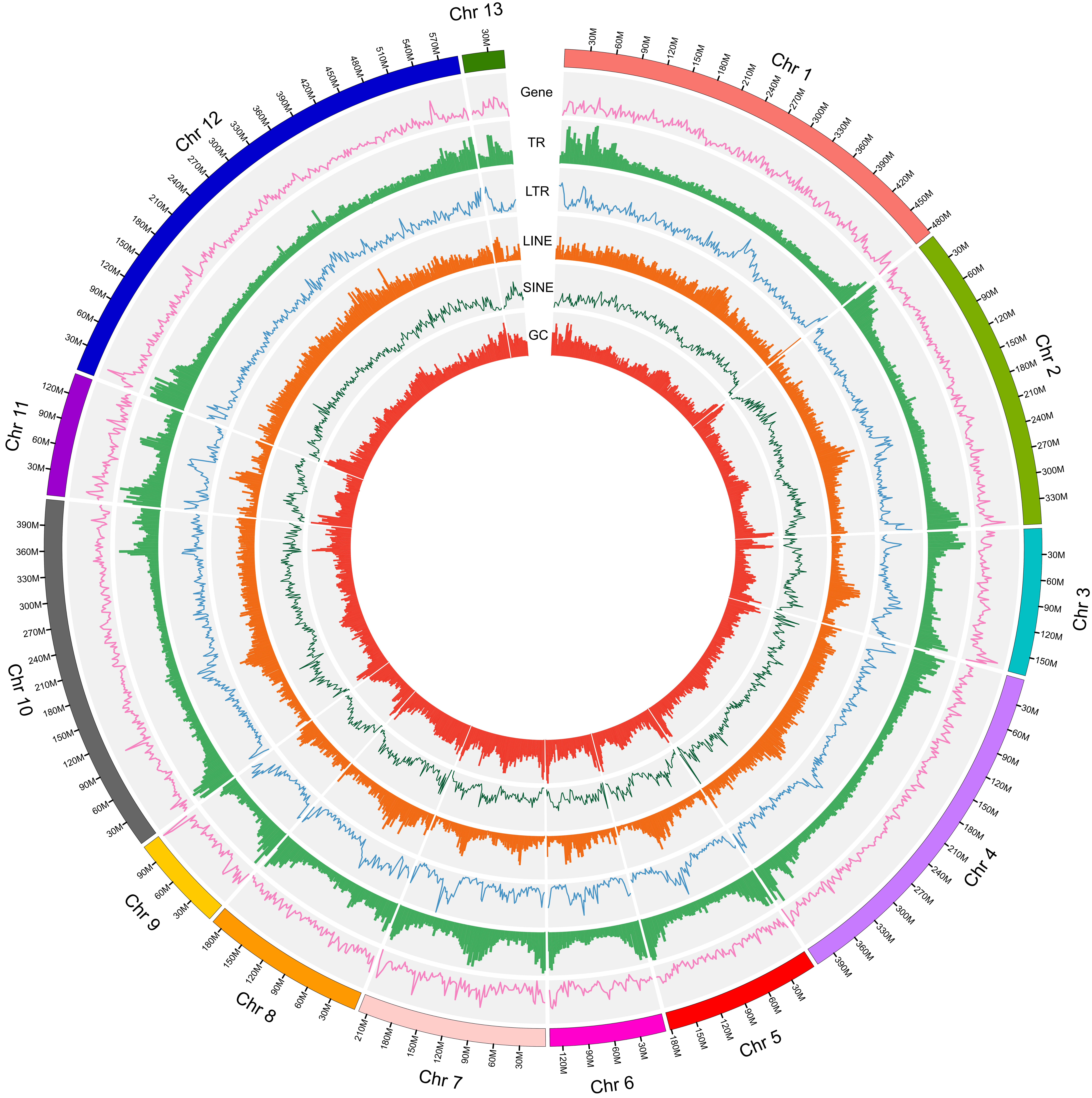

Figure 4

Click here to access/download;Figure;Figure 4.pdf

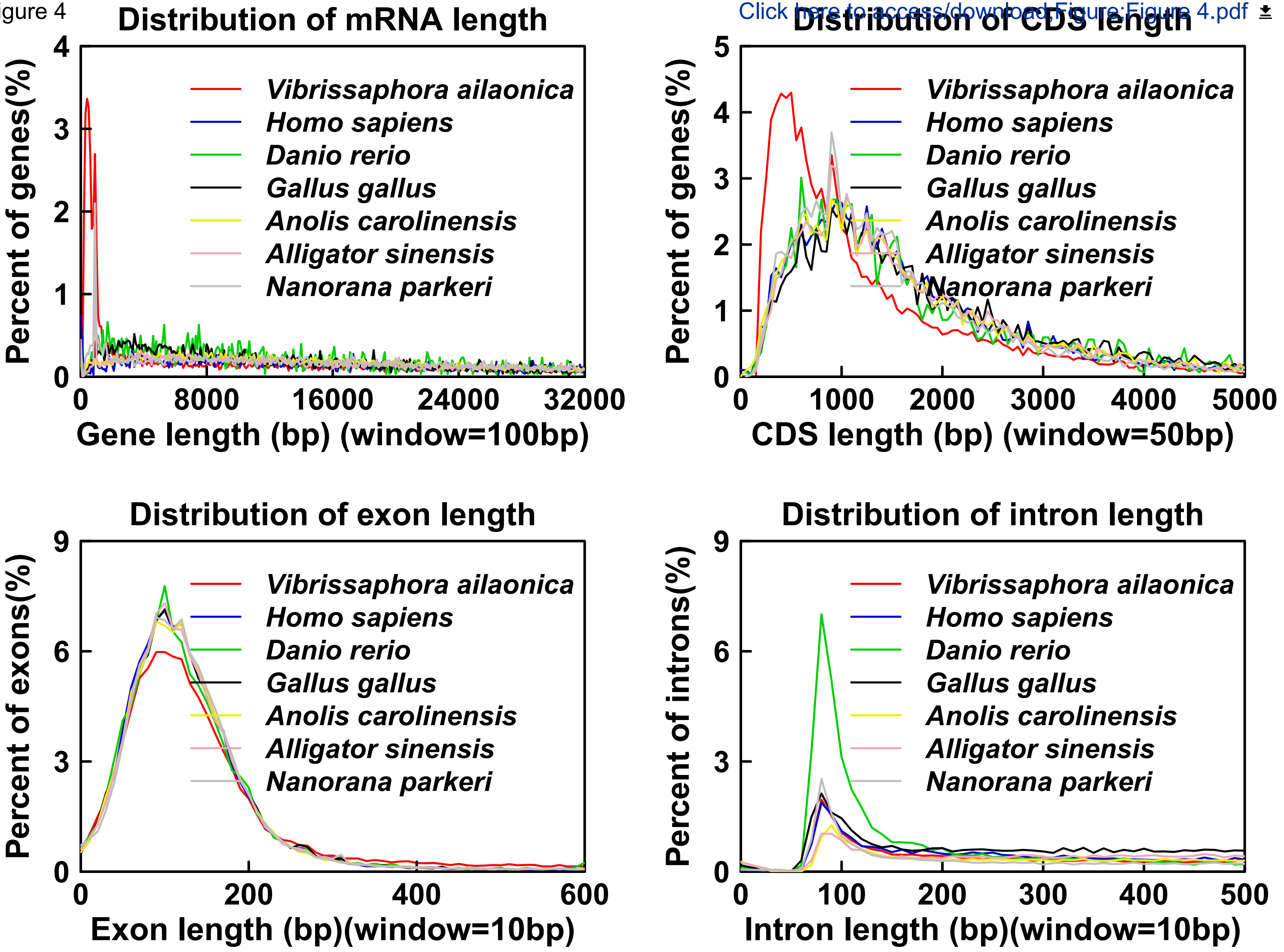

Figure 5

Click here to access/download;Figure;Figure 5.pdf

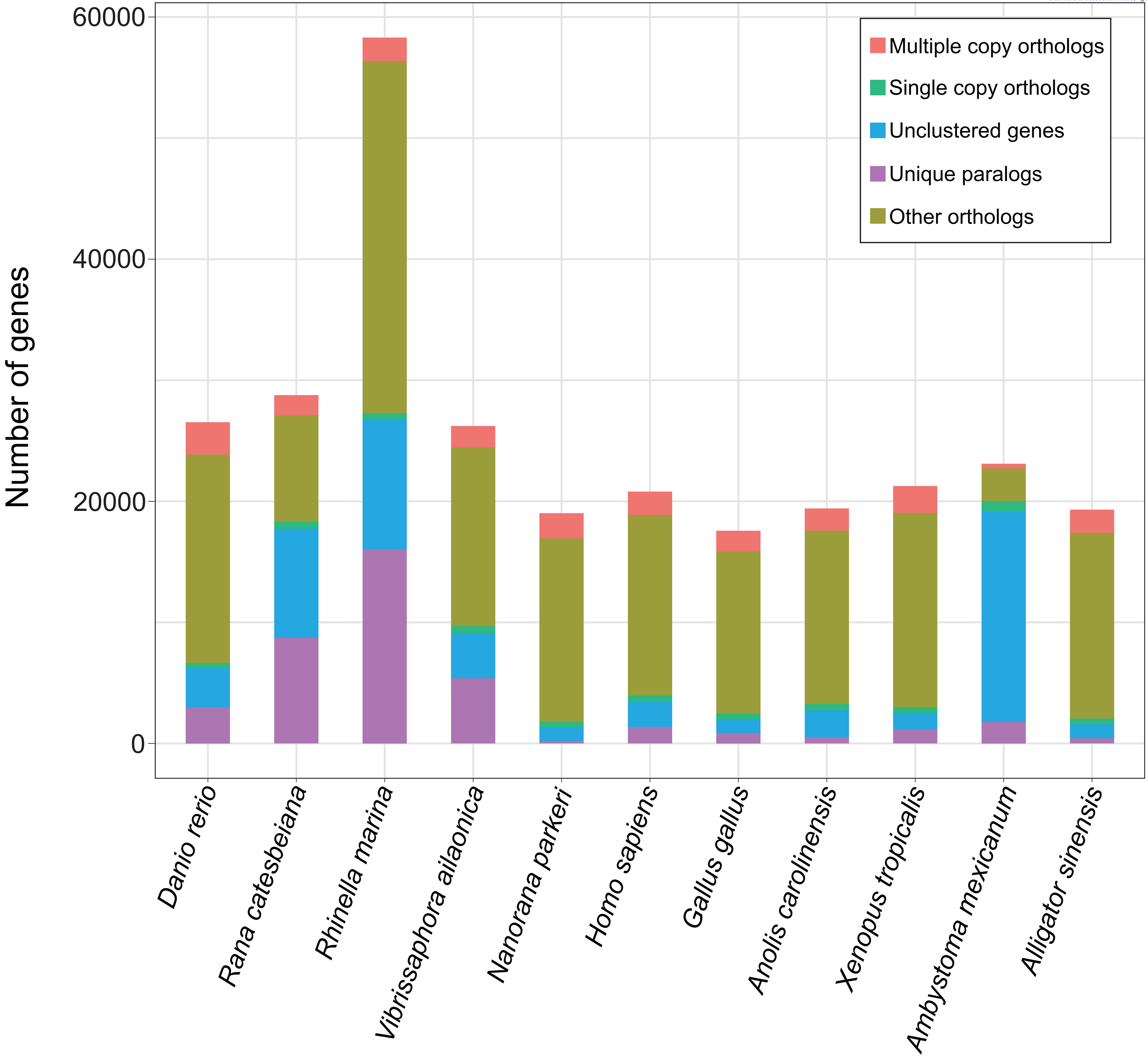

Figure 6

Click here to access/download;Figure;Figure 6.pdf

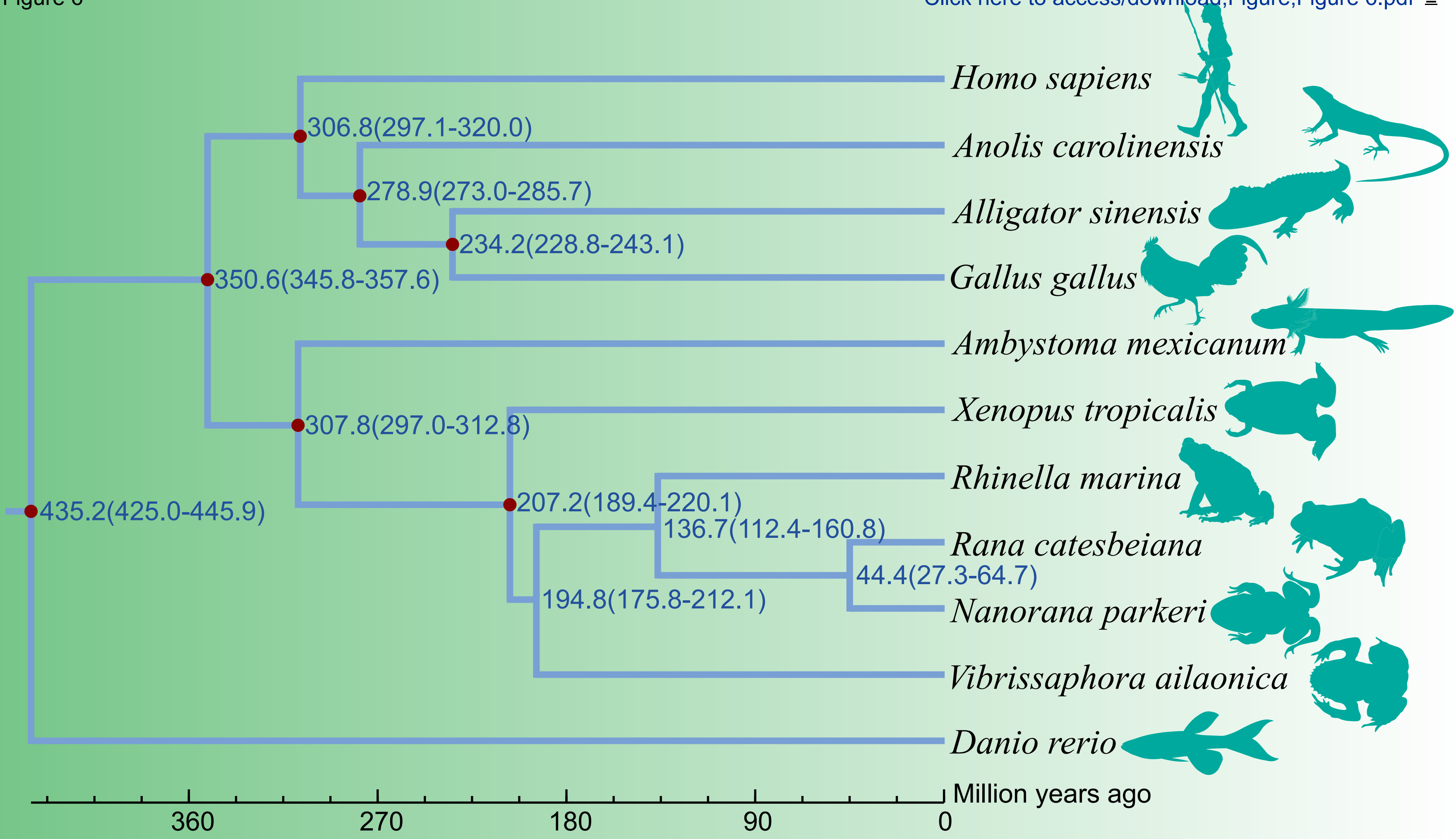

Click here to access/download
Supplementary Material
supplementary materials.doc

Personal Cover Click here to access/download;Personal Cover;cover letter.doc

Dear Editors,

We would like to submit the enclosed manuscript, entitled "**Chromosomal-level assembly of the mustache toad genome using third-generation DNA sequencing and Hi-C analysis**" for your consideration of publication as an original research paper in "***GigaScience***".

The mustache toad, *Vibrissaphora ailaonica*, belongs to the Megophryidae family and is an endemic amphibian species to China (partly across China-Vietnam border). The mustache toad has many interesting features, including the keratinized spines along the jaw. These spines repeatedly grow in sexually mature males (but not females) during the breeding season and fall off at the end of this process. Another unique aspect of the mustache toad is that breeding occurs during the cold season, unlike most frogs which breed in the warmer months. However, despite the importance of the mustache toad due to its unique morphological differences, such as spine development, genomic resources remain limited.

Using Illumina, PacBio, and Hi-C sequencing technologies, we reported on the first chromosomal-level genome assembly of the mustache toad. We successfully annotated 26,227 protein-coding genes by integrating three different methods. Phylogenetic analysis indicated that the mustache toad has a close relationship with the marine toad, bull frog, and Tibetan frog, and diverged from their common ancestor ~194.8 MYA. Analysis also indicated that the mustache toad had a faster evolutionary rate relative to most other closely related species examined. Gene family analysis identified several enriched biological processes and pathways, such as metabolism and intermediate filaments, suggesting that these terms may relate to the adaptation of the mustache toad to its habitat.

We believe these findings will be of interest to a broad audience of evolutionary and developmental biologists, as well as to researchers working to understand the molecular basis of keratinized spine development in the mustache toad. In addition, these genomic resources could help scientists studying the development and ecological adaptation of the mustache toad.

As a premier international journal devoted to the rapid dissemination of significant biological findings, ***GigaScience*** represents the ideal platform for sharing these results with the international research community.

We thank you for your consideration of our manuscript and look forward to hearing from you at your earliest convenience.

Yours sincerely,

Dingqi Rao, Ph.D

State Key Laboratory of Genetic Resources and Evolution, Kunming Institute of Zoology, Chinese Academy of Sciences, Kunming 650223, China

Email: kizar@mail.kiz.ac.cn
