# Supplementary material for: Chromosome-level assembly of the mustache toad genome using third-generation DNA sequencing and Hi-C analysis
Source: Gigascience. 2019 Sep 23;8(9):giz114. doi: 10.1093/gigascience/giz114 (PMC6755253; doi:10.1093/gigascience/giz114)
Supplement: giz114_GIGA-D-19-00099_Revision_1 [file giz114_giga-d-19-00099_revision_1.pdf]

GigaScience

Chromosomal-level assembly of the mustache toad genome using third-generation DNA sequencing and Hi-C analysis

--Manuscript Draft--

**Manuscript Number:**	GIGA-D-19-00099R1
**Full Title:**	Chromosomal-level assembly of the mustache toad genome using third-generation DNA sequencing and Hi-C analysis
**Article Type:**	Data Note
**Funding Information:**	National Key Research and Development Program of China (2017YFC0505202) — Dr. Dingqi Rao<br>National Natural Science Foundation of China (30270175) — Dr. Dingqi Rao<br>National Natural Science Foundation of China (30870278) — Dr. Dingqi Rao<br>National Natural Science Foundation of China (31372165) — Dr. Dingqi Rao
**Abstract:**	Background<br><br>The mustache toad, Vibrissaphora ailaonica, is an endemic species to China belonging to the Megophryidae family. Like other mustache toad species, V. ailaonica males temporarily develop keratinized nuptial spines on their upper jaw during each breeding season that fall off when the breeding season ends, which probably reversed the sexual size dimorphism with males being larger than females. To investigate the genetic mechanism of the repeatedly developed keratinized spines, a high-quality reference genome of mustache toad would be a valuable resource.<br><br>Findings<br><br>For genome construction, we generated 225 Gb of short reads and 277 Gb of long reads using Illumina and Pacific Biosciences (PacBio) sequencing, respectively. The sequencing data were assembled into a 3.53 Gb genome assembly with a contig N50 length of 821 Kb. Additionally, we applied Hi-C technology to identify contacts among contigs, then assembled contigs into scaffolds and identified a genome assembly with 13 chromosomes and a scaffold N50 length of 412.42 Mb. Based on the 26,227 protein-coding genes annotated in the genome, we analyzed the phylogenetic relationships of the mustache toad with other chordate species. Results showed that the mustache toad has a relatively higher evolutionary rate and separated from the marine toad, bull frog, and Tibetan frog ancestor 206.1 million years ago. Furthermore, we identified 201 expanded gene families in the mustache toad, which were mainly enriched in immune pathway, keratin filament, and metabolic processes.<br><br>Conclusions<br><br>Using Illumina, PacBio, and Hi-C technologies, we constructed the first high-quality chromosomal-level mustache toad genome. This work not only offers a valuable reference genome for functional studies of mustache toad traits, but also provides important chromosome information for wider genome comparisons.
**Corresponding Author:**	Dingqi Rao, Ph.D<br>Kunming Institute of Zoology Chinese Academy of Sciences<br>Kunming, Yunnan CHINA
**Corresponding Author Secondary Information:**	
**Corresponding Author's Institution:**	Kunming Institute of Zoology Chinese Academy of Sciences

Powered by Editorial Manager® and ProduXion Manager® from Aries Systems Corporation

**Corresponding Author's Secondary Institution:**	
**First Author:**	Yongxin Li
**First Author Secondary Information:**	
**Order of Authors:**	Yongxin Li
	Yandong Ren
	Dongru Zhang
	Hui Jiang
	Zhongkai Wang
	Xueyan Li
	Dingqi Rao, Ph.D
**Order of Authors Secondary Information:**	
**Response to Reviewers:**	Responses to Reviewers<br><br>The authors would like to thank the reviewers for the helpful comments, and we have revised our manuscript accordingly. At this time, we hope to have an opportunity to publish this paper in GigaScience.<br><br>Reviewer 1 Comments for the Author...<br><br>Reviewer #1: Li et al report the first genome assembly of a mustache toad. They used a combination of PacBio and HiC to generate a highly-contiguous assembly.<br>They used RNA-seq data, ab intio gene prediction and homology to annotate ~26000 genes, analyzed gene family contractions and expansions, and estimated the phylogenetic relationship to other amphibians. Given the sparsity of amphibian genomes, this assembly will be valuable for the community. I recommend accepting the manuscript after a few issues have been addressed, most of which are minor.<br><br>Major comments:<br><br>1) Since k-mer based genome size estimation is often not very precise, I find the redundancy reduction of the assembly potentially problematic. The authors removed contigs that overlap with at least 70% another contig using an alignment identity cutoff of 70%. It feels a bit like these parameters were optimized such that the final assembly matches the k-mer predicted size. E.g. the 70% identity cutoff is not compatible with the error rate of PacBio reads, unless the purpose of the Redundans run was to remove single reads that contain much more than the ~15% expected error rate. Also, heterozygosity and alt haplotypes should not result in 30% divergence.<br>I wonder if the authors can check which contigs were removed in this step and ensure that no real sequences were removed. If there is any doubt that some of the contigs may contain functionally important sequences (genes, etc), then I would suggest to provide the removed contigs with the redundancy-filtered assembly as an extra fasta file. Specifically, I wonder if the slightly lower BUSCO scores can be explained by removing real contigs based on 70% similarity.<br><br>Response: To make sure all the removed contigs not contain real sequences, we checked the BUSCO of the raw genome and the redundancy-filtered genome with eukaryote and metazoan as datasets at the same time. Besides, we also checked the mapping ratio of Illumina reads on the raw genome and the redundancy-filtered genome. The BUSCO results shown that most of the genes were not removed and the mapping ratio results shown that both coding region and non-coding region were remained (Table S5). All these results indicated that the redundancy-filtered step not removed many real sequences. However, as you have said before, we further checked the contigs that were removed in this step, all the removed contigs, the BUSCO results of genome and the redundancy-filtered assembly have been uploaded to GigaDB FTP server. Thank you for your suggestions.

Powered by Editorial Manager® and ProduXion Manager® from Aries Systems Corporation

2) The manuscript 'undersells' the contiguity of mustache toad assembly, which has *substantially higher* contig and scaffold N50 values than any other amphibian genome.
I therefore recommend to place Table S8 in the main text.

Response: Done. Table S8 has been removed to main text (Table 4). Thank you.

3) Table 3 is hard to understand as absolute numbers are reported. A much better way would be to report '%complete genes, %complete and duplicated genes, %fragmented genes, %missing genes' which sums to 100%. In addition, these 4 BUSCO percentages for the other amphibian genomes should be added to this table to provide a direct comparison of genome assembly completeness.

Response: Table 3 has been corrected and the BUSCO results of the other amphibian genomes were also added.

4) I wonder how the divergence time estimates would change if first or second codon positions instead of four-fold degenerate sites were used. This may be relevant as four-fold degenerate sites are clearly saturated over these phylogenetic distances. Also, the divergence times shown in Figure 6 are quite different to the times from timetree, where e.g. the Rana - Nanorana split was 89 Mya (Figure 6, 44 Mya) and the Rana - Rhinella split was 160 Mya (Figure 6, 137 Mya).

Response: We added the fossil evidence between Rana - Nanorana and Rana - Rhinella from timetree to run mcmctree tree again, and both four-fold degenerate sites and first or second codon positions were used for the divergence time analysis (Figure 6; Figures S2 and S3). The results shown that these three results are much closed and the divergence time between Rana - Nanorana, Rana - Rhinella are similar to timetree results. We have revised the divergence time results in Figure 6. Thank you.
Besides, because the expansion and contraction analysis of gene family are related the divergence time result, so we updated the expansion and contraction results this time, including all the descriptions and Tables (Tables S11-S14). Thank you.

Minor comments:
1) The manuscript should be edited by a native speaker to improve the language.
A few examples: "Like other mustache toad species, V. ailaonica males develop temporary keratinized nuptial spines on their upper jaw during each breeding season and fall off when the breeding season ends, which probably lead to the reverse of the sexual size dimorphism, namely the size of the male get larger than female." should be improved to "Like other mustache toad species, V. ailaonica males temporarily develop keratinized nuptial spines on their upper jaw during each breeding season that fall off when the breeding season ends, which probably reversed the sexual size dimorphism with males being larger than females."
"To investigate the genetic mechanism of the repeatedly develop the keratinized spines"
-->
"To investigate the genetic mechanism of the repeatedly developed keratinized spines"

"Another unique aspect of the mustache toad is that breeding occurs during the cold season, unlike most frogs and toads which breed in the warmer months"
-->
"Another unique aspect of the mustache toad is that breeding occurs during the cold season, whereas most frogs and toads breed in the warmer months"
etc.

Response: Done. We have corrected the above mistakes and other mistakes in our manuscript. Thank you.

2) Please reference Figure 1 in line 55, where the temporary spines are described.

Response: Corrected.

3) Line 75-76: I find this outlook that we will learn from the toad genome (sex

dimorphism) how body size control works in general a bit far-stretched. This could be removed.

Response: Corrected.

4) Line 94/95: Please mention the Illumina read length (paired end 150 bp reads). I find this information more important than library size.

Response: Corrected.

5) Line 164/165: The conclusion that the toad assembly is very complete is justified based on the high percentage of mapping RNA-seq reads and transcripts. However, this sentence should be moved to Line 161 (after "Table S8).", where this analysis is done.

Response: Corrected.

6) Line 180: Please replace 'closely-related' with 'vertebrate' as zebrafish, lamprey and amphibians are not really closely related.

Response: Corrected.

7) Line 179: Would an Augustus model trained from an amphibian (e.g. xenopus) be not more appropriate than a zebrafish model?

Response: The official website of Augustus not included the amphibian species as model. So we selected zebrafish that has high-quality gene set as model. Thank you for your suggestions.

8) Table 4: Please round the percentage to 2 digits (9.94%).

Response: Corrected.

9) Line 204/205: The references don't match: Reference 34 (www.axolotl-omics.org) and 36 refer to the Ambystoma genome assembly. The Rhinella reference is missing.

Response: Corrected.

Reviewer 2 Comments for the Author...

Reviewer #2: Here, Yongxin Li and colleagues reported the chromosome-level genome with the full annotation of the mustache toad, Vibrissaphora ailaonica, using conventional paired-end short read, sufficient amount of PacBio long reads and chromosome conformation capture (Hi-C) data. Although there are several amphibian genomes reported previously, many of them do not have chromosome-level genomes, so I think this is definitely a valuable resource to the community, especially to study the synteny of amphibian genome. So I would like to recommend accepting this manuscript for publication after resolving some issues as mentioned below:

1) On page 5, more details for RNA-Seq library prep construction method should be provided (poly-A capturing or ribosome-depletion? Which library kit do they use?). Also, even the authors mentioned that 9 tissues were dissected from the biospecimen they sequenced the genome (page 4, line 85), it is not clear whether all those tissues were used in this 'mixed RNA-Seq' experiment. Please provide more details for this experiment.

Response: The datails for RNA-seq library construction method were added. About the RNA-seq experiment, after equally mixed the DNA of the 9 tissues, the mixed DNA sample was used for library construction and RNA-seq experiments. Thank you.

2) On page 5, the authors mentioned that they used four Hi-C libraries. Are they constructed from the same samples (blood), with the same parameter (four technical replicates)? Or using different samples? If they used a different parameter to construct these four libraries, it should be specified.

Powered by Editorial Manager® and ProduXion Manager® from Aries Systems Corporation

	Response: Yes, all these four Hi-C libraries were used the same samples with the same parameter. We also clarify these informations in the main text this time. Thank you for your suggestions.<br><br>3) Authors claimed that they deposited the data on PRJNA523649, but it looks like they uploaded one single file for each data set. Because they used different libraries, at least for paired-end seq (Table S1) and HiC-seq (Table S4), it would be better to provide those raw data separately.<br><br>Response: Thank you for your suggestions, we uploaded these sequencing data in the same PROJECT ID, but with different SRA IDs, you could see this by this link (https://www.ncbi.nlm.nih.gov/sra/?term=PRJNA523649). Thank you.
**Additional Information:**	
**Question**	**Response**
Are you submitting this manuscript to a special series or article collection?	No
**Experimental design and statistics**<br><br>Full details of the experimental design and statistical methods used should be given in the Methods section, as detailed in our Minimum Standards Reporting Checklist. Information essential to interpreting the data presented should be made available in the figure legends.<br><br>Have you included all the information requested in your manuscript?	Yes
**Resources**<br><br>A description of all resources used, including antibodies, cell lines, animals and software tools, with enough information to allow them to be uniquely identified, should be included in the Methods section. Authors are strongly encouraged to cite Research Resource Identifiers (RRIDs) for antibodies, model organisms and tools, where possible.<br><br>Have you included the information requested as detailed in our Minimum Standards Reporting Checklist?	Yes
**Availability of data and materials**	Yes

All datasets and code on which the conclusions of the paper rely must be either included in your submission or deposited in publicly available repositories (where available and ethically appropriate), referencing such data using a unique identifier in the references and in the "Availability of Data and Materials" section of your manuscript.<br><br>Have you have met the above requirement as detailed in our Minimum Standards Reporting Checklist?	

Manuscript
Click here to access/download;Manuscript;Manuscript.doc
Click here to view linked References

Chromosomal-level assembly of the mustache toad genome using third-generation DNA sequencing and Hi-C analysis

Yongxin Li[1,2,†], Yandong Ren[2,†], Dongru Zhang[1,†], Hui Jiang[3], Zhongkai Wang[2], Xueyan Li[1], Dingqi Rao[1,*]

1. State Key Laboratory of Genetic Resources and Evolution, Kunming Institute of Zoology, Chinese Academy of Sciences, Kunming 650223, China
2. Center for Ecological and Environmental Sciences, Northwestern Polytechnical University, Xi'an 710072, China
3. National Engineering Laboratory of Marine Germplasm Resources Exploration and Utilization, Zhejiang Ocean University, Zhoushan 316022, China

[†]These authors have the equal contribution.
[*]Corresponding author: Dingqi Rao (kizar@mail.kiz.ac.cn).

Abstract

Background: The mustache toad, *Vibrissaphora ailaonica*, is an endemic species to China belonging to the Megophryidae family. Like other mustache toad species, *V. ailaonica* males temporarily develop keratinized nuptial spines on their upper jaw during each breeding season that fall off when the breeding season ends, which probably reversed the sexual size dimorphism with males being larger than females. To investigate the genetic mechanism of the repeatedly developed keratinized spinesLike other mustache toad species, *V. ailaonica* males develop temporary keratinized nuptial spines on their upper jaw during each breeding season and fall off when the breeding season ends, which probably lead to the reverse of the sexual size dimorphism, namely the size of the male get larger than female. To investigate the genetic mechanism of the repeatedly develop the keratinized spines, a high-quality reference genome of mustache toad would be a valuable resource. **Findings:** For genome construction, we generated 225 Gb of short reads and 277 Gb of long reads using Illumina and Pacific Biosciences (PacBio) sequencing, respectively. The sequencing data were assembled into a 3.53 Gb genome assembly with a contig N50 length of 821 Kb. Additionally, we applied Hi-C technology to identify contacts among contigs, then assembled contigs into scaffolds and identified a genome assembly with 13 chromosomes and a scaffold N50 length of 412.42 Mb. Based on the 26,227 protein-coding genes annotated in the genome, we analyzed the phylogenetic relationships of the mustache toad with other chordate species. Results showed that the mustache toad has a relatively higher evolutionary rate and separated from the marine toad, bull frog, and Tibetan frog ancestor 206.1194.8 million years ago. Furthermore, we identified 201349 expanded gene families in the mustache toad, which were mainly enriched

in immune pathway, keratin filament, and metabolic processes. **Conclusions:** Using Illumina, PacBio, and Hi-C technologies, we constructed the first high-quality chromosomal-level mustache toad genome. This work not only offers a valuable reference genome for functional studies of mustache toad traits, but also provides important chromosome information for wider genome comparisons.

Keywords: Mustache toad; Genome assembly; Evolution; PacBio; Hi-C

Introduction

The mustache toad, *Vibrissaphora ailaonica* (NCBI Taxonomy ID: 428466), belongs to the Megophryidae family and is an endemic amphibian species to China (including the China-Vietnam border) [1-3]. This mustache toad species exhibits many interesting features, including unique keratinized spines along the upper jaw [1, 4-6]. These spines repeatedly grow in sexually mature males during the breeding season and fall off at the end of this process [5-8] (Figure 1). This morphological difference between males and females is further highlighted by their sexual dimorphism in body size (males are significantly larger than females) and may be used as a weapon for sexually mature individuals to compete for nests and mating opportunities [7, 9, 10]. Another unique aspect of the mustache toad is that breeding occurs during the cold season, whereas most frogs and toads breed in the warmer months ~~Another unique aspect of the mustache toad is that breeding occurs during the cold season, unlike most frogs and toads which breed in the warmer months~~ [1]. However, despite the importance of the mustache toad in spine dynamic development and sexual dimorphism in

body size, the genomic resources for the species remain limited. To date, no next-generation sequencing (NGS) data have been reported in the *Vibrissaphora* genus. Therefore, this lack of genome sequence and transcriptome data for this important species (*V. ailaonica*) has hindered identification of genome-based functional genes related to their attractive dynamic body appearance (e.g., spine and body size). Besides, as there is such a shortage of amphibian genomes in Genome 10K project, and it is necessary to analyze other important genomes to large-scale study the phylogenetic relationships in amphibian [11].

In this study, we combined genomic sequencing data from Illumina short reads, PacBio long reads, and Hi-C data to generate the first chromosomal-level reference genome for the mustache toad. The completeness and continuity of the genome were comparable with that of other important amphibian species. The high-quality reference genome generated in this study will facilitate research on population genetic traits and functional gene identification related to important characteristics of the mustache toad~~, which will, in turn, accelerate the development of more efficient body size control techniques and improve the artificial breeding industry for other economically important species~~.

Sampling and sequencing

A male mustache toad (*V. ailaonica*) with keratinized nuptial spines on its upper jaw was caught from the Ailao Mountain during the breeding season for sequencing (Figure 1). To obtain sufficient high-quality DNA for the PacBio Sequel platform (Pacific Biosciences, USA), the mustache toad was dissected, and fresh liver tissue was used for DNA extraction using phenol/chloroform extraction. DNA quality was checked by agarose gel electrophoresis,

with excellent integrity DNA molecules were obtained. Other tissues, including spines, brain, stomach, intestine, liver, lung, spleen, blood, and tongue, were snap-frozen in liquid nitrogen for 10 min and then all these 9 organs/tissues were stored at −80℃ for~~–~~ RNA-seq experiment~~subsequent use~~. Isolated total RNA were used to isolate intact poly(A)+ RNA by the NEBNext Poly(A) mRNA Magnetic Isolation Module. The mRNA was further fragmented and randomly primed during the first-strand synthesis by reverse transcription. This procedure was followed by second-strand synthesis with DNA polymerase I to create double-stranded cDNA fragments using Transcriptor First Strand cDNA Synthesis Kit (Roche). In the Hi-C experiments, the collected blood was used for library construction. The blood sample (150 ul) was cross-linked for 10 min with formaldehyde (1% final concentration), after which glycine (0.2 M final concentration) was added for 5 min to stop the cross-linking process, with the sample then stored until the further analysis.

Extracted DNA was sequenced using the Illumina and PacBio Sequel platforms. The short reads generated from the Illumina platform were used for estimation of genome size and error correction of the assembled genome, and the PacBio long reads were used for genome assembly. To this end, five libraries with insertion lengths of 220 bp or 500 bp were ~~generated from~~sequenced on ~~the~~ Illumina HiSeq 2500 platform generating 150 bp paired-end reads and a 20 Kb library was constructed using the PacBio platform according to the manufacturers' protocols. Finally, we obtained 225.03 Gb of Illumina short reads and 277.15 Gb of PacBio long reads (Table 1; Additional File: Tables S1 and S2). The average subreads N50 length reached to 14.78 Kb, providing ultra-long genomic sequences for the following assembly and analysis (Additional File: Table S2). The RNA-seq samples were obtained by mixing an equal

amount of RNA extracted from each tissue stored and used for library construction. After sequencing on the Illumina HiSeq 4000 platform, we obtained 14.18 Gb of sequencing data (Table 1; Additional File: Table S3). Four Hi-C libraries were constructed using the same sample with same parameters and sequenced on the Illumina Hiseq X-ten platform, which generated 378.78 Gb of clean data (Table 1; Additional File: Table S4).

Genome characteristics estimation

The Illumina short reads were quality filtered by the following steps: First, the adaptors were removed from the sequencing reads. Second, read pairs were excluded if any one read had more than 10% "N". Third, read pairs with low quality base more than 50% were removed. Fourth, the PCR duplicates produced during library construction in read pairs were removed. The filtered reads were used for estimation of genome size and other characteristics. Using the k-mer method, we calculated the 17-mer depth frequency distribution in the mustache toad. Genome size was estimated by: $G = TKN_{17\text{-mer}} / PKFD_{17\text{-mer}}$, where $TKN_{17\text{-mer}}$ is the total kmer number and $PKFD_{17\text{-mer}}$ is the peak kmer frequency depth of 17-mer. We estimated a genome size of 3.52 Gb (peak = 54) and found a heterozygous and repeat sequences peak, suggesting that the mustache toad genome exhibits complex genome assembly (Figure 2).

Genome assembly by PacBio long reads and Hi-C data

Based on 38 single-molecular real-time cells by the PacBio Sequel platform, we generated 277.15 Gb of subreads (Table 1; Additional File: Table S2). The average and N50 length of subreads were 9.65 Kb and 14.78 Kb, respectively (Additional File: Table S2). All the long

reads were used for genome assembly using wtdbg software (https://github.com/ruanjue/wtdbg-1.2.8). As a result, we obtained a 3.95 Gb genome assembly with a contig N50 length of 739.54 Kb. However, although the size of the genome assembly was comparable with the estimation k-mer result, it was a slightly larger. This may be due to the complexity of the mustache toad genome (high heterozygous rate and repeat sequences). Then the redundant sequences in the genome assembly were removed using Redundans software (v0.13c) [12] with an identity of 0.7 and overlap of 0.7, resulting in a genome assembly of 3.58 Gb and contig N50 length of 834.90 Kb. To make sure all the removed contigs not contain real sequences, we checked the BUSCO result and the mapping ratio of Illumina reads in the raw genome and the redundancy-filtered genome. Finally, All these results indicated that the parameters in the redundancy-filtered step are proper in this study (Additional File: Table S5). To further improve the quality and accuracy of our genome assembly, the Illumina short reads were used to polish the genome using Pilon software (RRID:SCR_014731, v1.21) [13] at the single-base level. The Hi-C data were used to improve the connection integrity of the contigs (15,899 contigs). We obtained 378.78 Gb of Hi-C sequencing data, which were first filtered by Hic-Pro (v2.10.0) [14] (Table 1; Additional File: Table S4), and then mapped to the polished mustache toad genome [15]. The location and direction of the contigs were determined by 3D *de novo* assembly (3d-DNA) software (v180419) [16] with default parameters. Most contigs were then successfully clustered and anchored on 13 groups (Figure 3) [17]. Finally, we obtained the first chromosomal-level high quality mustache toad assembly (3.53 Gb) with a scaffold N50 length of 412.42 Mb, providing a solid genomic resource for further study of the mustache toad (Table 2).

Genome assembly evaluation

Genome assembly quality is directly related to the accuracy and completeness of protein-coding gene prediction. Therefore, we evaluated the assembled mustache toad genome using three methods. First, the assembled genome was compared with the core gene set in BUSCO software (RRID:SCR_015008, v2.0) [18]. We found 245 (80.8%) and 833 (85.1%) conserved core genes in the mustache toad genome using the eukaryote and metazoan databases, respectively (Table 3). When we further considered the fragmented BUSCO genes found in the genome, there were 272 (89.7%) and 881 (90.1%) conserved core genes found in the eukaryote and metazoan databases, respectively. ~~(Table 3)~~ This results indicated that the assembled mustache toad genome is comparable with published amphibian genomes (Table 3). Second, we aligned all filtered short reads generated from the Illumina platform to the genome using BWA software (RRID:SCR_010910, v0.7.12) [19] and found 1,778 million clean reads that could be mapped to the genome, accounting for 97.78% of total clean reads (Additional File: Table ~~S5~~S6). Third, the RNA-seq reads were *de novo* assembled using Bridger software (RRID:SCR_017039, version: r2014-12-01) [20], with redundant transcripts removed by TGICL [21], resulting in 19,876 transcripts (Additional File: Table ~~S6~~S7). These transcripts were then aligned to the genome, with 17,878 transcripts (89.95%) found in the assembled genome and 94.52% of transcripts longer than 1 Kb (Additional File: Table ~~S7~~S8). Besides, we analyzed the N50 length and BUSCO results and found that the mustache toad genome was comparable with other published amphibian genomes (Tables 2-~~and Table 3~~-4~~; Additional File: Table S8~~). These results indicate that our assembled mustache

toad genome exhibited high completeness and accuracy.

The GC distribution of the mustache toad and vertebrate ~~closely related~~ species was calculated using the slide window method. Results showed that their GC distributions were similar to each other, with an average GC content of 43.68% in the mustache toad and 36.60% to 44.49% in other species (Additional File: Figure S1).~~ These results indicate that our assembled mustache toad genome exhibited high completeness and accuracy.~~

Genome annotation

We used Tandem Repeat Finder (TRF, v4.04) [22] to identify repetitive elements and RepeatModeler software (RRID:SCR_015027, v1.0.4) to detect transposable elements (TEs) in the mustache toad genome. Then, the *de novo* library of repeats produced by RepeatModeler analysis and the repbase (RepBase16.02) database were then used for RepeatMasker (RRID:SCR_012954, version: open-4.0) [23] analysis to identify homologous repeats. RepeatProteinMask was used to query the TE protein database at the protein level. Lastly, we identified 2.45 Gb of repeat sequences, which accounts for 69.48% of the estimated genome size (Additional File: Table S9). Among these repeat sequences, 60.87% (2.15 Gb) was predicted by the *de novo* method (Table ~~4~~5).

After repeat sequence annotation, we masked all repeats, except for the tandem repeat sequences, for protein-coding gene annotation. Augustus software (RRID:SCR_008417, v2.5.5) [24] was used to *de novo* predict coding genes using a zebrafish (*Danio rerio*) dataset as the train species. For the homology-based method, protein sequences of ~~closely related~~chordate species, including *D. rerio* (GCF_000002035.6) [25], *Nanorana parkeri*

(GCF_000935625.1) [26], *Homo sapiens* (GCF_000001405.38) [27], *Gallus gallus* (GCF_000002315.5) [28], *Pelodiscus sinensis* (GCF_000230535.1) [29], *Xenopus laevis* (GCF_001663975.1) [30], and *Petromyzon marinus* [31] (https://genomes.stowers.org/organism/Petromyzon/marinus), were downloaded and aligned against the mustache toad genome using the TBLASTN module (BLAST version: 2.3.0). The transcripts assembled by RNA-seq reads were first translated into amino acids and then aligned to the genome using TBLASTN software for gene annotation. EVidenceModeler (RRID:SCR_014659, version: r2012-06-25) [32] was used to integrate results from the three methods, and genes with poor transcriptome evidence support were filtered out. Finally, 26,227 high-quality protein-coding genes were predicted in the mustache toad genome. Moreover, the distributions of mRNA, CDS, exon, and intron lengths were comparable with closely related species (Figure 4).

Gene functional annotation can help to elucidate gene function. Thus, we aligned all 26,227 protein-coding genes to protein databases, including InterProScan, KEGG, SwissProt, and TrEMBL. Results showed that most obtained genes could be annotated in these functional databases (Table ~~5~~6).

Phylogenetic tree and divergence time analysis

To reveal the phylogenetic relationships of the mustache toad with other closely related species, we identified the single-copy genes among the species. First, protein sequences, including those of *D. rerio* (GCF_000002035.6) [25], *N. parkeri* (GCF_000935625.1) [26], *H. sapiens* (GCF_000001405.38) [27], *G. gallus* (GCF_000002315.5) [28], *Anolis carolinensis*

(GCF_000090745.1) [33], *Xenopus tropicalis* (GCF_000004195.3) [30], *Rhinella marina* (GigaDB) [34], *Rana catesbeiana* (GCA_002284835.2) [35], *Ambystoma mexicanum* (www.axolotl-omics.org) [36, 37], and *Alligator sinensis* (GCF_000455745.1) [~~37~~38], were downloaded from NCBI and the longest transcript of each gene in each species was selected. The BLASTP program (BLAST version: 2.2.24) was then used to align these protein sequences among these 11 species (including the mustache toad) with an e-value of 1e-5. The homolog relationships (including ortholog and paralog) were then determined using OrthoMCL software (v1.4) [~~38~~39]. Genes with only one copy in the species were identified as single-copy genes. In total, 238 genes were identified (Figure 5), with the detailed results of gene family statistics shown in the supplementary information (Additional File: Table S10). The 238 single-copy genes were aligned using MUSCLE software (RRID:SCR_011812, v3.8.31) [~~39~~40, ~~40~~41] and concatenated to supergenes for maximum-likelihood-based phylogenetic analyses. We performed phylogenetic analysis, with zebrafish as the outgroup, using RAxML software (RRID:SCR_006086, v8.2.3) [~~41~~42] with the parameter -m for PROTGAMMAAUTO. Results indicated that the mustache toad exhibited a close relationship with the ancestor of the marine toad (*R. marina*), bull frog (*R. catesbeiana*), and Tibetan frog (*N. parkeri*), with topological relationships in other clades found to be the same as reported previously (Figure 6). To further investigate the divergence time of these species, especially toad and frogs, the MCMCTREE model in PAML software (RRID:SCR_014932, v4.8) [~~42~~43] was used with three datasets (four-fold degenerate sites (4dTVs); the first-codon sites; the second-codon sites) extracted from the single-copy genes as the input file. Fossil records were downloaded from the TIMETREE website (www.timetree.org) and used to

calibrate the results. We found that the results from the three different datasets are very similar and the mustache toad diverged with the common ancestor of the marine toad, bull frog, and Tibetan frog about ~~194.8~~206.1 million years ago (Figure 6; Additional File: Figures S2 and S3).

Gene family expansion and contraction

We performed gene family expansion and contraction analysis using CAFÉ software (RRID:SCR_005983, v4.0) [~~43~~44], and found ~~349~~ 201 and ~~2,607~~326 expanded and contracted gene families in the mustache toad (*P* < 0.05), respectively. Using the GO/KEGG databases, functional enrichment analysis of the expanded gene families found ~~174~~ 210 GO terms (adjusted *P*-value < 0.05) and ~~2~~9 KEGG pathways (*q*-value < 0.05) to be significantly enriched (Additional File: Tables S11 and S12). The expanded gene families were mainly related to metabolic processes, intermediate filament terms, enzyme activities, and immune terms. For example, the cellular metabolic process (adjusted *P*-value = ~~2.37~~6.06E-14), intermediate filament (adjusted *P*-value = ~~3.92~~3.42E-1~~5~~3), keratin filament (adjusted *P*-value = ~~1.66~~2.94E-1~~3~~2), endoribonuclease activity (adjusted *P*-value = ~~1.06~~9.19E-0~~8~~7), and immune response (*q*-value = ~~4.81~~8.36E-0~~3~~6) were enriched (Additional File: Tables S11 and S12). In addition, for the contracted gene families, ~~226~~ 220 GO terms (adjusted *P*-value < 0.05) and ~~11~~ 9 KEGG pathways (*q*-value < 0.05) were enriched, respectively (Additional File: Tables S13 and S14). These enriched terms were mainly involved in ion binding and transporter activity, including neurotransmitter transporter activity (adjusted *P*-value = ~~4.20~~1.89E-~~11~~09), sodium ion transmembrane transporter activity (adjusted *P*-value =

~~1.55~~3.33E-06~~8~~), and secondary active transmembrane transporter activity (adjusted *P*-value = ~~6.37~~1.86E-08) (Additional File: Tables S13 and S14). Thus, these biological processes may be related to the special characteristics of the mustache toad.

Relative evolutionary rate of species

The evolutionary rate of species can reflect its evolution history and status. The relative evolutionary rate of the mustache toad to other closely related species was analyzed using LINTRE [~~44~~45] and MEGA (RRID:SCR_000667, v7.0.26) softwares. Two-cluster analysis was applied to test the molecular evolution of multiple sequences in a phylogenetic context based on the concatenated supergenes (protein sequences) using *tpcv* (a module in LINTRE software). The concatenated supergenes were also used for Tajima's relative rate test. We used zebrafish as the outgroup in both methods, and found that, except for the axolotl, the mustache toad had a relatively faster evolutionary rate than its closely related species (e.g., *X.tropicalis*, *R. marina*, *R. catesbeiana*, and *N. parkeri*) (Additional File: Tables S15 and S16). The crocodile had a slower evolutionary rate relative to its closely related species, and this result is consistent with previous study [~~45~~46] (Additional File: Tables S15 and S16).

Conclusions

Using Illumina, PacBio, and Hi-C sequencing technologies, we reported on the first chromosomal-level genome assembly of the mustache toad. We successfully annotated 26,227 protein-coding genes by integrating the results of three different methods. The phylogenetic analysis results indicated that the mustache toad has a close relationship with the marine toad,

bull frog, and Tibetan frog, and diverged at ~~194.8~~206.1 MYA with their common ancestor. Analysis showed that the mustache toad had a faster evolutionary rate relative to most other closely related species studied. Expansion and contraction of gene family analysis identified several biological processes and pathways, such as metabolism and intermediate filaments, suggesting that these terms may relate to the special adaptations of the mustache toad to its habitat.

Availability of supporting data

The raw sequencing data were deposited in the NCBI database under accession number PRJNA523649. The genome assembly and annotation results are available via the GigaScience repository GigaDB.

Additional files

Figure S1: The GC content in these genomes.

Figure S2: The divergence time of these species (using the first-codon sites).

Figure S3: The divergence time of these species (using the second-codon sites).

Table S1: The statistics of Illumina sequencing clean data.

Table S2: The statistics of PacBio Sequel sequencing data.

Table S3: The statistics of RNA-seq clean data.

Table S4: The statistics of Hi-C sequencing clean data.

Table S5. The comparison of BUSCO and Illumina reads mapping results in these two genome versions.

Table S65: The statistics of Illumina reads mapping ratio to the assembled genome.

Table S76: The statistics of assembled transcripts by Bridger software.

Table S87: The statistics of transcripts mapping ratio to the assembled genome.

~~Table S8: The quality statistics of several published amphibian genomes.~~

Table S9: The statistics of the annotated repeat sequences in our assembled genome.

Table S10: The statistics of gene family among these species.

Table S11: The GO enrichment analysis of expanded gene families.

Table S12: The KEGG enrichment analysis of expanded gene families.

Table S13: The GO enrichment analysis of contracted gene families.

Table S14: The KEGG enrichment analysis of contracted gene families.

Table S15: Two cluster analysis of mustache toad and other species.

Table S16: The relative evolutionary rate of mustache toad and other species analyzed by Tajima's Test.

Abbreviations

BLAST: Basic Local Alignment Search Tool; BUSCO: Benchmarking Universal Single-Copy Orthologs; BWA: Burrows-Wheeler Aligner; CDS: Coding DNA Sequence; DNA: Deoxyribonucleic Acid; GO: Gene Ontology; Hi-C: High-throughput chromosome conformation capture; KEGG: Kyoto Encyclopedia of Genes and Genomes; MHC: Major Histocompatibility Complex; NCBI: National Center for Biotechnology Information; NR: Non-Redundant Protein Sequence Database; PCR: Polymerase Chain Reaction; RNA:

Ribonucleic Acid; RNA-seq: RNA sequencing.

Conflicts of interest

The authors declare that they have no competing interests.

Funding

This work was supported by the National Key Research and Development Program of China (NO. 2017YFC0505202) and National Natural Science Foundation of China (NO. NSFC-30270175; NO. NSFC-30870278; NO. NSFC-31372165).

Author contributions

D.R. designed the project; D.R. and D.Z. collected the samples; Y.L. and Y.R. estimated the genome size and assembled the genome; Y.L. polished the assembled genome and employed the Hi-C analysis; H.J. performed the genome annotation; Y.L. and Z.W. assessed the quality of the genome assembly; Y.L. and Y.R. constructed the phylogenetic tree and determined divergence time, relative evolutionary rate of species, and expansion and contraction of gene families. Y.L., D.R., Y.R., and X.L. wrote the manuscript.

References

1. Liang F and Changyuan Y. Amphibians of China. Science Press: Beijing; 2016.
2. Matsui M, Hamidy A, Murphy RW, Khonsue W, Yambun P, Shimada T, et al. Phylogenetic relationships of megophryid frogs of the genus Leptobrachium (Amphibia, Anura) as revealed by mtDNA gene sequences. Molecular Phylogenetics & Evolution. 2010;56 1:259-72.
3. Matsui M. A New Leptobrachium (Vibrissaphora) from Laos (Anura: Megophryidae). Current Herpetology. 2013;32 2:182-9.

4. Liu C HS, Zhao EJAHS, Chengdu, Old Ser. Preliminary study of genus Vibrissaphora (Amphibia: Salientia) and discussion on problems of amphibian classification. Copeia. 1980;3:1-9.
5. Rao DQ and Wilkinson JA. Phylogenetic relationships of the mustache toads inferred from mtDNA sequences. Molecular Phylogenetics & Evolution. 2008;46 1:61-73.
6. Zheng Y, Li S and Fu J. A phylogenetic analysis of the frog genera Vibrissaphora and Leptobrachium, and the correlated evolution of nuptial spine and reversed sexual size dimorphism. Molecular Phylogenetics & Evolution. 2008;46 2:695-707.
7. Zheng Y, Deng D, Li S and Fu J. Aspects of the breeding biology of the Omei mustache toad (*Leptobrachium boringii*): Polygamy and paternal care. Amphibia-Reptilia. 2010;31 2:183-94.
8. Zhang W, Guo Y, Li J, Huang L, Kazitsa EG and Wu H. Transcriptome analysis reveals the genetic basis underlying the seasonal development of keratinized nuptial spines in *Leptobrachium boringii*. Bmc Genomics. 2016;17 1:978.
9. Zheng Y, Rao D, Murphy RW and Zeng X. Reproductive Behavior and Underwater Calls in the Emei Mustache Toad, *Leptobrachium boringii*. Asian Herpetological Research. 2011;02 4:199-215.
10. Hudson CM, Xianjin HE and Jinzhong FU. Keratinized Nuptial Spines Are Used for Male Combat in the Emei Moustache Toad (*Leptobrachium boringii*). Asian Herpetological Research. 2011;02 3:142-8.
11. Genome 10K: a proposal to obtain whole-genome sequence for 10,000 vertebrate species. The Journal of heredity. 2009;100 6:659-74. doi:10.1093/jhered/esp086.
12. Pryszcz LP and Gabaldón T. Redundans: an assembly pipeline for highly heterozygous genomes. Nucleic Acids Research. 2016;44 12:e113-e.
13. Walker BJ, Abeel T, Shea T, Priest M, Abouelliel A, Sakthikumar S, et al. Pilon: an integrated tool for comprehensive microbial variant detection and genome assembly improvement. PLoS One. 2014;9 11:e112963. doi:10.1371/journal.pone.0112963.
14. Servant N, Varoquaux N, Lajoie BR, Viara E, Chen CJ, Vert JP, et al. HiC-Pro: an optimized and flexible pipeline for Hi-C data processing. Genome Biology. 2015;16 1:259.
15. Durand N, Shamim M, Machol I, Rao SP, Huntley M, Lander E, et al. Juicer Provides a One-Click System for Analyzing Loop-Resolution Hi-C Experiments. Cell Systems. 2016;3 1:95-8.
16. Dudchenko O, Batra SS, Omer AD, Nyquist SK, Hoeger M, Durand NC, et al. De novo assembly of the *Aedes aegypti* genome using Hi-C yields chromosome-length scaffolds. Science. 2017;356 6333:92.
17. Wilkinson JA. A New Species of the Genus Vibrissaphora (Anura: Megophryidae) from Yunnan Province, China. Herpetologica. 2006;62 1:90-5.
18. Simão FA, Waterhouse RM, Panagiotis I, Kriventseva EV and Zdobnov EM. BUSCO: assessing genome assembly and annotation completeness with single-copy orthologs. Bioinformatics. 2015;31 19:3210-2.
19. Li H and Durbin R. Fast and accurate short read alignment with Burrows-Wheeler transform. 2009.
20. Chang Z, Li G, Liu J, Zhang Y, Ashby C, Liu D, et al. Bridger: a new framework for

de novo transcriptome assembly using RNA-seq data. Genome Biology. 2015;16 1:30.

21. Pertea G, Huang X, Liang F, Antonescu V, Sultana R, Karamycheva S, et al. TIGR Gene Indices clustering tools (TGICL): a software system for fast clustering of large EST datasets. Bioinformatics. 2003;19 5:651-2.

22. Benson G. Tandem repeats finder: a program to analyze DNA sequences. Nucleic Acids Res. 1999;27 2:573-80.

23. Bedell JA, Korf I, . and Gish W, . MaskerAid: a performance enhancement to RepeatMasker. Bioinformatics. 2000;16 11:1040-1.

24. Stanke M and Waack S. Gene prediction with a hidden Markov model and a new intron submodel. Bioinformatics. 2003;19 suppl_2:215--25.

25. Kerstin H, Clark MD, Torroja CF, James T, Camille B, Matthieu M, et al. The zebrafish reference genome sequence and its relationship to the human genome. Nature. 2013.

26. Yan-Bo S, Zi-Jun X, Xue-Yan X, Shi-Ping L, Wei-Wei Z, Xiao-Long T, et al. Whole-genome sequence of the Tibetan frog *Nanorana parkeri* and the comparative evolution of tetrapod genomes. Proc Natl Acad Sci U S A. 2015;11 112:E1257-E62.

27. Lander ES, Linton LM, Birren B, Nusbaum C, Zody MC, Baldwin J, et al. Initial sequencing and analysis of the human genome. Nature. 2001;409 6822:860-921. doi:10.1038/35057062.

28. Sequence and comparative analysis of the chicken genome provide unique perspectives on vertebrate evolution. Nature. 2004;432 7018:695-716. doi:10.1038/nature03154.

29. Wang Z, Pascual-Anaya J, Zadissa A, Li W, Niimura Y, Huang Z, et al. The draft genomes of soft-shell turtle and green sea turtle yield insights into the development and evolution of the turtle-specific body plan. Nat Genet. 2013;45 6:701-6. doi:10.1038/ng.2615.

30. Session AM, Uno Y, Kwon T, Chapman JA, Toyoda A, Takahashi S, et al. Genome evolution in the allotetraploid frog *Xenopus laevis*. Nature. 2016;538 7625:336-43. doi:10.1038/nature19840.

31. Smith JJ and Timoshevskaya N. The sea lamprey germline genome provides insights into programmed genome rearrangement and vertebrate evolution. 2018;50 2:270-7. doi:10.1038/s41588-017-0036-1.

32. Haas BJ, Salzberg SL, Zhu W, Pertea M, Allen JE, Orvis J, et al. Automated eukaryotic gene structure annotation using EVidenceModeler and the Program to Assemble Spliced Alignments. Genome Biology. 2008;9 1:R7.

33. Jessica AL, Federica DP, Manfred G, Christina W, Lesheng K, Evan M, et al. The genome of the green anole lizard and a comparative analysis with birds and mammals. Nature. 2011.

34. Edwards RJ, Tuipulotu DE, Amos TG, O'Meally D, Richardson MF, Russell TL, et al. Draft genome assembly of the invasive cane toad, *Rhinella marina*. GigaScience. 2018;7 9 doi:10.1093/gigascience/giy095.

34. ~~Nowoshilow S, Schloissnig S, Fei JF, Dahl A, Pang AWC, Pippel M, et al. The axolotl genome and the evolution of key tissue formation regulators. Nature.~~

~~2018;554 7690.~~

35. Hammond SA, Warren RL, Vandervalk BP, Kucuk E, Khan H, Gibb EA, et al. The North American bullfrog draft genome provides insight into hormonal regulation of long noncoding RNA. 2017;8 1:1433. doi:10.1038/s41467-017-01316-7.

36. Smith JJ, Timoshevskaya N, Timoshevskiy VA, Keinath MC, Hardy D and Voss SR. A chromosome-scale assembly of the axolotl genome. Genome Res. 2019;29 2:317-24. doi:10.1101/gr.241901.118.

37. Nowoshilow S, Schloissnig S, Fei JF, Dahl A, Pang AWC, Pippel M, et al. The axolotl genome and the evolution of key tissue formation regulators. Nature. 2018;554 7690.

~~37~~38. Wan QH, Pan SK, Hu L, Zhu Y, Xu PW, Xia JQ, et al. Genome analysis and signature discovery for diving and sensory properties of the endangered Chinese alligator. Cell research. 2013;23 9:1091-105. doi:10.1038/cr.2013.104.

~~38~~39. Li L, Jr SC and Roos DS. OrthoMCL: identification of ortholog groups for eukaryotic genomes. Genome Research. 2003;13 9:2178-89.

~~39~~40. Edgar RC. MUSCLE: multiple sequence alignment with high accuracy and high throughput. Nucleic Acids Res. 2004;32 5:1792-7. doi:10.1093/nar/gkh340.

~~40~~41. Edgar RC. MUSCLE: a multiple sequence alignment method with reduced time and space complexity. Bmc Bioinformatics. 2004.

~~41~~42. Alexandros S. RAxML version 8: a tool for phylogenetic analysis and post-analysis of large phylogenies. Bioinformatics. 2014;30 9:1312-3.

~~42~~43. Yang Z. PAML 4: phylogenetic analysis by maximum likelihood. Molecular biology and evolution. 2007;24 8:1586-91. doi:10.1093/molbev/msm088.

~~43~~44. Tijl DB, Nello C, Demuth JP and Hahn MW. CAFE: a computational tool for the study of gene family evolution. Bioinformatics. 2006;22 10:1269-71.

~~44~~45. Takezaki N, Rzhetsky A, and Nei M. Phylogenetic test of the molecular clock and linearized trees. Molecular Biology & Evolution. 1995;12 5:823-33.

~~45~~46. Green RE, Braun EL, Joel A, Dent E, Ngan N, Glenn H, et al. Three crocodilian genomes reveal ancestral patterns of evolution among archosaurs. Science. 2014;346 6215:1254449.

Tables and Figures

Table 1: Sequencing data used for mustache toad genome assembly and annotation.

Sequencing type	Platform	Library size (bp)	Clean data (Gb)	Application
Genome long reads	PacBio Sequel	20,000	277.15	Contig assembly
Genome short reads	Illumina HiSeq 2500	250	225.03	Genome survey, genome base correction, and genome assessment
Genome Hi-C reads	Illumina HiSeq X-Ten	250	378.78	Chromosome construction
Transcriptome short reads	Illumina HiSeq 4000	250	14.18	Genome annotation and assessment

Table 2: Assembly statistics of the mustache toad genome.

	Wtdbg contig		Hi-C scaffold	
Term	Size (bp)	Number	Size (bp)	Number
N90	153,029	4,866	134,864,763	11
N80	301,658	3,285	181,461,513	8
N70	456,829	2,334	220,042,448	6
N60	624,716	1,671	359,321,214	5
N50	821,125	1,180	412,424,790	4
Max length (bp)	9,978,207		592,710,058	
Total size (bp)	3,530,531,046		3,535,795,546	
Total number (>100bp)	15,899		5,370	

Note: Statistics of genome assembly. Wtdbg contig was the genome assembled by wtdbg and 2-round pilon error-correction. Hi-C scaffold was the genome finished by Hi-C assembly.

Table 3: The BUSCO results of the mustache toad and other amphibian genomes.

Library	*V. ailaonica* (eukaryota)	*V. ailaonica* (metazoa)	*Nanorana parkeri* (eukaryota)	*Xenopus tropicalis* (eukaryota)	*Rhinella marina* (eukaryota)	*Rana catesbeiana* (eukaryota)	*Ambystoma mexicanum* (eukaryota)
%Complete genes	80.8%	85.1%	90.1%	90.1%	90.4%	58.0%	24.4%
%Complete and single-copy genes	78.2%	83.6%	87.8%	88.1%	86.1%	55.4%	23.4%
%Complete and duplicated genes	2.6%	1.5%	2.3%	2.0%	4.3%	2.6%	1.0%
%Fragmented genes	8.9%	4.9%	3.6%	2.0%	3.3%	20.8%	24.4%
%Missing genes	10.3%	10.0%	6.3%	7.9%	6.3%	21.2%	51.2%

Note: Both “eukaryote” and “metazoan” are two core gene sets in BUSCO database.

~~**Table 3: The BUSCO results of the mustache toad genome.**~~

~~Library~~	~~eukaryota~~	~~metazoa~~
~~Complete BUSCOs (C)~~	~~245~~	~~833~~
~~Complete and single-copy BUSCOs (S)~~	~~237~~	~~818~~
~~Complete and duplicated BUSCOs (D)~~	~~8~~	~~15~~
~~Fragmented BUSCOs (F)~~	~~27~~	~~48~~
~~Missing BUSCOs (M)~~	~~31~~	~~97~~
~~Total BUSCO groups searched~~	~~303~~	~~978~~
~~Summarize~~	~~80.8%~~	~~85.1%~~

Formatted: Left
Formatted: Left
Formatted: Left
Formatted: Left
Formatted: Left
Formatted: Left
Formatted: Left
Formatted: Left

Table 4: The quality statistics of several published amphibian genomes.

Species	Contig N50 (bp)	Scaffold N50 (bp)	Genome size (bp)	Genome BUSCO (eukaryota)
Nanorana parkeri	32,798	1,069,101	2,053,867,363	90.1%
Xenopus tropicalis	71,041	135,134,832	1,440,398,454	90.1%
Rhinella marina	166,489	167,498	2,551,759,918	90.4%
Rana catesbeiana	5,415	39,363	6,250,353,185	58.0%
Ambystoma mexicanum	216,366	3,052,786	32,393,605,577	24.4%

Table ~~4~~5: The statistics of *de novo* annotated repeat sequences in mustache toad genome.

Type	Length (bp)	Percentage in genome (%)
DNA	350,793,270	9.94~~3777~~
LINE	297,954,803	8.45~~45989~~
SINE	11,009,363	0.31~~2077~~
LTR	307,317,539	8.71~~1390~~
Other	43,867,330	1.24~~3487~~
Satellite	9,696,790	0.27~~4870~~
Simple repeat	125,397,072	3.55~~4574~~
Unknown	1,114,326,962	31.~~587320~~59
Total	2,147,505,764	60.87~~4369~~

Table ~~5~~6: The functional annotation results of protein-coding genes in mustache toad.

Database	Annotated gene number	Percent (%)
Interpro	12,997	49.56
KEGG	10,035	38.26
SwissProt	12,410	47.32
Trembl	17,916	68.31

Figure 1: The mustache toad, *Vibrissaphora ailaonica*. (A) The adult male individual with spines in the upper jaw. (B) The adult female individual. (C) The adult male individual during the fall off process of spines in the upper jaw. (D) The adult male individual without spines (after fall off process of spines) in the upper jaw. (E) The body size of mustache toad in side view, male (left) and female (right). (F) The body size of mustache toad in top view, male (left) and female (right).

Figure 2: The 17-mer analysis of *Vibrissaphora ailaonica* genome characteristics.

Figure 3: The circos graph showing genome characteristics. From outer circle to inner ring are: gene distribution, tandem repeats (TR), long tandem repeats (LTR), long interspersed nuclear elements (LINE), short interspersed nuclear elements (SINE), and GC content.

Figure 4: The length distributions of annotated protein-coding genes in these species.

Figure 5: The statistics of gene family among these 11 species.

Figure 6: The phylogenetic relationships among these species. The blue numbers represent divergence time. The red dot represents the fossil record used in the node.

Figure 6

Click here to access/download;Figure;Figure 6.pdf

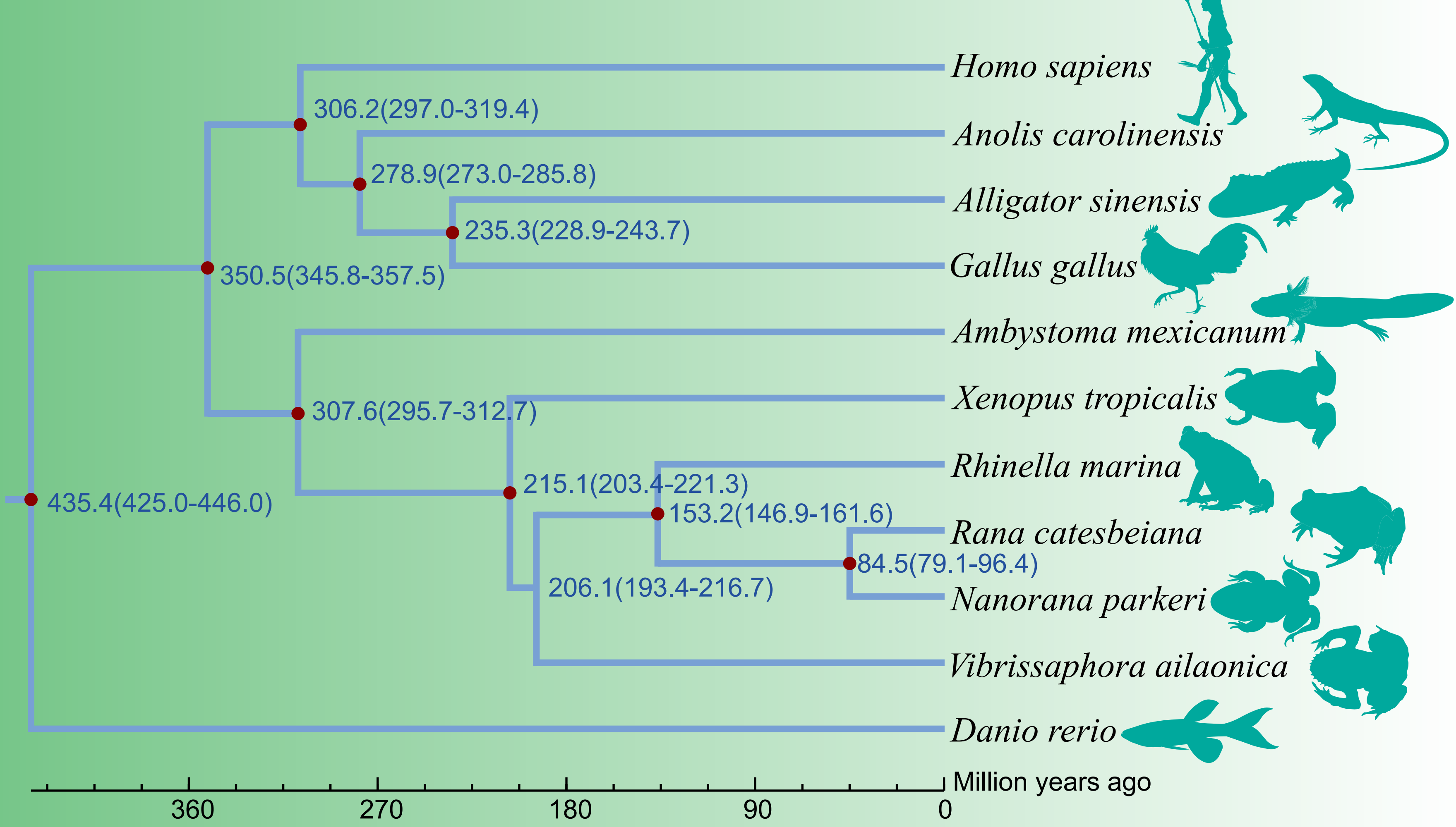

Click here to access/download
Supplementary Material
Supplementary Materials.doc
