# Supplementary material for: Chromosome-level assembly of the mustache toad genome using third-generation DNA sequencing and Hi-C analysis
Source: Gigascience. 2019 Sep 23;8(9):giz114. doi: 10.1093/gigascience/giz114 (PMC6755253; doi:10.1093/gigascience/giz114)
Supplement: giz114_GIGA-D-19-00099_Revision_2 [file giz114_giga-d-19-00099_revision_2.pdf]

GigaScience

Chromosome-level assembly of the mustache toad genome using third-generation DNA sequencing and Hi-C analysis

--Manuscript Draft--

**Manuscript Number:**	GIGA-D-19-00099R2
**Full Title:**	Chromosome-level assembly of the mustache toad genome using third-generation DNA sequencing and Hi-C analysis
**Article Type:**	Data Note
**Funding Information:**	National Key Research and Development Program of China (2017YFC0505202) — Dr. Dingqi Rao<br>National Natural Science Foundation of China (30270175) — Dr. Dingqi Rao<br>National Natural Science Foundation of China (30870278) — Dr. Dingqi Rao<br>National Natural Science Foundation of China (31372165) — Dr. Dingqi Rao

Abstract:

Background

The mustache toad, Vibrissaphora ailaonica, is endemic to China and belongs to the Megophryidae family. Like other mustache toad species, V. ailaonica males temporarily develop keratinized nuptial spines on their upper jaw during each breeding season, which fall off at the end of the breeding season. This feature is likely to be result of the reversal of sexual dimorphism in body size, with males being larger than females. A high-quality reference genome for the mustache toad would be invaluable to investigate the genetic mechanism underlying these repeatedly developing keratinized spines.

Findings

To construct the mustache toad genome, we generated 225 Gb of short reads and 277 Gb of long reads using Illumina and Pacific Biosciences (PacBio) sequencing technologies, respectively. Sequencing data were assembled into a 3.53-Gb genome assembly, with a contig N50 length of 821 Kb. We also used high-throughput chromosome conformation capture (Hi-C) technology to identify contacts between contigs, then assembled contigs into scaffolds and assembled a genome with 13 chromosomes and a scaffold N50 length of 412.42 Mb. Based on the 26,227 protein-coding genes annotated in the genome, we analyzed phylogenetic relationships between the mustache toad and other chordate species. The mustache toad has a relatively higher evolutionary rate and separated from a common ancestor of the marine toad, bullfrog, and Tibetan frog 206.1 million years ago. Furthermore, we identified 201 expanded gene families in the mustache toad, which were mainly enriched in immune pathway, keratin filament, and metabolic processes.

Conclusions

Using Illumina, PacBio, and Hi-C technologies, we constructed the first high-quality chromosome-level mustache toad genome. This work not only offers a valuable reference genome for functional studies of mustache toad traits, but also provides important chromosomal information for wider genome comparisons.

**Corresponding Author:**	Dingqi Rao, Ph.D<br>Kunming Institute of Zoology Chinese Academy of Sciences<br>Kunming, Yunnan CHINA
**Corresponding Author Secondary Information:**	
**Corresponding Author's Institution:**	Kunming Institute of Zoology Chinese Academy of Sciences
**Corresponding Author's Secondary**	

Powered by Editorial Manager® and ProduXion Manager® from Aries Systems Corporation

**Institution:**	
**First Author:**	Yongxin Li
**First Author Secondary Information:**	
**Order of Authors:**	Yongxin Li
	Yandong Ren
	Dongru Zhang
	Hui Jiang
	Zhongkai Wang
	Xueyan Li
	Dingqi Rao, Ph.D
**Order of Authors Secondary Information:**	
**Response to Reviewers:**	All the comments in this version have been corrected.
**Additional Information:**	
**Question**	**Response**
Are you submitting this manuscript to a special series or article collection?	No
**Experimental design and statistics**<br><br>Full details of the experimental design and statistical methods used should be given in the Methods section, as detailed in our Minimum Standards Reporting Checklist. Information essential to interpreting the data presented should be made available in the figure legends.<br><br>Have you included all the information requested in your manuscript?	Yes
**Resources**<br><br>A description of all resources used, including antibodies, cell lines, animals and software tools, with enough information to allow them to be uniquely identified, should be included in the Methods section. Authors are strongly encouraged to cite Research Resource Identifiers (RRIDs) for antibodies, model organisms and tools, where possible.<br><br>Have you included the information requested as detailed in our Minimum	Yes

Standards Reporting Checklist?	
**Availability of data and materials**<br><br>All datasets and code on which the conclusions of the paper rely must be either included in your submission or deposited in publicly available repositories (where available and ethically appropriate), referencing such data using a unique identifier in the references and in the "Availability of Data and Materials" section of your manuscript.<br><br>Have you have met the above requirement as detailed in our Minimum Standards Reporting Checklist?	Yes

Powered by Editorial Manager® and ProduXion Manager® from Aries Systems Corporation

Manuscript
Click here to access/download;Manuscript;Manuscript.doc
Click here to view linked References

Chromosome-level assembly of the mustache toad genome using third-generation DNA sequencing and Hi-C analysis

Running title: The mustache toad genome

Yongxin Li[1,2,‡], Yandong Ren[2,‡], Dongru Zhang[1,‡], Hui Jiang[3], Zhongkai Wang[2], Xueyan Li[1], Dingqi Rao[1,*]

[1] State Key Laboratory of Genetic Resources and Evolution, Kunming Institute of Zoology, Chinese Academy of Sciences, Kunming 650223, China; [2] Center for Ecological and Environmental Sciences, Northwestern Polytechnical University, Xi'an 710072, China; [3] National Engineering Laboratory of Marine Germplasm Resources Exploration and Utilization, Zhejiang Ocean University, Zhoushan 316022, China

[‡]Equal contribution

[*]Correspondence address. Dingqi Rao. State Key Laboratory of Genetic Resources and Evolution, Kunming Institute of Zoology, Chinese Academy of Sciences, Kunming 650223, China. Tel: +86-0871-65128516; E-mail: kizar@mail.kiz.ac.cn; ORCID: 0000-0003-2680-1503

Abstract

Background: The mustache toad, *Vibrissaphora ailaonica*, is endemic to China and belongs to the Megophryidae family. Like other mustache toad species, *V. ailaonica*

males temporarily develop keratinized nuptial spines on their upper jaw during each breeding season, which fall off at the end of the breeding season. This feature is likely to be result of the reversal of sexual dimorphism in body size, with males being larger than females. A high-quality reference genome for the mustache toad would be invaluable to investigate the genetic mechanism underlying these repeatedly developing keratinized spines. **Findings:** To construct the mustache toad genome, we generated 225 Gb of short reads and 277 Gb of long reads using Illumina and Pacific Biosciences (PacBio) sequencing technologies, respectively. Sequencing data were assembled into a 3.53-Gb genome assembly, with a contig N50 length of 821 Kb. We also used high-throughput chromosome conformation capture (Hi-C) technology to identify contacts between contigs, then assembled contigs into scaffolds and assembled a genome with 13 chromosomes and a scaffold N50 length of 412.42 Mb. Based on the 26,227 protein-coding genes annotated in the genome, we analyzed phylogenetic relationships between the mustache toad and other chordate species. The mustache toad has a relatively higher evolutionary rate and separated from a common ancestor of the marine toad, bullfrog, and Tibetan frog 206.1 million years ago. Furthermore, we identified 201 expanded gene families in the mustache toad, which were mainly enriched in immune pathway, keratin filament, and metabolic processes. **Conclusions:** Using Illumina, PacBio, and Hi-C technologies, we constructed the first high-quality chromosome-level mustache toad genome. This work not only offers a valuable reference genome for functional studies of mustache toad traits, but also provides important chromosomal information for wider genome comparisons.

Keywords: Mustache toad; Genome assembly; Evolution; PacBio; Hi-C

Data Description

The mustache toad, *Vibrissaphora ailaonica* (NCBI: txid:428466), is an amphibian belonging to the Megophryidae family that is endemic to China (including the China–Vietnam border) [1–3]. This mustache toad species exhibits many interesting features, including unique keratinized spines along the upper jaw [1, 4–6]. These spines grow repeatedly in sexually mature males during the breeding season, and fall off at the end of this process [5–8] (Figure 1). This morphological difference between males and females is further highlighted by their sexual dimorphism in body size (males are significantly larger than females). The spines (and body size) may be used as a weapon for sexually mature individuals to compete for nests and mating opportunities [7, 9, 10]. Another unique aspect of the mustache toad is that breeding occurs during the cold season, whereas most frogs and toads breed in the warmer months [1]. However, despite the importance of the mustache toad in terms of dynamic spine development and sexual dimorphism in body size, few genomic resources exist for this species. In fact, to date, no next-generation sequencing (NGS) data have been reported in the *Vibrissaphora* genus. The lack of genome sequence and transcriptome data for *V. ailaonica* has hindered identification of functional genes related to their attractive and dynamic appearance (e.g., spine and body size). The shortage of amphibian genomes represented in the Genome 10K project makes it

necessary to analyze other important genomes to study phylogenetic relationships in amphibians on a larger scale [11].

In this study, we combined genomic sequencing data from Illumina short reads, PacBio long reads, and Hi-C data, to generate the first chromosome-level reference genome for the mustache toad. The completeness and continuity of the genome were comparable with that of other important amphibian species. The high-quality reference genome generated in this study will facilitate research on population genetic traits and functional gene identification related to important characteristics of the mustache toad.

Analyses and Methods

Sampling and sequencing

During the breeding season (in February), a male mustache toad (*V. ailaonica*) with keratinized nuptial spines on its upper jaw was caught for sequencing from Ailao Mountain (Figure 1). To obtain sufficient high-quality DNA for the PacBio Sequel platform (Pacific Biosciences, USA), the mustache toad was dissected, and fresh liver tissue was used for DNA extraction using phenol/chloroform extraction. DNA quality was checked by agarose gel electrophoresis, and high integrity DNA molecules were obtained. Other tissues, including spines, brain, stomach, intestine, liver, lung, spleen, blood, and tongue, were snap-frozen in liquid nitrogen for 10 min. These 9 organs/tissues were stored at −80°C for RNA-seq analysis. Isolated total RNA was used to isolate intact poly (A) + RNA using the NEBnext Ultra-Directional RNA

Library Prep kit (NEB, protocol B) for library construction. The mRNA was further fragmented and randomly primed during first-strand synthesis by reverse transcription. This procedure was followed by second-strand synthesis with DNA polymerase I to create double-stranded cDNA fragments using Transcriptor First Strand cDNA Synthesis Kit (Roche).

For the Hi-C experiments, collected blood was used for library construction. The blood sample (150 µl) was cross-linked for 10 min with formaldehyde (1% final concentration), after which glycine (0.2 M final concentration) was added for 5 min to stop the cross-linking process. The sample was then stored until required for further analysis.

Extracted DNA was sequenced using the Illumina and PacBio Sequel platforms. Short reads generated from the Illumina platform were used to estimate genome size and to correct errors in the assembled genome, and the PacBio long reads were used for genome assembly. To this end, five libraries with insertion lengths of 220 bp or 500 bp were sequenced on an Illumina HiSeq 2500 platform, generating 150-bp paired-end reads. A 20-Kb library was constructed using the PacBio platform, according to the manufacturer’s protocols. Finally, we obtained 225.03 Gb of Illumina short reads and 277.15 Gb of PacBio long reads (Table 1, Additional Table S1, Additional Table S2). The average N50 length of subreads was 14.78 Kb, providing ultra-long genomic sequences for the following assembly and analysis (Additional Table S2).

RNA-seq samples were obtained by mixing an equal amount of RNA extracted from

each tissue that had been stored and used for library construction. After sequencing on the Illumina HiSeq 4000 platform, we obtained 14.18 Gb of sequencing data (Table 1, Additional Table S3). Four Hi-C libraries were constructed using the same sample with same parameters, and sequenced on the Illumina Hiseq X-ten platform, which generated 378.78 Gb of clean data (Table 1, Additional Table S4).

Genome characteristics estimation

Illumina short reads were filtered for quality as follows. First, adaptors were removed from the sequencing reads. Then, read pairs were excluded if any one read had more than 10% ‘N’, and read pairs with more than 50% low quality bases were removed. Finally, PCR duplicates produced during library construction were removed.

Filtered reads were used to estimate genome size and other characteristics. Using the *k*-mer method, we calculated the 17-mer depth frequency distribution in the mustache toad. Genome size was estimated by:

$$G = TKN_{17\text{-mer}} / PKFD_{17\text{-mer}}$$

where $TKN_{17\text{-mer}}$ is the total *k*-mer number, and $PKFD_{17\text{-mer}}$ is the peak *k*-mer frequency depth of 17-mer.

We estimated a genome size of 3.52 Gb (peak = 54) and found heterozygous, repeated sequence peaks, suggesting that the mustache toad genome exhibits complex genome assembly (Figure 2).

Genome assembly using PacBio long reads and Hi-C data

Based on 38 single-molecule real-time cells, and using the PacBio Sequel platform, we generated 277.15 Gb of subreads (Table 1, Additional Table S2). The average and N50 length of subreads was 9.65 Kb and 14 78 Kb, respectively (Additional Table S2). All long reads were assembled using wtdbg software [12] (WTDBG, RRID: SCR_017225). As a result, we obtained a 3.95-Gb genome assembly, with a contig N50 length of 739.54 Kb. However, although the size of the genome assembly was comparable with the estimated *k*-mer result, the end result was a slightly larger. This may be associated with the complexity of the mustache toad genome (which has a high rate of heterozygosity rate and repetitive sequences). Redundancy in the genome assembly was removed using Redundans software (v0.13c) [13], with an identity of 0.7 and overlap of 0.7. This resulted in a genome assembly of 3.58 Gb and a contig N50 length of 834.90 Kb. To ensure that all contigs removed were not real sequences, we used BUSCO (Benchmarking Universal Single-Copy Orthologs) [14] and the mapping ratio of Illumina reads in both the raw genome and the redundancy-filtered genome. Results of these checks indicated that the parameters used in the redundancy-filtered step were appropriate for this study (Additional Table S5). To further improve the quality and accuracy of our genome assembly, Illumina short reads were used to polish the genome using Pilon software (Pilon, RRID: SCR_014731, v1.21) [15] at the single-base level.

Hi-C data were used to improve the connection integrity of the contigs (15,899 contigs). We obtained 378.78 Gb of Hi-C sequencing data, which was first filtered using Hic-Pro (v2.10.0) [16] (Table 1, Additional Table S4), and then mapped to the

polished mustache toad genome [17]. The locations and directions of the contigs were determined by 3D *de novo* assembly (3d-DNA) software (v180419) [18], with default parameters. Most contigs were then successfully clustered and anchored in 13 groups (Figure 3) [19]. Finally, we obtained the first chromosome-level, high quality mustache toad assembly (3.53 Gb) with a scaffold N50 length of 412.42 Mb, which provides a solid genomic resource to assist further study of the mustache toad (Table 2).

Genome assembly evaluation

The quality of a genome assembly is directly related to the accuracy and completeness of protein-coding gene prediction. Therefore, we evaluated the assembled mustache toad genome using three methods. First, the assembled genome was compared against the core gene set in BUSCO (BUSCO, RRID:SCR_015008, v2.0) [14]. We found 245 (80.8%) and 833 (85.1%) conserved core genes in the mustache toad genome using the eukaryote and metazoan databases, respectively (Table 3). When we further considered the fragmented BUSCO genes found in the genome, there were 272 (89.7%) and 881 (90.1%) conserved core genes in the eukaryote and metazoan databases, respectively. These results indicated that the assembled mustache toad genome is comparable with published amphibian genomes (Table 3).

Second, all filtered short reads generated from the Illumina platform were aligned to the genome using BWA (Burrows–Wheeler Aligner) software (BWA, RRID:SCR_010910, v0.7.12) [20]; 1,778 million clean reads could be mapped to the

genome, accounting for 97.78% of total clean reads (Additional Table S6). Third, RNA-seq reads were *de novo*-assembled using Bridger software (Bridger, RRID:SCR_017039, version: r2014-12-01) [21], with redundant transcripts removed by TGICL [22]. This resulted in 19,876 transcripts (Additional Table S7). These transcripts were then aligned to the genome, with 17,878 transcripts (89.95%) found in the assembled genome, and 94.52% of transcripts being longer than 1 Kb (Additional Table S8). Analysis of N50 length and BUSCO results revealed that the mustache toad genome was comparable with that of other published amphibian genomes (Tables 2–4), indicating that our assembled mustache toad genome exhibited high completeness and accuracy.

The GC distribution of the mustache toad genome, and that of other vertebrate species, was calculated using the slide window method. GC distributions were similar, with an average GC content of 43.68% in the mustache toad, and 36.60% to 44.49% in other species (Additional Figure S1).

Genome annotation

Tandem Repeat Finder (TRF, v4.04) [23] was used to identify repetitive elements, and RepeatModeler software (RepeatModeler, RRID:SCR_015027, v1.0.4) was used to detect transposable elements (TEs) in the mustache toad genome. Then, the *de novo* library of repeats produced by RepeatModeler analysis and the repbase (RepBase16.02) database were used for RepeatMasker (RepeatMasker, RRID:SCR_012954, version: open-4.0) [24] analysis to identify homologous repeats.

RepeatProteinMask was used to query the TE protein database at the protein level. Lastly, we identified 2.45 Gb of repeat sequences, accounting for 69.48% of the estimated genome size (Additional Table S9). Among these repeat sequences, 60.87% (2.15 Gb) was predicted by the *de novo* method (Table 5).

After repeat sequence annotation, we masked all repeats, except for the tandem repeat sequences, for protein-coding gene annotation. Augustus software (Augustus, RRID:SCR_008417, v2.5.5) [25] was used to *de novo*-predict coding genes using a zebrafish (*Danio rerio*) dataset as the training species. For the homology-based method, protein sequences of chordate species, including *D. rerio* (GCF_000002035.6) [26], *Nanorana parkeri* (GCF_000935625.1) [27], *Homo sapiens* (GCF_000001405.38) [28], *Gallus gallus* (GCF_000002315.5) [29], *Pelodiscus sinensis* (GCF_000230535.1) [30], *Xenopus laevis* (GCF_001663975.1) [31], and *Petromyzon marinus* [32], were downloaded and aligned against the mustache toad genome using the TBLASTN module (TBLASTN, RRID:SCR_011822, BLAST version: 2.3.0). The transcripts assembled by RNA-seq reads were first translated into amino acids and then aligned to the genome using TBLASTN software for gene annotation. EVidenceModeler (EVidenceModeler, RRID:SCR_014659, version: r2012-06-25) [33] was used to integrate results from the three methods, and genes with poor transcriptome evidence support were filtered out. Finally, 26,227 high-quality protein-coding genes were predicted in the mustache toad genome. The distributions of mRNA, coding sequences, exon and intron lengths were comparable with those of closely related species (Figure 4).

Gene functional annotation can help to elucidate gene function. Thus, we aligned all 26,227 protein-coding genes to protein databases, including InterProScan, Kyoto Encyclopedia of Genes and Genomes (KEGG), SwissProt, and TrEMBL. Results showed that most of the genes obtained could be annotated from these functional databases (Table 6).

Phylogenetic tree and divergence time analysis

To reveal phylogenetic relationships between the mustache toad and other closely related species, we identified the single-copy genes among these species. First, protein sequences, including those of *D. rerio* (GCF_000002035.6) [26], *N. parkeri* (GCF_000935625.1) [27], *H. sapiens* (GCF_000001405.38) [28], *G. gallus* (GCF_000002315.5) [29], *Anolis carolinensis* (GCF_000090745.1) [34], *Xenopus tropicalis* (GCF_000004195.3) [31], *Rhinella marina* (GigaDB) [35], *Rana catesbeiana* (GCA_002284835.2) [36], *Ambystoma mexicanum* [37, 38], and *Alligator sinensis* (GCF_000455745.1) [39], were downloaded from the National Center for Biotechnology Information (NCBI). The longest transcript of each gene in each species was selected. BLASTP (BLASTP, RRID:SCR_001010, BLAST version: 2.2.24) was then used to align these protein sequences from the 11 species (including the mustache toad), with an e-value of 1e-5. Homology relationships (including orthologs and paralogs) were then determined using OrthoMCL software (v1.4) [40]. Genes with only one copy in the species were identified as single-copy genes. In total, 238 genes were identified (Figure 5). Detailed statistics about gene families are shown

in Additional Table S10.

The 238 single-copy genes were aligned using MUSCLE software (MUSCLE, RRID:SCR_011812, v3.8.31) [41, 42], and concatenated to supergenes for maximum-likelihood-based phylogenetic analyses. We performed phylogenetic analysis, with zebrafish as the outgroup, using RAxML software (RAxML, RRID:SCR_006086, v8.2.3) [43], with the parameter '-m' for PROTGAMMAAUTO. Results indicated that the mustache toad has a close relationship with the ancestor of the marine toad (*R. marina*), bullfrog (*R. catesbeiana*), and Tibetan frog (*N. parkeri*), with topological relationships in other clades found to be the same as reported previously (Figure 6). To further investigate the divergence time of these species, especially toad and frogs, the MCMCTREE model (part of PAML software package; PAML, RRID:SCR_014932, v4.8) [44] was used with three datasets (four-fold degenerate sites [4dTVs], first-codon sites, and second-codon sites) extracted from the single-copy genes as the input file. Fossil records were downloaded from the TIMETREE website [45] and used to calibrate the results. Results from the three different datasets were very similar, showing that the mustache toad diverged from the common ancestor of the marine toad, bullfrog and Tibetan frog about 206.1 million years ago (Figure 6; Additional Figure S2, Additional Figure S3).

Gene family expansion and contraction

We performed gene family expansion and contraction analysis using CAFÉ software (CAFÉ, RRID:SCR_005983, v4.0) [46], and found 201 and 326 expanded and

contracted gene families in the mustache toad (*P* < 0.05), respectively. Using the Gene Ontology (GO) and KEGG databases, functional enrichment analysis of expanded gene families revealed 210 GO terms (adjusted *P* < 0.05) and 9 KEGG pathways (*q* < 0.05) to be significantly enriched (Additional Table S11, Additional Table S12). The expanded gene families were mainly related to metabolic processes, intermediate filament terms, enzyme activities, and immune terms. For example, cellular metabolic process (adjusted *P* = 6.06E-14), intermediate filament (adjusted *P* = 3.42E-15), keratin filament (adjusted *P* = 2.94E-13), endoribonuclease activity (adjusted *P* = 9.19E-08), and immune response (*q* = 8.36E-03) were enriched (Additional Table S11, Additional Table S12). In addition, for the contracted gene families, 220 GO terms (adjusted *P* < 0.05) and 9 KEGG pathways (*q* < 0.05) were enriched, respectively (Additional Table S13, Additional Table S14). These enriched terms were mainly involved in ion binding and transporter activity, including neurotransmitter transporter activity (adjusted *P* = 1.89E-11), sodium ion transmembrane transporter activity (adjusted *P* = 3.33E-06), and secondary active transmembrane transporter activity (adjusted *P* = 1.86E-08) (Additional Table S13, Additional Table S14). Thus, these biological processes may be related to the special characteristics of the mustache toad.

Relative evolutionary rate of species

The evolutionary rate of species can reflect its evolutionary history and status. The relative evolutionary rate of the mustache toad to other closely related species was

analyzed using LINTRE [47] and MEGA (MEGA, RRID:SCR_000667, v7.0.26) software. Two-cluster analysis was applied to test the molecular evolution of multiple sequences in a phylogenetic context, based on concatenated supergenes (protein sequences) using *tpcv* (a module in LINTRE software). Concatenated supergenes were also used for Tajima's relative rate test. We used zebrafish as the outgroup in both methods, and found that, except for the axolotl, the mustache toad had a relatively faster evolutionary rate than its closely related species (e.g., *X. tropicalis*, *R. marina*, *R. catesbeiana*, and *N. parkeri*) (Additional Table S15, Additional Table S16). The crocodile had a slower evolutionary rate, relative to its closely related species, which is consistent with previous work [48] (Additional Table S15, Additional Table S16).

Discussion

Using Illumina, PacBio, and Hi-C sequencing technologies, we report the first chromosome-level genome assembly of the mustache toad. We successfully annotated the high-quality protein-coding genes by integrating results from three different methods. Phylogenetic analysis indicated that the mustache toad is closely related to the marine toad, bullfrog, and Tibetan frog. Analysis showed that the mustache toad had a faster evolutionary rate, relative to most other closely related species studied. Analysis of the expansion and contraction of gene families identified several biological processes and pathways, such as metabolism and intermediate filaments, suggesting that these terms may relate to the special adaptations of the mustache toad

to its habitat. This work not only offers a valuable chromosome-level genomic data for comparative genomics analysis, but also provides important genomic data for studying the mustache toad traits.

Availability of supporting data and materials

Raw sequencing data were deposited in the NCBI database under accession number PRJNA523649. Genome assembly and annotation results are available via the *GigaScience* repository, GigaDB [49].

Declarations

List of Abbreviations

BLAST: Basic Local Alignment Search Tool; BUSCO: Benchmarking Universal Single-Copy Orthologs; GO: Gene Ontology; Hi-C: High-throughput chromosome conformation capture; KEGG: Kyoto Encyclopedia of Genes and Genomes.

Competing interests

The authors declare that they have no competing interests.

Funding

This work was supported by the National Key Research and Development Program of China (grant number 2017YFC0505202) and the National Natural Science Foundation of China (grant numbers NSFC-30270175, NSFC-30870278, and

NSFC-31372165).

Authors' contributions

D.R. designed the project; D.R. and D.Z. collected the samples; Y.L. and Y.R. estimated the genome size and assembled the genome; Y.L. polished the assembled genome and analyzed Hi-C data; H.J. performed the genome annotation; Y.L. and Z.W. assessed the quality of the genome assembly; Y.L. and Y.R. constructed the phylogenetic tree and determined divergence time, relative evolutionary rate of species, and expansion and contraction of gene families. Y.L., D.R., Y.R., and X.L. wrote the manuscript. All authors read and approved the final version of the manuscript.

Acknowledgements

Not applicable.

References

1. Liang F and Changyuan Y. Amphibians of China. Science Press: Beijing; 2016. ISBN-10: 9401795630
2. Matsui M, Hamidy A, Murphy RW, Khonsue W, Yambun P, Shimada T, et al. Phylogenetic relationships of megophryid frogs of the genus Leptobrachium (Amphibia, Anura) as revealed by mtDNA gene sequences. Molecular Phylogenetics & Evolution. 2010;56 1:259-72.

3. Matsui M. A New Leptobrachium (Vibrissaphora) from Laos (Anura: Megophryidae). Current Herpetology. 2013;32 2:182-9.

4. Liu C HS, Zhao EJAHS, Chengdu, Old Ser. Preliminary study of genus Vibrissaphora (Amphibia: Salientia) and discussion on problems of amphibian classification. Copeia. 1980;3:1-9.

5. Rao DQ and Wilkinson JA. Phylogenetic relationships of the mustache toads inferred from mtDNA sequences. Molecular Phylogenetics & Evolution. 2008;46 1:61-73.

6. Zheng Y, Li S and Fu J. A phylogenetic analysis of the frog genera Vibrissaphora and Leptobrachium, and the correlated evolution of nuptial spine and reversed sexual size dimorphism. Molecular Phylogenetics & Evolution. 2008;46 2:695-707.

7. Zheng Y, Deng D, Li S and Fu J. Aspects of the breeding biology of the Omei mustache toad (*Leptobrachium boringii*): Polygamy and paternal care. Amphibia-Reptilia. 2010;31 2:183-94.

8. Zhang W, Guo Y, Li J, Huang L, Kazitsa EG and Wu H. Transcriptome analysis reveals the genetic basis underlying the seasonal development of keratinized nuptial spines in *Leptobrachium boringii*. Bmc Genomics. 2016;17 1:978.

9. Zheng Y, Rao D, Murphy RW and Zeng X. Reproductive Behavior and Underwater Calls in the Emei Mustache Toad, *Leptobrachium boringii*. Asian Herpetological Research. 2011;02 4:199-215.

10. Hudson CM, Xianjin HE and Jinzhong FU. Keratinized Nuptial Spines Are Used for Male Combat in the Emei Moustache Toad (*Leptobrachium boringii*). Asian Herpetological Research. 2011;02 3:142-8.

11. Genome 10K: a proposal to obtain whole-genome sequence for 10,000 vertebrate species. The Journal of heredity. 2009;100 6:659-74. doi:10.1093/jhered/esp086.

12. WTDBG. https://github.com/ruanjue/wtdbg-1.2.8.

13. Pryszcz LP and Gabaldón T. Redundans: an assembly pipeline for highly heterozygous genomes. Nucleic Acids Research. 2016;44 12:e113-e.

14. Simão FA, Waterhouse RM, Panagiotis I, Kriventseva EV and Zdobnov EM. BUSCO: assessing genome assembly and annotation completeness with single-copy orthologs. Bioinformatics. 2015;31 19:3210-2.

15. Walker BJ, Abeel T, Shea T, Priest M, Abouelliel A, Sakthikumar S, et al. Pilon: an integrated tool for comprehensive microbial variant detection and genome assembly improvement. PLoS One. 2014;9 11:e112963. doi:10.1371/journal.pone.0112963.

16. Servant N, Varoquaux N, Lajoie BR, Viara E, Chen CJ, Vert JP, et al. HiC-Pro: an optimized and flexible pipeline for Hi-C data processing. Genome Biology. 2015;16 1:259.

17. Durand N, Shamim M, Machol I, Rao SP, Huntley M, Lander E, et al. Juicer Provides a One-Click System for Analyzing Loop-Resolution Hi-C Experiments. Cell Systems. 2016;3 1:95-8.

18. Dudchenko O, Batra SS, Omer AD, Nyquist SK, Hoeger M, Durand NC, et al. De novo assembly of the *Aedes aegypti* genome using Hi-C yields chromosome-length scaffolds. Science. 2017;356 6333:92.

19. Wilkinson JA. A New Species of the Genus Vibrissaphora (Anura: Megophryidae) from Yunnan Province, China. Herpetologica. 2006;62 1:90-5.

20. Li H and Durbin R. Fast and accurate short read alignment with Burrows-Wheeler transform. 2009.

21. Chang Z, Li G, Liu J, Zhang Y, Ashby C, Liu D, et al. Bridger: a new framework for de novo transcriptome assembly using RNA-seq data. Genome Biology. 2015;16 1:30.

22. Pertea G, Huang X, Liang F, Antonescu V, Sultana R, Karamycheva S, et al. TIGR Gene Indices clustering tools (TGICL): a software system for fast clustering of large EST datasets. Bioinformatics. 2003;19 5:651-2.

23. Benson G. Tandem repeats finder: a program to analyze DNA sequences. Nucleic Acids Res. 1999;27 2:573-80.

24. Bedell JA, Korf I, . and Gish W, . MaskerAid: a performance enhancement to RepeatMasker. Bioinformatics. 2000;16 11:1040-1.

25. Stanke M and Waack S. Gene prediction with a hidden Markov model and a new intron submodel. Bioinformatics. 2003;19 suppl_2:215--25.

26. Kerstin H, Clark MD, Torroja CF, James T, Camille B, Matthieu M, et al. The zebrafish reference genome sequence and its relationship to the human genome. Nature. 2013. 496(7446):498-503. doi: 10.1038/nature12111.

27. Yan-Bo S, Zi-Jun X, Xue-Yan X, Shi-Ping L, Wei-Wei Z, Xiao-Long T, et al. Whole-genome sequence of the Tibetan frog *Nanorana parkeri* and the comparative evolution of tetrapod genomes. Proc Natl Acad Sci U S A. 2015;11 112:E1257-E62.

28. Lander ES, Linton LM, Birren B, Nusbaum C, Zody MC, Baldwin J, et al. Initial sequencing and analysis of the human genome. Nature. 2001;409 6822:860-921. doi:10.1038/35057062.

29. Sequence and comparative analysis of the chicken genome provide unique perspectives on vertebrate evolution. Nature. 2004;432 7018:695-716. doi:10.1038/nature03154.

30. Wang Z, Pascual-Anaya J, Zadissa A, Li W, Niimura Y, Huang Z, et al. The draft genomes of soft-shell turtle and green sea turtle yield insights into the development and evolution of the turtle-specific body plan. Nat Genet. 2013;45 6:701-6. doi:10.1038/ng.2615.

31. Session AM, Uno Y, Kwon T, Chapman JA, Toyoda A, Takahashi S, et al. Genome evolution in the allotetraploid frog *Xenopus laevis*. Nature. 2016;538 7625:336-43. doi:10.1038/nature19840.

32. Smith JJ and Timoshevskaya N. The sea lamprey germline genome provides insights into programmed genome rearrangement and vertebrate evolution. 2018;50 2:270-7. doi:10.1038/s41588-017-0036-1.

33. Haas BJ, Salzberg SL, Zhu W, Pertea M, Allen JE, Orvis J, et al. Automated eukaryotic gene structure annotation using EVidenceModeler and the Program

to Assemble Spliced Alignments. Genome Biology. 2008;9 1:R7.

34. Jessica AL, Federica DP, Manfred G, Christina W, Lesheng K, Evan M, et al. The genome of the green anole lizard and a comparative analysis with birds and mammals. Nature. 2011.

35. Edwards RJ, Tuipulotu DE, Amos TG, O'Meally D, Richardson MF, Russell TL, et al. Draft genome assembly of the invasive cane toad, *Rhinella marina*. GigaScience. 2018;7 9 doi:10.1093/gigascience/giy095.

36. Hammond SA, Warren RL, Vandervalk BP, Kucuk E, Khan H, Gibb EA, et al. The North American bullfrog draft genome provides insight into hormonal regulation of long noncoding RNA. 2017;8 1:1433. doi:10.1038/s41467-017-01316-7.

37. Smith JJ, Timoshevskaya N, Timoshevskiy VA, Keinath MC, Hardy D and Voss SR. A chromosome-scale assembly of the axolotl genome. Genome Res. 2019;29 2:317-24. doi:10.1101/gr.241901.118.

38. Nowoshilow S, Schloissnig S, Fei JF, Dahl A, Pang AWC, Pippel M, et al. The axolotl genome and the evolution of key tissue formation regulators. Nature. 2018;554 7690.

39. Wan QH, Pan SK, Hu L, Zhu Y, Xu PW, Xia JQ, et al. Genome analysis and signature discovery for diving and sensory properties of the endangered Chinese alligator. Cell research. 2013;23 9:1091-105. doi:10.1038/cr.2013.104.

40. Li L, Jr SC and Roos DS. OrthoMCL: identification of ortholog groups for

eukaryotic genomes. Genome Research. 2003;13 9:2178-89.

41. Edgar RC. MUSCLE: multiple sequence alignment with high accuracy and high throughput. Nucleic Acids Res. 2004;32 5:1792-7. doi:10.1093/nar/gkh340.

42. Edgar RC. MUSCLE: a multiple sequence alignment method with reduced time and space complexity. BMC Bioinformatics. 2004; 5 (1) DOI:10.1186/1471-2105-5-113

43. Alexandros S. RAxML version 8: a tool for phylogenetic analysis and post-analysis of large phylogenies. Bioinformatics. 2014;30 9:1312-3.

44. Yang Z. PAML 4: phylogenetic analysis by maximum likelihood. Molecular biology and evolution. 2007;24 8:1586-91. doi:10.1093/molbev/msm088.

45. TIMETREE website. www.timetree.org.

46. Tijl DB, Nello C, Demuth JP and Hahn MW. CAFE: a computational tool for the study of gene family evolution. Bioinformatics. 2006;22 10:1269-71.

47. Takezaki N, Rzhetsky A, and Nei M. Phylogenetic test of the molecular clock and linearized trees. Molecular Biology & Evolution. 1995;12 5:823-33.

48. Green RE, Braun EL, Joel A, Dent E, Ngan N, Glenn H, et al. Three crocodilian genomes reveal ancestral patterns of evolution among archosaurs. Science. 2014;346 6215:1254449.

49. Li Y; Ren Y; Zhang D; Jiang H; Wang Z; Li X; Rao D (2019): Supporting data for "Chromosomal-level assembly of the mustache toad genome using third-generation DNA sequencing and Hi-C analysis" GigaScience Database.

http://dx.doi.org/10.5524/100624

Tables

Table 1: Sequencing data used for mustache toad genome assembly and annotation

Sequencing type	Platform	Library size (bp)	Clean data (Gb)	Application
Genome long reads	PacBio Sequel	20,000	277.15	Contig assembly
Genome short reads	Illumina HiSeq 2500	250	225.03	Genome survey, genome base correction, and genome assessment
Genome Hi-C reads	Illumina HiSeq X-Ten	250	378.78	Chromosome construction
Transcriptome short reads	Illumina HiSeq 4000	250	14.18	Genome annotation and assessment

Table 2: Assembly data for the mustache toad genome

Term	Wtdbg contig		Hi-C scaffold	
	Size (bp)	Number	Size (bp)	Number
N90	153,029	4,866	134,864,763	11

N80	301,658	3,285	181,461,513	8
N70	456,829	2,334	220,042,448	6
N60	624,716	1,671	359,321,214	5
N50	821,125	1,180	412,424,790	4
Max length (bp)	9,978,207		592,710,058	
Total size (bp)	3,530,531,046		3,535,795,546	
Total number (>100bp)	15,899		5,370	

Note: These data pertain to genome assembly. Wtdbg contig was the genome assembled by wtdbg and 2-round pilon error-correction. Hi-C scaffold was the genome finished by Hi-C assembly.

Table 3: Assessment of genome assembly and annotation completeness of the mustache toad and other amphibian genomes, using Benchmarking Universal Single-Copy Orthologs (BUSCO)

Library	*V. ailaonica* (eukaryota)	*V. ailaonica* (metazoa)	*Nanorana parkeri* (eukaryota)	*Xenopus tropicalis* (eukaryota)	*Rhinella marina* (eukaryota)	*Rana catesbeiana* (eukaryota)	*Ambystoma mexicanum* (eukaryota)
Complete genes (%)	80.8	85.1	90.1	90.1	90.4	58.0	24.4
Complete and single-copy	78.2	83.6	87.8	88.1	86.1	55.4	23.4

genes (%)							
Complete and duplicated genes (%)	2.6	1.5	2.3	2.0	4.3	2.6	1.0
Fragmented genes (%)	8.9	4.9	3.6	2.0	3.3	20.8	24.4
Missing genes (%)	10.3	10.0	6.3	7.9	6.3	21.2	51.2

Note: Both ‘eukaryote’ and ‘metazoan’ are two core gene sets in the BUSCO database

Table 4: Quality data for several published amphibian genomes

Species	Contig N50 (bp)	Scaffold N50 (bp)	Genome size (bp)	Genome BUSCO (eukaryota) (%)
Nanorana parkeri	32,798	1,069,101	2,053,867,363	90.1
Xenopus tropicalis	71,041	135,134,832	1,440,398,454	90.1
Rhinella marina	166,489	167,498	2,551,759,918	90.4
Rana catesbeiana	5,415	39,363	6,250,353,185	58.0
Ambystoma mexicanum	216,366	3,052,786	32,393,605,577	24.4

Table 5: D*e novo*-annotated repeat sequences in the mustache toad genome

Type	Length (bp)	Percentage in genome (%)

DNA	350,793,270	9.94
LINE	297,954,803	8.45
SINE	11,009,363	0.31
LTR	307,317,539	8.71
Other	43,867,330	1.24
Satellite	9,696,790	0.27
Simple repeat	125,397,072	3.55
Unknown	1,114,326,962	31.59
Total	2,147,505,764	60.87

Table 6: Functional annotation for protein-coding genes in the mustache toad genome

Database	Annotated gene number	Percent (%)
Interpro	12,997	49.56
KEGG	10,035	38.26
SwissProt	12,410	47.32
Trembl	17,916	68.31

Figure legends

Figure 1: The mustache toad, *Vibrissaphora ailaonica*.

(**A**) The adult male individual with spines in the upper jaw. (**B**) The adult female individual. (**C**) The adult male individual during the process of spines shedding from

the upper jaw. (**D**) The adult male individual without spines (after spine have been shed) in the upper jaw. (**E**) The body size of the mustache toad, side view: male (left) and female (right). (**F**) The body size of mustache toad, top view: male (left) and female (right).

Figure 2: 17-mer analysis of *Vibrissaphora ailaonica* genome characteristics.

Figure 3: Circos graph showing characteristics of the mustache toad genome. From outer circle to inner ring: gene distribution, tandem repeats (TR), long tandem repeats (LTR), long interspersed nuclear elements (LINE), short interspersed nuclear elements (SINE), and GC content.

Figure 4: Length distributions of annotated protein-coding genes in these species. The species including *Vibrissaphora ailaonica*, *Homo sapiens*, *Danio rerio*, *Gallus gallus*, *Anolis carolinensis*, *Alligator sinensis*, and *Nanorana parkeri*.

Figure 5: The statistics of gene family among these 11 species. The species including *Danio rerio*, *Rana catesbeiana*, *Rhinella marina*, *Vibrissaphora ailaonica*, *Nanorana parkeri*, *Homo sapiens*, *Gallus gallus*, *Anolis carolinensis*, *Xenopus tropicalis*, *Ambystoma mexicanum*, and *Alligator sinensis*.

Figure 6: The phylogenetic relationships among these species. The species

including *Danio rerio*, *Rana catesbeiana*, *Rhinella marina*, *Vibrissaphora ailaonica*, *Nanorana parkeri*, *Homo sapiens*, *Gallus gallus*, *Anolis carolinensis*, *Xenopus tropicalis*, *Ambystoma mexicanum*, and *Alligator sinensis*. Blue numbers represent divergence time. The red dot represents the fossil record used in the node.

Additional files

Additional Figure S1: The GC content in these genomes. The species including *Gallus gallus*, *Vibrissaphora ailaonica*, *Alligator sinensis*, *Nanorana parkeri*, *Homo sapiens*, *Anolis carolinensis*, *Xenopus tropicalis*, and *Danio rerio*.

Additional Figure S2: The divergence time of these species (using first-codon sites). The species including *Danio rerio*, *Rana catesbeiana*, *Rhinella marina*, *Vibrissaphora ailaonica*, *Nanorana parkeri*, *Homo sapiens*, *Gallus gallus*, *Anolis carolinensis*, *Xenopus tropicalis*, *Ambystoma mexicanum*, and *Alligator sinensis*.

Additional Figure S3: The divergence time of these species (using second-codon sites). The species including *Danio rerio*, *Rana catesbeiana*, *Rhinella marina*, *Vibrissaphora ailaonica*, *Nanorana parkeri*, *Homo sapiens*, *Gallus gallus*, *Anolis carolinensis*, *Xenopus tropicalis*, *Ambystoma mexicanum*, and *Alligator sinensis*.

Additional Table S1: Illumina sequencing clean data.

Additional Table S2: PacBio Sequel sequencing data.

Additional Table S3: RNA-sequencing clean data.

Additional Table S4: Hi-C sequencing clean data.

Additional Table S5: Comparison of the BUSCO and Illumina read mapping results

between the raw genome and redundancy-filtered genome.

Additional Table S6: Illumina read mapping ratio to the assembled genome.

Additional Table S7: The statistics of assembled transcripts by Bridger software. The redundant transcripts were removed by TGICL software.

Additional Table S8: Transcript mapping ratio to the assembled genome.

Additional Table S9: Annotated repeat sequences in our assembled genome.

Additional Table S10: Gene families among these species. The species including *Danio rerio*, *Rana catesbeiana*, *Rhinella marina*, *Vibrissaphora ailaonica*, *Nanorana parkeri*, *Homo sapiens*, *Gallus gallus*, *Anolis carolinensis*, *Xenopus tropicalis*, *Ambystoma mexicanum*, and *Alligator sinensis*.

Additional Table S11: Gene Ontology (GO) enrichment analysis of expanded gene families.

Additional Table S12: Kyoto Encyclopedia of Genes and Genomes (KEGG) enrichment analysis of expanded gene families.

Additional Table S13: Gene Ontology (GO) enrichment analysis of contracted gene families.

Additional Table S14: Kyoto Encyclopedia of Genes and Genomes (KEGG) enrichment analysis of contracted gene families.

Additional Table S15: Two-cluster analysis of mustache toad and other species.

Additional Table S16: The relative evolutionary rate of mustache toad and other species analyzed by Tajima's test.

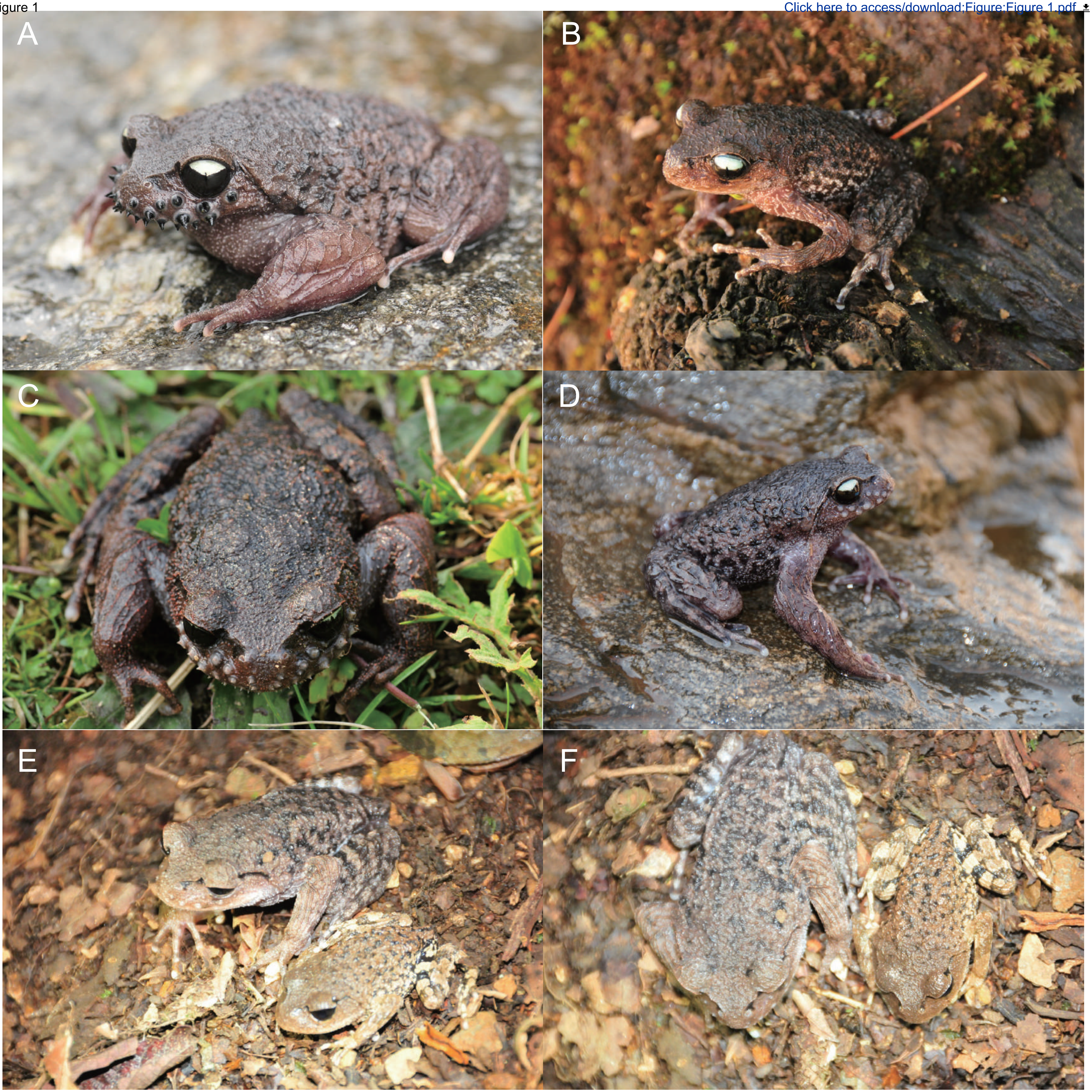
A
B
C
D
E
F

Figure 2

Click here to access/download;Figure;Figure 2.pdf

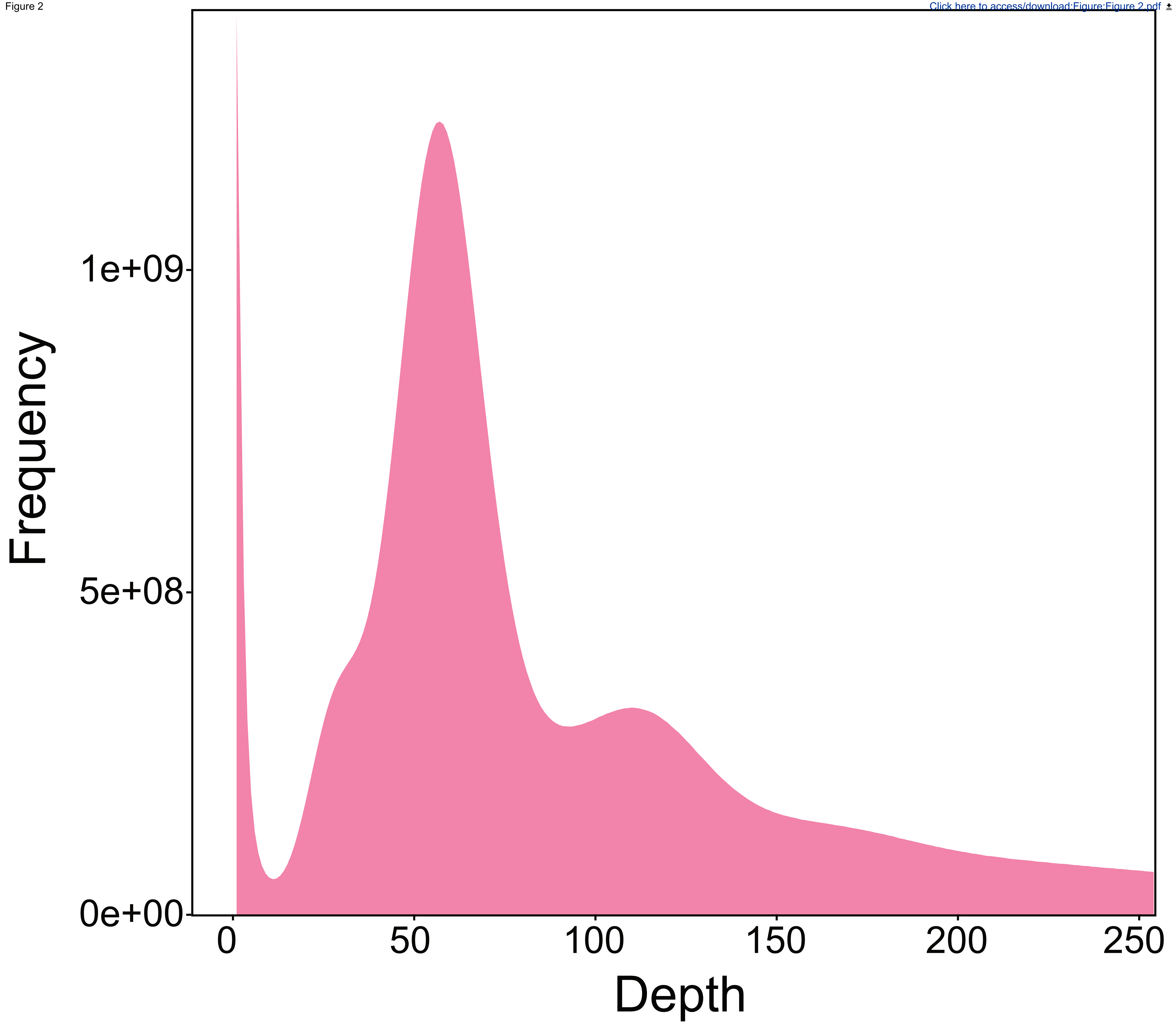

Figure 3

Click here to access/download;Figure;Figure 3.pdf

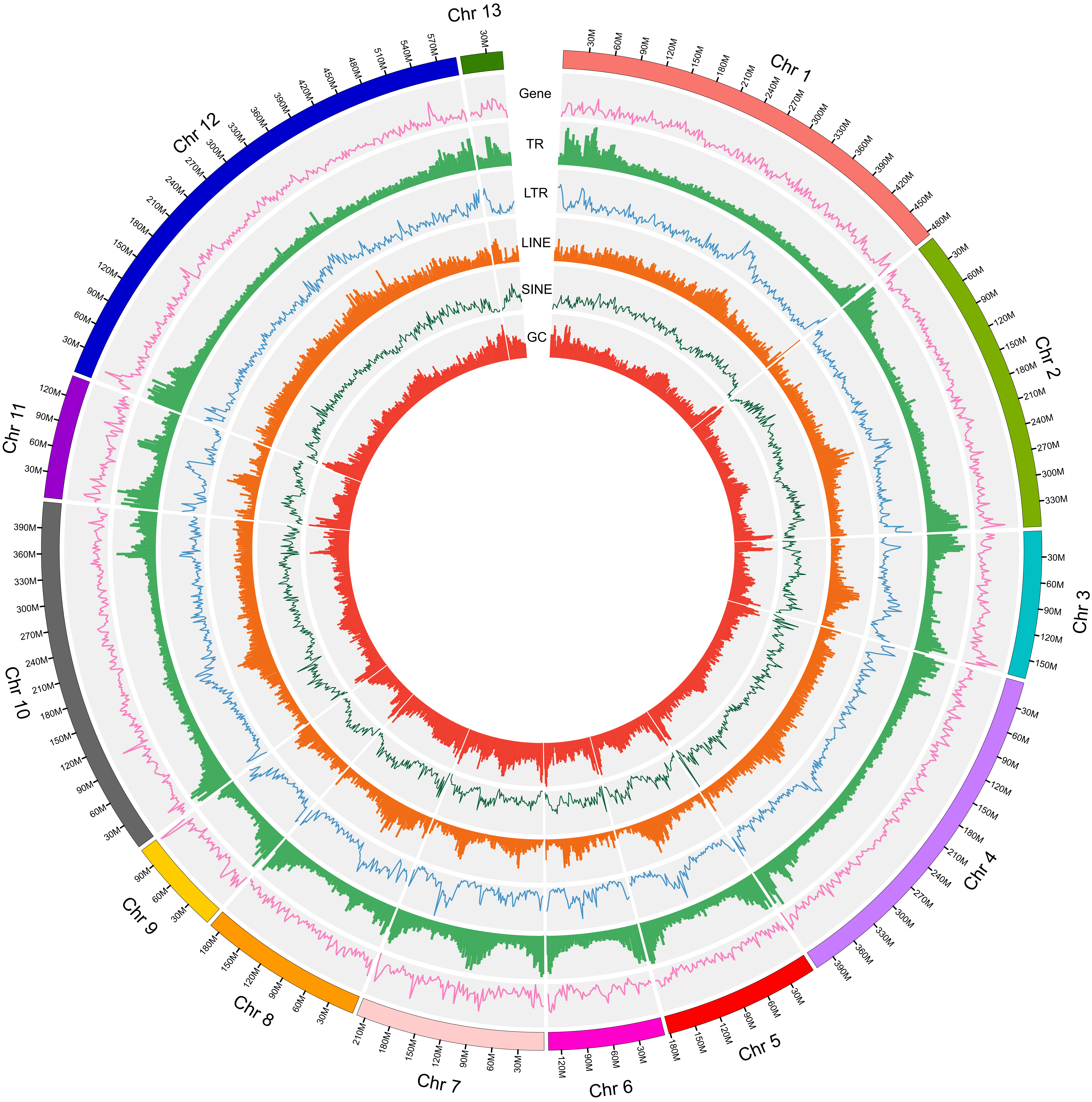

Figure 4

Click here to access/download;Figure;Figure 4.pdf

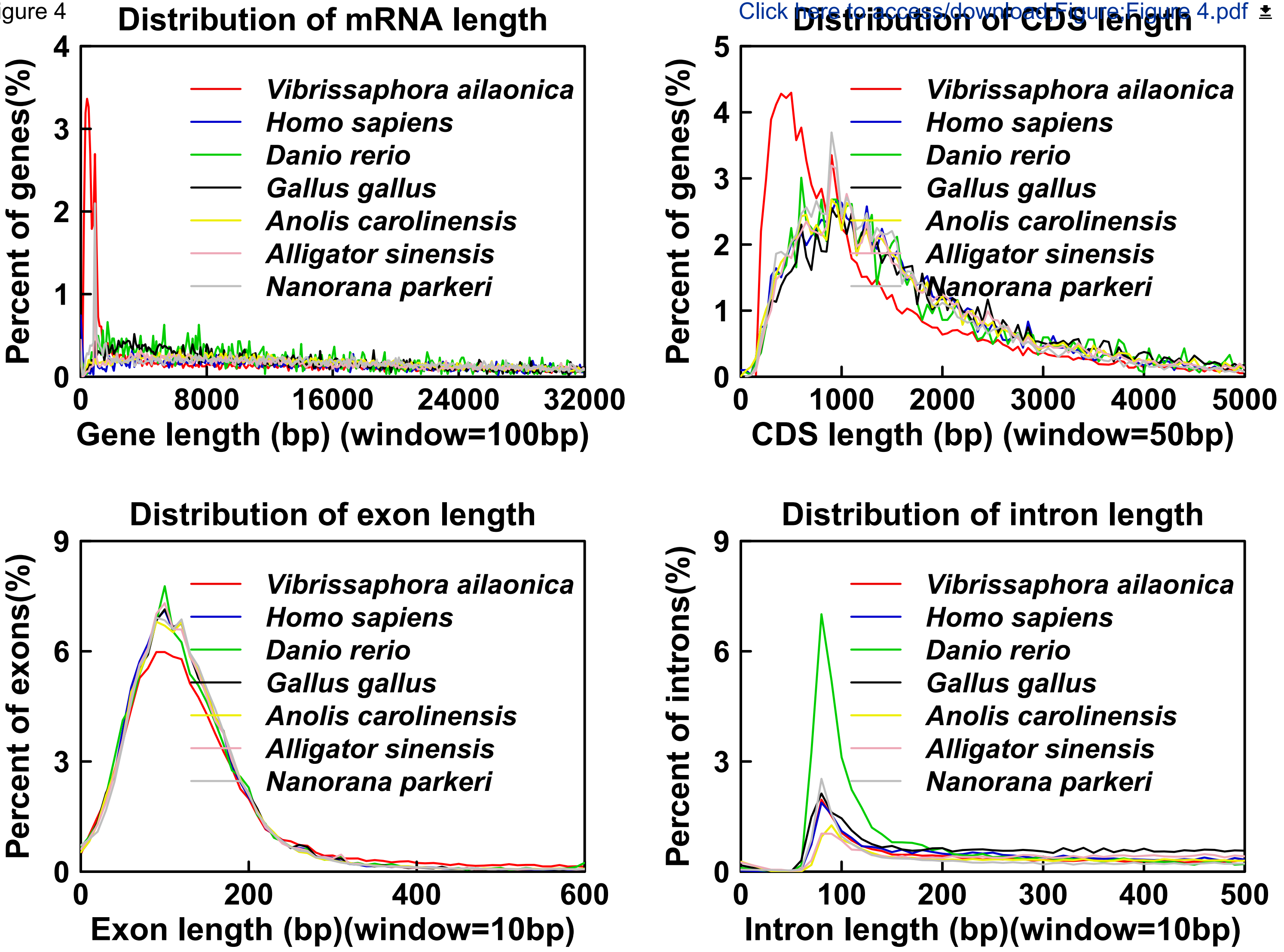

Click here to access/download;Figure;Figure 5.pdf

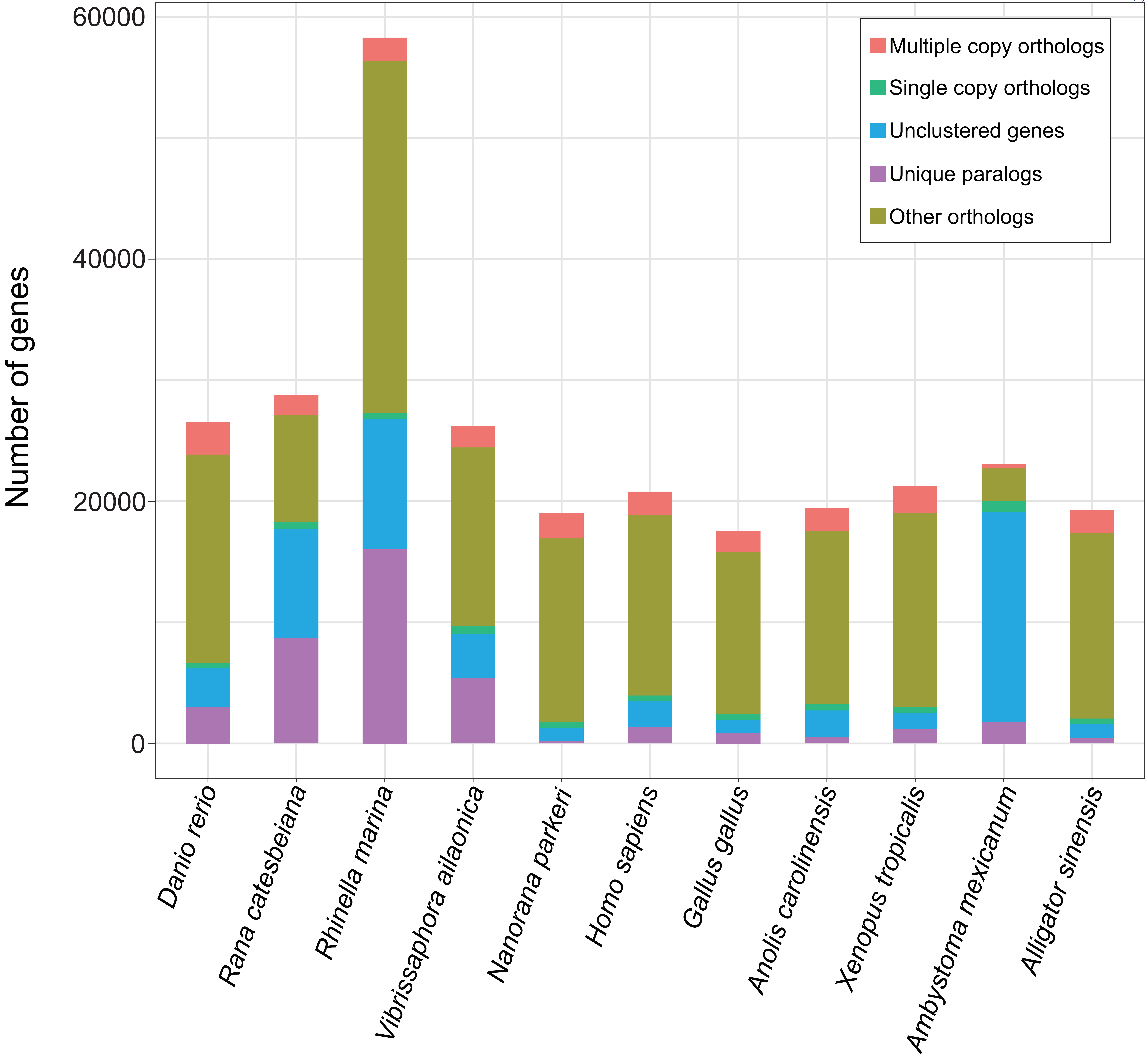

Figure 6

Click here to access/download;Figure;Figure 6.pdf

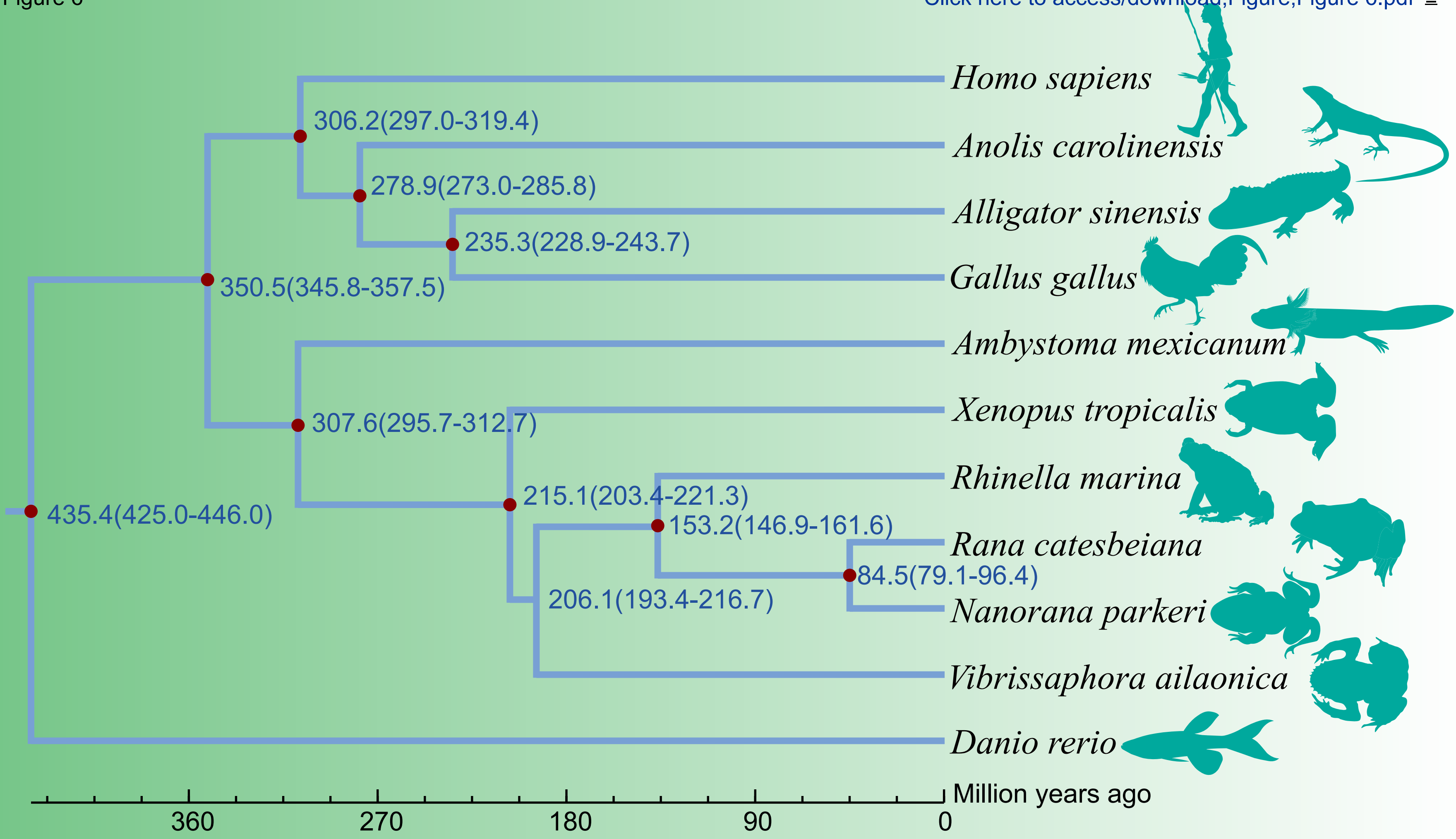

Click here to access/download
Supplementary Material
Supplementary Materials.doc
