# Supplementary material for: Chromosome-level assembly of the mustache toad genome using third-generation DNA sequencing and Hi-C analysis
Source: Gigascience. 2019 Sep 23;8(9):giz114. doi: 10.1093/gigascience/giz114 (PMC6755253; doi:10.1093/gigascience/giz114)
Supplement: giz114_Supplementary_Files [file giz114_supplementary_files.doc]

Table S1: Illumina sequencing clean data.

| Library ID | Total reads (PE) | Total bases (PE) | Sequencing strategy | Sequencing depth (X) |
| --- | --- | --- | --- | --- |
| Bank_220bp_1 | 582,833,836 | 73,413,385,936 | PE150 | 20.88 |
| Bank_220bp_2 | 283,865,412 | 35,764,217,296 | PE150 | 10.17 |
| Bank_220bp_3 | 287,593,388 | 36,233,971,934 | PE150 | 10.31 |
| Bank_500bp_1 | 284,006,014 | 35,782,045,588 | PE150 | 10.18 |
| Bank_500bp_2 | 347,945,268 | 43,837,759,016 | PE150 | 12.47 |
| Total | 1,786,243,918 | 225,031,379,770 | - | 64.01 |

Table S2: PacBio Sequel sequencing data.

| Cell | # of total subreads bases | # of subreads | Mean subreads length | Subreads N50 length |
| --- | --- | --- | --- | --- |
| m54061_180518_075056 | 7,979,227,224 | 955,606 | 8,349.91 | 13,320 |
| m54136_180519_070119 | 5,932,315,887 | 768,004 | 7,724.33 | 12,951 |
| m54139_180519_101818 | 7,499,768,690 | 899,036 | 8,342.01 | 13,401 |
| m54061_180519_203134 | 6,844,039,853 | 865,256 | 7,909.84 | 12,933 |
| m54061_180522_045401 | 7,510,230,267 | 896,502 | 8,377.26 | 13,707 |
| m54061_180523_082608 | 7,199,987,761 | 750,986 | 9,587.38 | 14,314 |
| m54136_180523_032127 | 7,733,351,812 | 915,520 | 8,446.95 | 13,570 |
| m54139_180523_053910 | 7,785,252,096 | 926,692 | 8,401.12 | 13,485 |
| m54136_180523_133425 | 7,501,812,794 | 904,643 | 8,292.57 | 13,405 |
| m54139_180523_155315 | 6,046,013,497 | 744,340 | 8,122.65 | 13,179 |
| m54139_180522_090200 | 6,469,561,980 | 812,383 | 7,963.68 | 13,132 |
| m54139_180521_064948 | 8,030,240,534 | 955,329 | 8,405.73 | 13,453 |
| m54061_180521_081500 | 6,751,306,731 | 883,034 | 7,645.58 | 13,122 |
| m54136_180524_034610 | 6,110,932,978 | 596,975 | 10,236.50 | 14,740 |
| m54139_180524_062124 | 7,186,230,537 | 720,974 | 9,967.39 | 14,519 |
| m54136_180524_135839 | 6,133,166,424 | 653,578 | 9,383.99 | 13,960 |
| m54139_180527_143742 | 6,401,985,648 | 676,649 | 9,461.31 | 13,992 |
| m54136_180525_110602 | 7,082,100,757 | 741,661 | 9,548.97 | 14,368 |
| m54061_180526_073150 | 6,860,233,113 | 718,011 | 9,554.50 | 14,324 |
| m54061_180525_110324 | 7,992,221,053 | 853,517 | 9,363.87 | 14,355 |
| m54139_180526_075913 | 7,518,315,568 | 683,131 | 11,005.67 | 16,491 |
| m54061_180525_211619 | 7,755,021,252 | 822,491 | 9,428.70 | 14,209 |
| m54136_180527_180845 | 8,032,412,777 | 723,374 | 11,104.09 | 16,927 |
| m54061_180527_181500 | 7,466,369,324 | 666,484 | 11,202.62 | 16,143 |
| m54136_180527_075637 | 6,978,299,693 | 614,013 | 11,365.07 | 16,684 |
| m54061_180528_144417 | 7,490,488,881 | 653,973 | 11,453.82 | 16,535 |
| m54061_180528_042925 | 7,645,926,962 | 688,754 | 11,101.10 | 16,402 |
| m54139_180528_082807 | 8,228,008,520 | 758,462 | 10,848.28 | 16,416 |
| m54136_180528_082751 | 8,158,506,415 | 739,499 | 11,032.48 | 16,640 |
| m54139_180530_063258 | 9,052,990,684 | 862,634 | 10,494.59 | 16,199 |
| m54139_180529_150238 | 5,188,257,643 | 454,202 | 11,422.80 | 16,603 |
| m54136_180529_045543 | 7,513,104,015 | 718,585 | 10,455.41 | 16,024 |
| m54139_180529_044957 | 8,450,722,595 | 783,322 | 10,788.31 | 16,374 |
| m54136_180528_184006 | 8,196,588,359 | 754,625 | 10,861.80 | 16,283 |
| m54139_180528_183731 | 7,355,521,665 | 710,184 | 10,357.21 | 15,923 |
| m54139_180530_164054 | 7,588,548,780 | 756,560 | 10,030.33 | 15,544 |
| m54139_180527_042705 | 6,165,300,864 | 656,771 | 9,387.29 | 13,917 |
| m54061_180526_174736 | 7,317,025,826 | 790,616 | 9,254.84 | 14,182 |
| Total/Average | 277,151,389,459 | 29,076,376 | 9,649 | 14,782 |

Table S3: RNA-sequencing clean data.

| Term | RNA-seq |
| --- | --- |
| Total reads (PE) | 95,477,170 |
| Total bases (PE) | 14,184,855,224 |
| Sequencing strategy | PE150 |

Table S4: Hi-C sequencing clean data.

| Library ID | Total reads (PE) | Total bases (PE) | Sequencing strategy | Sequencing depth (X) |
| --- | --- | --- | --- | --- |
| CC6 | 749,761,952 | 112,464,292,800 | PE150 | 31.99 |
| CC11 | 563,053,188 | 84,457,978,200 | PE150 | 24.02 |
| CC20 | 848,396,332 | 127,259,449,800 | PE150 | 36.19 |
| CC23 | 363,979,516 | 54,596,927,400 | PE150 | 15.53 |
| Total | 2,525,190,988 | 378,778,648,200 | - | 107.73 |

Table S5. Comparison of the BUSCO and Illumina reads mapping results between the raw genome and redundancy-filtered genome.

| Term | Raw genome | Redundancy-filtered genome |
| --- | --- | --- |
| BUSCO (eukaryota) | Complete: 79.9%; Fragmented: 11.2%;  Missing: 8.9% | Complete: 77.9 %; Fragmented: 11.2 %;  Missing: 10.9 % |
| BUSCO (metazoa) | Complete: 84.7%; Fragmented: 5.5%;  Missing: 9.8% | Complete: 82.9%; Fragmented: 4.7%;  Missing: 12.4% |
| Mapping number of Illumina reads | 1,788,735,503 | 1,780,194,139 |
| Mapping ratio of Illumina reads | 98.40% | 97.71% |

Table S6: Illumina reads mapping ratio to the assembled genome.

| Term | Number |
| --- | --- |
| Total reads | 1,818,733,600 |
| Mapped reads to genome | 1,778,381,152 |
| Percent (%) | 97.78 |

Table S7: The statistics of assembled transcripts by Bridger software. The redundant transcripts were removed by TGICL software.

| Term | Size (bp) | Number |
| --- | --- | --- |
| N90 | 737 | 13,149 |
| N80 | 1,121 | 9,584 |
| N70 | 1,560 | 7,121 |
| N60 | 2,023 | 5,289 |
| N50 | 2,528 | 3,850 |
| Max length (bp) | 19,054 | |
| Total size (bp) | 32,614,892 | |
| Total number (>100bp) | 19,876 | |
| Total number (>1000 bp) | 10,528 | |

Table S8: Transcript mapping ratio to the assembled genome.

| Range of length | Total number | Total match number | Percent  (%) | >50% of sequence | | >90% of sequence | |
| --- | --- | --- | --- | --- | --- | --- | --- |
|  |  |  |  | Number | Percent  (%) | Number | Percent  (%) |
| All | 19,876 | 17,878 | 89.95 | 15,747 | 79.23 | 8,189 | 41.20 |
| ≥500 | 16,382 | 15,041 | 91.81 | 13,171 | 80.40 | 6,569 | 40.10 |
| ≥1000 | 10,528 | 9,951 | 94.52 | 8,609 | 81.77 | 4,190 | 39.80 |

Table S9: Annotated repeat sequences in our assembled genome. The type represents that the method or software used in this study.

| Type | Repeat Size | % of genome |
| --- | --- | --- |
| Trf | 706,824,074 | 20.036021 |
| Repeatmasker | 285,988,783 | 8.106794 |
| Proteinmask | 358,644,435 | 10.166331 |
| *De novo* | 2,147,505,764 | 60.874369 |
| Total | 2,451,228,978 | 69.483873 |

Table S10: Gene families among these species. The species including *Danio rerio*, *Rana catesbeiana*, *Rhinella marina*, *Vibrissaphora ailaonica*, *Nanorana parkeri*, *Homo sapiens*, *Gallus gallus*, *Anolis carolinensis*, *Xenopus tropicalis*, *Ambystoma mexicanum*, and *Alligator sinensis*.

| Species | Genes number | Genes in families | Unclustered genes | Family number | Unique families | Average genes  per family |
| --- | --- | --- | --- | --- | --- | --- |
| [*Danio rerio*](https://www.ncbi.nlm.nih.gov/Taxonomy/Browser/wwwtax.cgi?mode=Info&id=7955&lvl=3&lin=f&keep=1&srchmode=1&unlock) | 26,533 | 23,318 | 3,215 | 13,025 | 468 | 1.79 |
| *Vibrissaphora ailaonica* | 26,227 | 22,557 | 3,670 | 10,024 | 593 | 2.25 |
| [*Nanorana parkeri*](https://www.ncbi.nlm.nih.gov/Taxonomy/Browser/wwwtax.cgi?mode=Info&id=125878&lvl=3&lin=f&keep=1&srchmode=1&unlock) | 19,019 | 17,928 | 1,091 | 13,555 | 71 | 1.32 |
| [*Homo sapiens*](https://www.ncbi.nlm.nih.gov/Taxonomy/Browser/wwwtax.cgi?mode=Info&id=9606&lvl=3&lin=f&keep=1&srchmode=1&unlock) | 20,799 | 18,688 | 2,111 | 13,465 | 314 | 1.39 |
| [*Gallus gallus*](https://www.ncbi.nlm.nih.gov/Taxonomy/Browser/wwwtax.cgi?mode=Info&id=9031&lvl=3&lin=f&keep=1&srchmode=1&unlock) | 17,563 | 16,497 | 1,066 | 12,436 | 97 | 1.33 |
| [*Anolis carolinensis*](https://www.ncbi.nlm.nih.gov/Taxonomy/Browser/wwwtax.cgi?mode=Info&id=28377&lvl=3&lin=f&keep=1&srchmode=1&unlock) | 19,417 | 17,200 | 2,217 | 12,915 | 121 | 1.33 |
| [*Xenopus tropicalis*](https://www.ncbi.nlm.nih.gov/Taxonomy/Browser/wwwtax.cgi?mode=Info&id=8364&lvl=3&lin=f&keep=1&srchmode=1&unlock) | 21,258 | 19,924 | 1,334 | 13,926 | 236 | 1.43 |
| [*Alligator sinensis*](https://www.ncbi.nlm.nih.gov/Taxonomy/Browser/wwwtax.cgi?mode=Info&id=38654&lvl=3&lin=f&keep=1&srchmode=1&unlock) | 19,310 | 18,160 | 1,150 | 13,602 | 115 | 1.34 |
| *Rhinella marina* | 58,302 | 47,550 | 10,752 | 15,025 | 1,658 | 3.16 |
| *Rana catesbeiana* | 28,771 | 19,762 | 9,009 | 7,213 | 706 | 2.74 |
| *Ambystoma mexicanum* | 23,106 | 5,734 | 17,372 | 3,887 | 571 | 1.48 |

Table S11: Gene Ontology (GO) enrichment analysis of expanded gene families.

| GO ID | GO Term | GO Class | *P*-value | Adjusted *P*-value |
| --- | --- | --- | --- | --- |
| GO:0008150 | biological process | BP | 8.41E-59 | 5.85E-56 |
| GO:0009987 | cellular process | BP | 1.93E-41 | 6.71E-39 |
| GO:0008270 | zinc ion binding | MF | 3.41E-40 | 7.92E-38 |
| GO:0003676 | nucleic acid binding | MF | 2.71E-36 | 4.72E-34 |
| GO:0046914 | transition metal ion binding | MF | 2.34E-33 | 3.26E-31 |
| GO:0000786 | nucleosome | CC | 4.65E-25 | 4.62E-23 |
| GO:0032993 | protein-DNA complex | CC | 4.65E-25 | 4.62E-23 |
| GO:0046983 | protein dimerization activity | MF | 9.22E-25 | 8.02E-23 |
| GO:0000785 | chromatin | CC | 1.32E-23 | 1.02E-21 |
| GO:0046982 | protein heterodimerization activity | MF | 3.79E-23 | 2.63E-21 |
| GO:0008152 | metabolic process | BP | 2.47E-21 | 1.56E-19 |
| GO:0044699 | single-organism process | BP | 1.80E-20 | 1.04E-18 |
| GO:0044427 | chromosomal part | CC | 1.96E-19 | 1.05E-17 |
| GO:0044763 | single-organism cellular process | BP | 3.06E-18 | 1.52E-16 |
| GO:0005488 | binding | MF | 3.71E-17 | 1.72E-15 |
| GO:1901363 | heterocyclic compound binding | MF | 4.41E-17 | 1.92E-15 |
| GO:0097159 | organic cyclic compound binding | MF | 4.74E-17 | 1.94E-15 |
| GO:0005694 | chromosome | CC | 8.76E-17 | 3.39E-15 |
| GO:0005882 | intermediate filament | CC | 9.84E-17 | 3.42E-15 |
| GO:0045111 | intermediate filament cytoskeleton | CC | 9.84E-17 | 3.42E-15 |
| GO:0044237 | cellular metabolic process | BP | 1.83E-15 | 6.06E-14 |
| GO:0044238 | primary metabolic process | BP | 4.12E-15 | 1.30E-13 |
| GO:0071704 | organic substance metabolic process | BP | 6.68E-15 | 2.02E-13 |
| GO:0045095 | keratin filament | CC | 1.01E-14 | 2.94E-13 |
| GO:0003824 | catalytic activity | MF | 3.29E-13 | 9.17E-12 |
| GO:0004523 | ribonuclease H activity | MF | 4.46E-13 | 1.19E-11 |
| GO:0005622 | intracellular | CC | 1.50E-12 | 3.87E-11 |
| GO:0005623 | cell | CC | 4.28E-12 | 1.03E-10 |
| GO:0044464 | cell part | CC | 4.28E-12 | 1.03E-10 |
| GO:0065007 | biological regulation | BP | 7.77E-12 | 1.80E-10 |
| GO:0050789 | regulation of biological process | BP | 1.15E-11 | 2.58E-10 |
| GO:0050794 | regulation of cellular process | BP | 2.94E-11 | 6.39E-10 |
| GO:0051716 | cellular response to stimulus | BP | 3.44E-11 | 7.27E-10 |
| GO:0051234 | establishment of localization | BP | 1.02E-10 | 2.08E-09 |
| GO:0006810 | transport | BP | 1.07E-10 | 2.14E-09 |
| GO:0051179 | localization | BP | 1.13E-10 | 2.19E-09 |
| GO:0007154 | cell communication | BP | 1.47E-10 | 2.77E-09 |
| GO:0046872 | metal ion binding | MF | 2.62E-10 | 4.80E-09 |
| GO:0003677 | DNA binding | MF | 2.94E-10 | 5.25E-09 |
| GO:0023052 | signaling | BP | 4.10E-10 | 7.14E-09 |
| GO:0044700 | single organism signaling | BP | 4.54E-10 | 7.71E-09 |
| GO:0007165 | signal transduction | BP | 6.48E-10 | 1.07E-08 |
| GO:0043169 | cation binding | MF | 6.94E-10 | 1.12E-08 |
| GO:0044446 | intracellular organelle part | CC | 1.37E-09 | 2.17E-08 |
| GO:0016891 | endoribonuclease activity, producing 5'-phosphomonoesters | MF | 1.45E-09 | 2.24E-08 |
| GO:0044422 | organelle part | CC | 1.58E-09 | 2.40E-08 |
| GO:0044260 | cellular macromolecule metabolic process | BP | 1.76E-09 | 2.61E-08 |
| GO:0043170 | macromolecule metabolic process | BP | 2.14E-09 | 3.11E-08 |
| GO:0003674 | molecular_function | MF | 2.47E-09 | 3.52E-08 |
| GO:0016020 | membrane | CC | 2.68E-09 | 3.73E-08 |
| GO:0031224 | intrinsic to membrane | CC | 3.48E-09 | 4.75E-08 |
| GO:0016021 | integral to membrane | CC | 3.66E-09 | 4.90E-08 |
| GO:0044710 | single-organism metabolic process | BP | 4.88E-09 | 6.40E-08 |
| GO:0005525 | GTP binding | MF | 7.40E-09 | 9.16E-08 |
| GO:0019001 | guanyl nucleotide binding | MF | 7.40E-09 | 9.16E-08 |
| GO:0032561 | guanyl ribonucleotide binding | MF | 7.40E-09 | 9.16E-08 |
| GO:0050896 | response to stimulus | BP | 7.50E-09 | 9.16E-08 |
| GO:0004521 | endoribonuclease activity | MF | 7.79E-09 | 9.19E-08 |
| GO:0016893 | endonuclease activity, active with either ribo- or deoxyribonucleic acids and producing 5'-phosphomonoesters | MF | 7.79E-09 | 9.19E-08 |
| GO:0044425 | membrane part | CC | 1.15E-08 | 1.33E-07 |
| GO:0043228 | non-membrane-bounded organelle | CC | 4.72E-08 | 5.30E-07 |
| GO:0043232 | intracellular non-membrane-bounded organelle | CC | 4.72E-08 | 5.30E-07 |
| GO:0030554 | adenyl nucleotide binding | MF | 7.21E-08 | 7.97E-07 |
| GO:0032559 | adenyl ribonucleotide binding | MF | 7.58E-08 | 8.24E-07 |
| GO:0005524 | ATP binding | MF | 8.36E-08 | 8.95E-07 |
| GO:0004540 | ribonuclease activity | MF | 1.14E-07 | 1.21E-06 |
| GO:0035556 | intracellular signal transduction | BP | 1.21E-07 | 1.25E-06 |
| GO:0044765 | single-organism transport | BP | 1.34E-07 | 1.37E-06 |
| GO:0005737 | cytoplasm | CC | 3.11E-07 | 3.14E-06 |
| GO:0005215 | transporter activity | MF | 3.81E-07 | 3.79E-06 |
| GO:0044430 | cytoskeletal part | CC | 3.89E-07 | 3.81E-06 |
| GO:0004519 | endonuclease activity | MF | 9.26E-07 | 8.95E-06 |
| GO:1901360 | organic cyclic compound metabolic process | BP | 1.05E-06 | 1.00E-05 |
| GO:0006807 | nitrogen compound metabolic process | BP | 1.28E-06 | 1.21E-05 |
| GO:0019538 | protein metabolic process | BP | 1.32E-06 | 1.22E-05 |
| GO:0044267 | cellular protein metabolic process | BP | 1.78E-06 | 1.63E-05 |
| GO:0006725 | cellular aromatic compound metabolic process | BP | 2.03E-06 | 1.84E-05 |
| GO:0046483 | heterocycle metabolic process | BP | 2.12E-06 | 1.89E-05 |
| GO:0034641 | cellular nitrogen compound metabolic process | BP | 3.01E-06 | 2.65E-05 |
| GO:0016491 | oxidoreductase activity | MF | 3.41E-06 | 2.96E-05 |
| GO:0022892 | substrate-specific transporter activity | MF | 5.99E-06 | 5.15E-05 |
| GO:0006139 | nucleobase-containing compound metabolic process | BP | 8.04E-06 | 6.82E-05 |
| GO:0022857 | transmembrane transporter activity | MF | 1.12E-05 | 9.36E-05 |
| GO:0006811 | ion transport | BP | 1.21E-05 | 0.000100112 |
| GO:0044281 | small molecule metabolic process | BP | 1.40E-05 | 0.00011431 |
| GO:0006352 | DNA-dependent transcription, initiation | BP | 1.92E-05 | 0.000155307 |
| GO:0022891 | substrate-specific transmembrane transporter activity | MF | 2.76E-05 | 0.000220781 |
| GO:0006793 | phosphorus metabolic process | BP | 3.02E-05 | 0.000239001 |
| GO:0006796 | phosphate-containing compound metabolic process | BP | 3.60E-05 | 0.000281842 |
| GO:0007264 | small GTPase mediated signal transduction | BP | 4.61E-05 | 0.000356823 |
| GO:0055085 | transmembrane transport | BP | 5.04E-05 | 0.000385176 |
| GO:0015075 | ion transmembrane transporter activity | MF | 5.44E-05 | 0.00041123 |
| GO:0055114 | oxidation-reduction process | BP | 6.84E-05 | 0.000511737 |
| GO:0003700 | sequence-specific DNA binding transcription factor activity | MF | 0.0001059 | 0.000775857 |
| GO:0001071 | nucleic acid binding transcription factor activity | MF | 0.0001059 | 0.000775857 |
| GO:0016787 | hydrolase activity | MF | 0.000108802 | 0.000778141 |
| GO:0042605 | peptide antigen binding | MF | 0.000110684 | 0.000778141 |
| GO:0042612 | MHC class I protein complex | CC | 0.000110684 | 0.000778141 |
| GO:0003823 | antigen binding | MF | 0.000110684 | 0.000778141 |
| GO:0006323 | DNA packaging | BP | 0.000117618 | 0.000818622 |
| GO:0009058 | biosynthetic process | BP | 0.000130898 | 0.000902027 |
| GO:0036094 | small molecule binding | MF | 0.0001344 | 0.000917084 |
| GO:0042611 | MHC protein complex | CC | 0.000158588 | 0.001071624 |
| GO:0005856 | cytoskeleton | CC | 0.000171959 | 0.0011508 |
| GO:0010467 | gene expression | BP | 0.000175271 | 0.001161794 |
| GO:0002474 | antigen processing and presentation of peptide antigen via MHC class I | BP | 0.000190967 | 0.001242178 |
| GO:0048002 | antigen processing and presentation of peptide antigen | BP | 0.000190967 | 0.001242178 |
| GO:0006812 | cation transport | BP | 0.000206665 | 0.001331841 |
| GO:0006334 | nucleosome assembly | BP | 0.000240184 | 0.001506017 |
| GO:0034728 | nucleosome organization | BP | 0.000240184 | 0.001506017 |
| GO:0031497 | chromatin assembly | BP | 0.000240184 | 0.001506017 |
| GO:0019882 | antigen processing and presentation | BP | 0.00024296 | 0.001509821 |
| GO:1901576 | organic substance biosynthetic process | BP | 0.000254373 | 0.001566759 |
| GO:0000166 | nucleotide binding | MF | 0.000262481 | 0.001588579 |
| GO:1901265 | nucleoside phosphate binding | MF | 0.000262481 | 0.001588579 |
| GO:0044249 | cellular biosynthetic process | BP | 0.000266024 | 0.001596145 |
| GO:0008324 | cation transmembrane transporter activity | MF | 0.000279294 | 0.001661443 |
| GO:0071103 | DNA conformation change | BP | 0.000281708 | 0.001661598 |
| GO:0006412 | translation | BP | 0.000301269 | 0.001762046 |
| GO:0006333 | chromatin assembly or disassembly | BP | 0.000325418 | 0.001887424 |
| GO:0090304 | nucleic acid metabolic process | BP | 0.00034629 | 0.001991882 |
| GO:0044444 | cytoplasmic part | CC | 0.000363788 | 0.002075382 |
| GO:0065004 | protein-DNA complex assembly | BP | 0.000434469 | 0.002438634 |
| GO:0071824 | protein-DNA complex subunit organization | BP | 0.000434469 | 0.002438634 |
| GO:0043167 | ion binding | MF | 0.000499425 | 0.002780797 |
| GO:0016702 | oxidoreductase activity, acting on single donors with incorporation of molecular oxygen, incorporation of two atoms of oxygen | MF | 0.000521286 | 0.002879483 |
| GO:0009056 | catabolic process | BP | 0.000550352 | 0.003016102 |
| GO:0004518 | nuclease activity | MF | 0.000713935 | 0.003882022 |
| GO:0043565 | sequence-specific DNA binding | MF | 0.000732687 | 0.003934919 |
| GO:0016701 | oxidoreductase activity, acting on single donors with incorporation of molecular oxygen | MF | 0.00073497 | 0.003934919 |
| GO:0016772 | transferase activity, transferring phosphorus-containing groups | MF | 0.000747804 | 0.003973068 |
| GO:0007166 | cell surface receptor signaling pathway | BP | 0.000790999 | 0.00417072 |
| GO:1901575 | organic substance catabolic process | BP | 0.000946972 | 0.004955581 |
| GO:0042277 | peptide binding | MF | 0.001073465 | 0.005484515 |
| GO:0006486 | protein glycosylation | BP | 0.001079567 | 0.005484515 |
| GO:0043413 | macromolecule glycosylation | BP | 0.001079567 | 0.005484515 |
| GO:0070085 | glycosylation | BP | 0.001079567 | 0.005484515 |
| GO:0005515 | protein binding | MF | 0.00108785 | 0.005486548 |
| GO:0003723 | RNA binding | MF | 0.001114179 | 0.005578908 |
| GO:0004871 | signal transducer activity | MF | 0.001143126 | 0.005642664 |
| GO:0060089 | molecular transducer activity | MF | 0.001143126 | 0.005642664 |
| GO:0015672 | monovalent inorganic cation transport | BP | 0.001229103 | 0.006024334 |
| GO:0022890 | inorganic cation transmembrane transporter activity | MF | 0.001248827 | 0.006078208 |
| GO:0019637 | organophosphate metabolic process | BP | 0.001262457 | 0.006101875 |
| GO:0051649 | establishment of localization in cell | BP | 0.001489352 | 0.007148892 |
| GO:0006955 | immune response | BP | 0.001753606 | 0.008359656 |
| GO:0009100 | glycoprotein metabolic process | BP | 0.002085482 | 0.009746732 |
| GO:0009101 | glycoprotein biosynthetic process | BP | 0.002085482 | 0.009746732 |
| GO:0008373 | sialyltransferase activity | MF | 0.002100589 | 0.009746732 |
| GO:0033218 | amide binding | MF | 0.002100589 | 0.009746732 |
| GO:1901564 | organonitrogen compound metabolic process | BP | 0.002131224 | 0.009823391 |
| GO:0005216 | ion channel activity | MF | 0.002239809 | 0.010057466 |
| GO:0015267 | channel activity | MF | 0.002239809 | 0.010057466 |
| GO:0022803 | passive transmembrane transporter activity | MF | 0.002239809 | 0.010057466 |
| GO:0022838 | substrate-specific channel activity | MF | 0.002239809 | 0.010057466 |
| GO:0051641 | cellular localization | BP | 0.002376354 | 0.010558427 |
| GO:0051213 | dioxygenase activity | MF | 0.002381714 | 0.010558427 |
| GO:0008092 | cytoskeletal protein binding | MF | 0.002407672 | 0.010605949 |
| GO:0016773 | phosphotransferase activity, alcohol group as acceptor | MF | 0.0024524 | 0.010735036 |
| GO:0006082 | organic acid metabolic process | BP | 0.002508238 | 0.010843064 |
| GO:0043436 | oxoacid metabolic process | BP | 0.002508238 | 0.010843064 |
| GO:0030529 | ribonucleoprotein complex | CC | 0.002730231 | 0.011729883 |
| GO:0032501 | multicellular organismal process | BP | 0.003014032 | 0.012869731 |
| GO:0008080 | N-acetyltransferase activity | MF | 0.003067803 | 0.013019455 |
| GO:0019752 | carboxylic acid metabolic process | BP | 0.003285277 | 0.013857895 |
| GO:0043412 | macromolecule modification | BP | 0.003392636 | 0.014184401 |
| GO:0042578 | phosphoric ester hydrolase activity | MF | 0.003403441 | 0.014184401 |
| GO:0002376 | immune system process | BP | 0.003720011 | 0.015411476 |
| GO:0008378 | galactosyltransferase activity | MF | 0.003757496 | 0.015452014 |
| GO:0044707 | single-multicellular organism process | BP | 0.003774199 | 0.015452014 |
| GO:0016410 | N-acyltransferase activity | MF | 0.003898241 | 0.015866525 |
| GO:0005509 | calcium ion binding | MF | 0.004324597 | 0.017499531 |
| GO:0038023 | signaling receptor activity | MF | 0.00469394 | 0.018884292 |
| GO:0043168 | anion binding | MF | 0.004773212 | 0.01909285 |
| GO:0006464 | cellular protein modification process | BP | 0.005511974 | 0.021797351 |
| GO:0036211 | protein modification process | BP | 0.005511974 | 0.021797351 |
| GO:0043231 | intracellular membrane-bounded organelle | CC | 0.005664249 | 0.022147851 |
| GO:0043227 | membrane-bounded organelle | CC | 0.005664249 | 0.022147851 |
| GO:0004872 | receptor activity | MF | 0.006164559 | 0.02396946 |
| GO:0006950 | response to stress | BP | 0.006269638 | 0.0242426 |
| GO:0008104 | protein localization | BP | 0.006593795 | 0.025300364 |
| GO:0005102 | receptor binding | MF | 0.0066159 | 0.025300364 |
| GO:0016301 | kinase activity | MF | 0.007389753 | 0.028105291 |
| GO:0019062 | viral attachment to host cell | BP | 0.008601676 | 0.031844503 |
| GO:0044406 | adhesion to host | BP | 0.008601676 | 0.031844503 |
| GO:0044650 | adhesion of symbiont to host cell | BP | 0.008601676 | 0.031844503 |
| GO:0051701 | interaction with host | BP | 0.008601676 | 0.031844503 |
| GO:0051825 | adhesion to other organism involved in symbiotic interaction | BP | 0.008601676 | 0.031844503 |
| GO:0016874 | ligase activity | MF | 0.008686535 | 0.031988511 |
| GO:0060589 | nucleoside-triphosphatase regulator activity | MF | 0.009178929 | 0.033447825 |
| GO:0055086 | nucleobase-containing small molecule metabolic process | BP | 0.009178929 | 0.033447825 |
| GO:0030695 | GTPase regulator activity | MF | 0.009699259 | 0.035153854 |
| GO:0006325 | chromatin organization | BP | 0.009748123 | 0.035153854 |
| GO:0071702 | organic substance transport | BP | 0.009904054 | 0.035532069 |
| GO:0048037 | cofactor binding | MF | 0.01016473 | 0.036199233 |
| GO:0003735 | structural constituent of ribosome | MF | 0.010249112 | 0.036199233 |
| GO:0005840 | ribosome | CC | 0.010249112 | 0.036199233 |
| GO:0015077 | monovalent inorganic cation transmembrane transporter activity | MF | 0.010298058 | 0.036199233 |
| GO:0016070 | RNA metabolic process | BP | 0.01039843 | 0.036368377 |
| GO:0006508 | proteolysis | BP | 0.010615396 | 0.036941578 |
| GO:0016705 | oxidoreductase activity, acting on paired donors, with incorporation or reduction of molecular oxygen | MF | 0.010830165 | 0.037501466 |
| GO:0016407 | acetyltransferase activity | MF | 0.01089818 | 0.037550164 |
| GO:0019222 | regulation of metabolic process | BP | 0.011410218 | 0.039120749 |
| GO:0030173 | integral to Golgi membrane | CC | 0.012853218 | 0.043450982 |
| GO:0031228 | intrinsic to Golgi membrane | CC | 0.012853218 | 0.043450982 |
| GO:0044424 | intracellular part | CC | 0.012860492 | 0.043450982 |
| GO:0007186 | G-protein coupled receptor signaling pathway | BP | 0.013183913 | 0.04432852 |
| GO:0031323 | regulation of cellular metabolic process | BP | 0.014121311 | 0.047252078 |
| GO:0006520 | cellular amino acid metabolic process | BP | 0.014269127 | 0.04751824 |
| GO:0016758 | transferase activity, transferring hexosyl groups | MF | 0.014912572 | 0.049424524 |

Table S12: Kyoto Encyclopedia of Genes and Genomes (KEGG) enrichment analysis of expanded gene families.

| Map number | Pathway | Count | *P*-value | *Q*-value |
| --- | --- | --- | --- | --- |
| map04610 | Complement and coagulation cascades | 49 | 4.70E-06 | 0.000128341 |
| map04514 | Cell adhesion molecules (CAMs) | 45 | 0.000264485 | 0.006268119 |
| map04621 | NOD-like receptor signaling pathway | 26 | 0.000280282 | 0.006268119 |
| map02030 | Bacterial chemotaxis | 7 | 0.000314728 | 0.006451926 |
| map04150 | mTOR signaling pathway | 24 | 0.000466902 | 0.008835226 |
| map00100 | Steroid biosynthesis | 13 | 0.000995962 | 0.017500467 |
| map04720 | Long-term potentiation | 30 | 0.001381201 | 0.022651689 |
| map05322 | Systemic lupus erythematosus | 29 | 0.001484947 | 0.022831053 |
| map05416 | Viral myocarditis | 25 | 0.003349423 | 0.048468114 |

Table S13: Gene Ontology (GO) enrichment analysis of contracted gene families.

| GO ID | GO Term | GO Class | *P*-value | Adjusted *P*-value |
| --- | --- | --- | --- | --- |
| GO:0016021 | integral to membrane | CC | 7.80E-31 | 1.72E-28 |
| GO:0031224 | intrinsic to membrane | CC | 8.83E-31 | 1.72E-28 |
| GO:0004930 | G-protein coupled receptor activity | MF | 1.56E-29 | 2.03E-27 |
| GO:0004888 | transmembrane signaling receptor activity | MF | 5.95E-27 | 5.81E-25 |
| GO:0071944 | cell periphery | CC | 1.00E-26 | 7.83E-25 |
| GO:0007186 | G-protein coupled receptor signaling pathway | BP | 1.98E-25 | 1.29E-23 |
| GO:0044425 | membrane part | CC | 2.97E-25 | 1.65E-23 |
| GO:0016020 | membrane | CC | 4.64E-24 | 2.26E-22 |
| GO:0038023 | signaling receptor activity | MF | 9.57E-24 | 4.15E-22 |
| GO:0007166 | cell surface receptor signaling pathway | BP | 1.94E-23 | 7.57E-22 |
| GO:0004872 | receptor activity | MF | 1.33E-22 | 4.73E-21 |
| GO:0044699 | single-organism process | BP | 4.04E-22 | 1.31E-20 |
| GO:0017111 | nucleoside-triphosphatase activity | MF | 1.63E-21 | 4.90E-20 |
| GO:0004871 | signal transducer activity | MF | 3.68E-21 | 9.58E-20 |
| GO:0060089 | molecular transducer activity | MF | 3.68E-21 | 9.58E-20 |
| GO:0016817 | hydrolase activity, acting on acid anhydrides | MF | 1.55E-20 | 3.77E-19 |
| GO:0016462 | pyrophosphatase activity | MF | 1.64E-20 | 3.77E-19 |
| GO:0016818 | hydrolase activity, acting on acid anhydrides, in phosphorus-containing anhydrides | MF | 2.69E-20 | 5.82E-19 |
| GO:0007156 | homophilic cell adhesion | BP | 6.00E-19 | 1.17E-17 |
| GO:0016337 | cell-cell adhesion | BP | 6.00E-19 | 1.17E-17 |
| GO:0005575 | cellular_component | CC | 6.25E-18 | 1.16E-16 |
| GO:0044763 | single-organism cellular process | BP | 5.44E-16 | 9.64E-15 |
| GO:0020037 | heme binding | MF | 1.73E-15 | 2.82E-14 |
| GO:0046906 | tetrapyrrole binding | MF | 1.73E-15 | 2.82E-14 |
| GO:0005886 | plasma membrane | CC | 2.71E-15 | 4.23E-14 |
| GO:0030286 | dynein complex | CC | 5.32E-14 | 7.98E-13 |
| GO:0005506 | iron ion binding | MF | 1.52E-13 | 2.20E-12 |
| GO:0016705 | oxidoreductase activity, acting on paired donors, with incorporation or reduction of molecular oxygen | MF | 1.45E-12 | 1.89E-11 |
| GO:0016712 | oxidoreductase activity, acting on paired donors, with incorporation or reduction of molecular oxygen, reduced flavin or flavoprotein as one donor, and incorporation of one atom of oxygen | MF | 1.55E-12 | 1.89E-11 |
| GO:0005328 | neurotransmitter:sodium symporter activity | MF | 1.55E-12 | 1.89E-11 |
| GO:0005326 | neurotransmitter transporter activity | MF | 1.55E-12 | 1.89E-11 |
| GO:0015370 | solute:sodium symporter activity | MF | 1.55E-12 | 1.89E-11 |
| GO:0007155 | cell adhesion | BP | 1.75E-12 | 2.01E-11 |
| GO:0022610 | biological adhesion | BP | 1.75E-12 | 2.01E-11 |
| GO:0016887 | ATPase activity | MF | 1.09E-11 | 1.22E-10 |
| GO:0015294 | solute:cation symporter activity | MF | 1.49E-11 | 1.61E-10 |
| GO:0044421 | extracellular region part | CC | 2.02E-11 | 2.13E-10 |
| GO:0004866 | endopeptidase inhibitor activity | MF | 2.25E-11 | 2.25E-10 |
| GO:0061135 | endopeptidase regulator activity | MF | 2.25E-11 | 2.25E-10 |
| GO:0005875 | microtubule associated complex | CC | 2.90E-11 | 2.83E-10 |
| GO:0022804 | active transmembrane transporter activity | MF | 8.38E-11 | 7.97E-10 |
| GO:0044237 | cellular metabolic process | BP | 8.85E-11 | 8.22E-10 |
| GO:0003774 | motor activity | MF | 1.02E-10 | 9.26E-10 |
| GO:0005856 | cytoskeleton | CC | 1.37E-10 | 1.22E-09 |
| GO:0006836 | neurotransmitter transport | BP | 2.96E-10 | 2.57E-09 |
| GO:0015293 | symporter activity | MF | 4.55E-10 | 3.85E-09 |
| GO:0005509 | calcium ion binding | MF | 1.15E-09 | 9.53E-09 |
| GO:0030414 | peptidase inhibitor activity | MF | 1.88E-09 | 1.49E-08 |
| GO:0061134 | peptidase regulator activity | MF | 1.88E-09 | 1.49E-08 |
| GO:0015291 | secondary active transmembrane transporter activity | MF | 2.39E-09 | 1.86E-08 |
| GO:0015630 | microtubule cytoskeleton | CC | 8.48E-09 | 6.48E-08 |
| GO:0046872 | metal ion binding | MF | 8.83E-09 | 6.62E-08 |
| GO:0044765 | single-organism transport | BP | 1.04E-08 | 7.65E-08 |
| GO:0005215 | transporter activity | MF | 1.26E-08 | 9.07E-08 |
| GO:0043169 | cation binding | MF | 1.49E-08 | 1.06E-07 |
| GO:0022892 | substrate-specific transporter activity | MF | 2.00E-08 | 1.39E-07 |
| GO:0022857 | transmembrane transporter activity | MF | 2.20E-08 | 1.51E-07 |
| GO:0004497 | monooxygenase activity | MF | 2.71E-08 | 1.82E-07 |
| GO:0004857 | enzyme inhibitor activity | MF | 2.95E-08 | 1.95E-07 |
| GO:0044238 | primary metabolic process | BP | 8.73E-08 | 5.68E-07 |
| GO:0007165 | signal transduction | BP | 9.70E-08 | 6.20E-07 |
| GO:0044430 | cytoskeletal part | CC | 1.06E-07 | 6.65E-07 |
| GO:0044700 | single organism signaling | BP | 1.31E-07 | 8.09E-07 |
| GO:0071704 | organic substance metabolic process | BP | 1.33E-07 | 8.09E-07 |
| GO:0007018 | microtubule-based movement | BP | 1.36E-07 | 8.14E-07 |
| GO:0044260 | cellular macromolecule metabolic process | BP | 1.40E-07 | 8.28E-07 |
| GO:0023052 | signaling | BP | 1.43E-07 | 8.33E-07 |
| GO:0003777 | microtubule motor activity | MF | 1.73E-07 | 9.90E-07 |
| GO:0007154 | cell communication | BP | 3.30E-07 | 1.87E-06 |
| GO:0008150 | biological_process | BP | 3.80E-07 | 2.12E-06 |
| GO:0003676 | nucleic acid binding | MF | 4.36E-07 | 2.40E-06 |
| GO:0015081 | sodium ion transmembrane transporter activity | MF | 6.14E-07 | 3.33E-06 |
| GO:0006928 | cellular component movement | BP | 6.40E-07 | 3.42E-06 |
| GO:0005578 | proteinaceous extracellular matrix | CC | 7.41E-07 | 3.91E-06 |
| GO:0022890 | inorganic cation transmembrane transporter activity | MF | 1.83E-06 | 9.54E-06 |
| GO:0007017 | microtubule-based process | BP | 2.37E-06 | 1.22E-05 |
| GO:0046873 | metal ion transmembrane transporter activity | MF | 2.71E-06 | 1.37E-05 |
| GO:0055114 | oxidation-reduction process | BP | 2.82E-06 | 1.41E-05 |
| GO:0051716 | cellular response to stimulus | BP | 2.89E-06 | 1.43E-05 |
| GO:0043492 | ATPase activity, coupled to movement of substances | MF | 4.14E-06 | 2.00E-05 |
| GO:0015075 | ion transmembrane transporter activity | MF | 4.16E-06 | 2.00E-05 |
| GO:0006807 | nitrogen compound metabolic process | BP | 4.77E-06 | 2.27E-05 |
| GO:0031012 | extracellular matrix | CC | 4.97E-06 | 2.34E-05 |
| GO:0008324 | cation transmembrane transporter activity | MF | 5.35E-06 | 2.48E-05 |
| GO:0034641 | cellular nitrogen compound metabolic process | BP | 8.01E-06 | 3.67E-05 |
| GO:0005615 | extracellular space | CC | 8.38E-06 | 3.78E-05 |
| GO:1901360 | organic cyclic compound metabolic process | BP | 8.43E-06 | 3.78E-05 |
| GO:0009058 | biosynthetic process | BP | 9.00E-06 | 3.99E-05 |
| GO:0005576 | extracellular region | CC | 9.87E-06 | 4.32E-05 |
| GO:0016787 | hydrolase activity | MF | 1.05E-05 | 4.57E-05 |
| GO:0006725 | cellular aromatic compound metabolic process | BP | 1.09E-05 | 4.67E-05 |
| GO:0046483 | heterocycle metabolic process | BP | 1.11E-05 | 4.70E-05 |
| GO:0022891 | substrate-specific transmembrane transporter activity | MF | 1.14E-05 | 4.77E-05 |
| GO:0003674 | molecular_function | MF | 1.36E-05 | 5.65E-05 |
| GO:1901576 | organic substance biosynthetic process | BP | 1.53E-05 | 6.26E-05 |
| GO:0006139 | nucleobase-containing compound metabolic process | BP | 1.88E-05 | 7.64E-05 |
| GO:0044249 | cellular biosynthetic process | BP | 1.93E-05 | 7.77E-05 |
| GO:0015077 | monovalent inorganic cation transmembrane transporter activity | MF | 3.86E-05 | 0.000153434 |
| GO:0016491 | oxidoreductase activity | MF | 4.51E-05 | 0.000177791 |
| GO:0005319 | lipid transporter activity | MF | 7.01E-05 | 0.00027348 |
| GO:0010467 | gene expression | BP | 7.74E-05 | 0.000298731 |
| GO:0043170 | macromolecule metabolic process | BP | 7.86E-05 | 0.000300578 |
| GO:0005003 | ephrin receptor activity | MF | 7.97E-05 | 0.000301748 |
| GO:0004222 | metalloendopeptidase activity | MF | 9.06E-05 | 0.000339795 |
| GO:0043228 | non-membrane-bounded organelle | CC | 0.000101252 | 0.000359854 |
| GO:0043232 | intracellular non-membrane-bounded organelle | CC | 0.000101252 | 0.000359854 |
| GO:0008272 | sulfate transport | BP | 0.000101497 | 0.000359854 |
| GO:0015116 | sulfate transmembrane transporter activity | MF | 0.000101497 | 0.000359854 |
| GO:1901682 | sulfur compound transmembrane transporter activity | MF | 0.000101497 | 0.000359854 |
| GO:0072348 | sulfur compound transport | BP | 0.000101497 | 0.000359854 |
| GO:0050896 | response to stimulus | BP | 0.000102444 | 0.000359937 |
| GO:0006810 | transport | BP | 0.000116089 | 0.000404237 |
| GO:0051234 | establishment of localization | BP | 0.000120356 | 0.000415388 |
| GO:0043167 | ion binding | MF | 0.000127506 | 0.000436206 |
| GO:0090304 | nucleic acid metabolic process | BP | 0.00013987 | 0.000474343 |
| GO:0030234 | enzyme regulator activity | MF | 0.000143712 | 0.00048317 |
| GO:0042623 | ATPase activity, coupled | MF | 0.000158463 | 0.000528209 |
| GO:0016459 | myosin complex | CC | 0.00016503 | 0.000545438 |
| GO:0051179 | localization | BP | 0.000196901 | 0.000645307 |
| GO:0006811 | ion transport | BP | 0.000206927 | 0.000672513 |
| GO:0009059 | macromolecule biosynthetic process | BP | 0.000310379 | 0.001000397 |
| GO:0034645 | cellular macromolecule biosynthetic process | BP | 0.000331077 | 0.00105836 |
| GO:0008271 | secondary active sulfate transmembrane transporter activity | MF | 0.00038247 | 0.001212708 |
| GO:0006820 | anion transport | BP | 0.000413205 | 0.001299596 |
| GO:0034702 | ion channel complex | CC | 0.000527029 | 0.001631281 |
| GO:0034703 | cation channel complex | CC | 0.000527029 | 0.001631281 |
| GO:0016740 | transferase activity | MF | 0.000561411 | 0.001724018 |
| GO:0016070 | RNA metabolic process | BP | 0.000608197 | 0.00184163 |
| GO:0015103 | inorganic anion transmembrane transporter activity | MF | 0.000609154 | 0.00184163 |
| GO:0008509 | anion transmembrane transporter activity | MF | 0.000704288 | 0.002112863 |
| GO:0005283 | sodium:amino acid symporter activity | MF | 0.000747946 | 0.002176858 |
| GO:0003956 | NAD(P)+-protein-arginine ADP-ribosyltransferase activity | MF | 0.000747946 | 0.002176858 |
| GO:0005343 | organic acid:sodium symporter activity | MF | 0.000747946 | 0.002176858 |
| GO:0005416 | cation:amino acid symporter activity | MF | 0.000747946 | 0.002176858 |
| GO:0043234 | protein complex | CC | 0.000779813 | 0.002252794 |
| GO:0008152 | metabolic process | BP | 0.000822281 | 0.00234212 |
| GO:0044267 | cellular protein metabolic process | BP | 0.000822745 | 0.00234212 |
| GO:0044271 | cellular nitrogen compound biosynthetic process | BP | 0.000912665 | 0.002579271 |
| GO:0006793 | phosphorus metabolic process | BP | 0.000921049 | 0.002584239 |
| GO:0006796 | phosphate-containing compound metabolic process | BP | 0.000981931 | 0.002735379 |
| GO:1901362 | organic cyclic compound biosynthetic process | BP | 0.001005718 | 0.002781774 |
| GO:0018130 | heterocycle biosynthetic process | BP | 0.001144491 | 0.00314332 |
| GO:0006869 | lipid transport | BP | 0.00120687 | 0.003291463 |
| GO:0019438 | aromatic compound biosynthetic process | BP | 0.001281306 | 0.003470204 |
| GO:0010876 | lipid localization | BP | 0.001407224 | 0.003784947 |
| GO:0003810 | protein-glutamine gamma-glutamyltransferase activity | MF | 0.001510951 | 0.004036101 |
| GO:0044446 | intracellular organelle part | CC | 0.001827612 | 0.004848766 |
| GO:0034654 | nucleobase-containing compound biosynthetic process | BP | 0.001854683 | 0.00488734 |
| GO:0044422 | organelle part | CC | 0.001903367 | 0.004981968 |
| GO:0022843 | voltage-gated cation channel activity | MF | 0.001916173 | 0.00498205 |
| GO:0018149 | peptide cross-linking | BP | 0.002115002 | 0.005462588 |
| GO:0008237 | metallopeptidase activity | MF | 0.002149792 | 0.005515913 |
| GO:0055085 | transmembrane transport | BP | 0.002279204 | 0.005809736 |
| GO:0051260 | protein homooligomerization | BP | 0.002459547 | 0.006228723 |
| GO:0016755 | transferase activity, transferring amino-acyl groups | MF | 0.002849601 | 0.007169964 |
| GO:0016772 | transferase activity, transferring phosphorus-containing groups | MF | 0.003071853 | 0.007679633 |
| GO:0005887 | integral to plasma membrane | CC | 0.003448993 | 0.008513336 |
| GO:0031226 | intrinsic to plasma membrane | CC | 0.003448993 | 0.008513336 |
| GO:0004175 | endopeptidase activity | MF | 0.00369455 | 0.009062103 |
| GO:0042626 | ATPase activity, coupled to transmembrane movement of substances | MF | 0.003991449 | 0.009729157 |
| GO:0015399 | primary active transmembrane transporter activity | MF | 0.004461657 | 0.01067513 |
| GO:0015405 | P-P-bond-hydrolysis-driven transmembrane transporter activity | MF | 0.004461657 | 0.01067513 |
| GO:0051259 | protein oligomerization | BP | 0.004461657 | 0.01067513 |
| GO:0015629 | actin cytoskeleton | CC | 0.004493805 | 0.010686487 |
| GO:0005244 | voltage-gated ion channel activity | MF | 0.00466762 | 0.010966096 |
| GO:0022832 | voltage-gated channel activity | MF | 0.00466762 | 0.010966096 |
| GO:0032774 | RNA biosynthetic process | BP | 0.005651892 | 0.013199029 |
| GO:0043231 | intracellular membrane-bounded organelle | CC | 0.005845974 | 0.013490709 |
| GO:0043227 | membrane-bounded organelle | CC | 0.005845974 | 0.013490709 |
| GO:0003677 | DNA binding | MF | 0.005933068 | 0.013611156 |
| GO:0006351 | transcription, DNA-dependent | BP | 0.006020329 | 0.013650747 |
| GO:0019222 | regulation of metabolic process | BP | 0.006020329 | 0.013650747 |
| GO:0005515 | protein binding | MF | 0.006080654 | 0.013689478 |
| GO:0016820 | hydrolase activity, acting on acid anhydrides, catalyzing transmembrane movement of substances | MF | 0.006107613 | 0.013689478 |
| GO:0044459 | plasma membrane part | CC | 0.006580946 | 0.014581573 |
| GO:0050794 | regulation of cellular process | BP | 0.006611091 | 0.014581573 |
| GO:0031323 | regulation of cellular metabolic process | BP | 0.006617791 | 0.014581573 |
| GO:0015698 | inorganic anion transport | BP | 0.00671532 | 0.014713341 |
| GO:0080090 | regulation of primary metabolic process | BP | 0.00738896 | 0.016098852 |
| GO:0008076 | voltage-gated potassium channel complex | CC | 0.007625223 | 0.016430039 |
| GO:0034705 | potassium channel complex | CC | 0.007625223 | 0.016430039 |
| GO:0016301 | kinase activity | MF | 0.007807705 | 0.016730796 |
| GO:0050789 | regulation of biological process | BP | 0.008966864 | 0.019003395 |
| GO:0019219 | regulation of nucleobase-containing compound metabolic process | BP | 0.009063158 | 0.019003395 |
| GO:0051171 | regulation of nitrogen compound metabolic process | BP | 0.009063158 | 0.019003395 |
| GO:0060255 | regulation of macromolecule metabolic process | BP | 0.009063158 | 0.019003395 |
| GO:0005245 | voltage-gated calcium channel activity | MF | 0.010430172 | 0.021308717 |
| GO:0005891 | voltage-gated calcium channel complex | CC | 0.010430172 | 0.021308717 |
| GO:0006471 | protein ADP-ribosylation | BP | 0.010430172 | 0.021308717 |
| GO:0034704 | calcium channel complex | CC | 0.010430172 | 0.021308717 |
| GO:0009889 | regulation of biosynthetic process | BP | 0.010435808 | 0.021308717 |
| GO:0031326 | regulation of cellular biosynthetic process | BP | 0.010600422 | 0.021532107 |
| GO:0010556 | regulation of macromolecule biosynthetic process | BP | 0.010937358 | 0.022101396 |
| GO:2000112 | regulation of cellular macromolecule biosynthetic process | BP | 0.011109758 | 0.02233405 |
| GO:0010468 | regulation of gene expression | BP | 0.011284834 | 0.022569667 |
| GO:0051252 | regulation of RNA metabolic process | BP | 0.012393786 | 0.024661106 |
| GO:2001141 | regulation of RNA biosynthetic process | BP | 0.012786757 | 0.025313885 |
| GO:0006355 | regulation of transcription, DNA-dependent | BP | 0.012987817 | 0.025582063 |
| GO:0046914 | transition metal ion binding | MF | 0.01340361 | 0.026268381 |
| GO:0016773 | phosphotransferase activity, alcohol group as acceptor | MF | 0.013759205 | 0.02683045 |
| GO:0004714 | transmembrane receptor protein tyrosine kinase activity | MF | 0.014237356 | 0.02762472 |
| GO:0004869 | cysteine-type endopeptidase inhibitor activity | MF | 0.01433993 | 0.027686003 |
| GO:0032991 | macromolecular complex | CC | 0.014595407 | 0.028040436 |
| GO:0007169 | transmembrane receptor protein tyrosine kinase signaling pathway | BP | 0.016416936 | 0.031385319 |
| GO:0005249 | voltage-gated potassium channel activity | MF | 0.018206701 | 0.034637138 |
| GO:0065007 | biological regulation | BP | 0.018306029 | 0.034657045 |
| GO:0043412 | macromolecule modification | BP | 0.019157358 | 0.036093572 |
| GO:0044281 | small molecule metabolic process | BP | 0.020027025 | 0.037550673 |
| GO:0016310 | phosphorylation | BP | 0.023359693 | 0.043589858 |
| GO:0046373 | L-arabinose metabolic process | BP | 0.027401747 | 0.048818113 |
| GO:0046556 | alpha-N-arabinofuranosidase activity | MF | 0.027401747 | 0.048818113 |
| GO:0006069 | ethanol oxidation | BP | 0.027401747 | 0.048818113 |
| GO:0051903 | S-(hydroxymethyl)glutathione dehydrogenase activity | MF | 0.027401747 | 0.048818113 |
| GO:0042043 | neurexin family protein binding | MF | 0.027401747 | 0.048818113 |
| GO:0007259 | JAK-STAT cascade | BP | 0.027401747 | 0.048818113 |
| GO:0019566 | arabinose metabolic process | BP | 0.027401747 | 0.048818113 |
| GO:0006067 | ethanol metabolic process | BP | 0.027401747 | 0.048818113 |
| GO:0034308 | primary alcohol metabolic process | BP | 0.027401747 | 0.048818113 |
| GO:0006464 | cellular protein modification process | BP | 0.027538423 | 0.048818113 |
| GO:0036211 | protein modification process | BP | 0.027538423 | 0.048818113 |

Table S14: Kyoto Encyclopedia of Genes and Genomes (KEGG) enrichment analysis of contracted gene families.

| Map number | Pathway | Count | *P*-value | *Q*-value |
| --- | --- | --- | --- | --- |
| map04750 | Inflammatory mediator regulation of TRP channels | 10 | 2.28E-05 | 0.000393967 |
| map04145 | Phagosome | 11 | 3.52E-05 | 0.000531999 |
| map04610 | Complement and coagulation cascades | 10 | 7.54E-05 | 0.001008416 |
| map05322 | Systemic lupus erythematosus | 8 | 8.33E-05 | 0.001008416 |
| map02010 | ABC transporters | 5 | 0.000206431 | 0.002270744 |
| map04962 | Vasopressin-regulated water reabsorption | 4 | 0.001128789 | 0.011381958 |
| map05140 | Leishmaniasis | 5 | 0.001496758 | 0.013931359 |
| map05150 | Staphylococcus aureus infection | 5 | 0.003892555 | 0.033642794 |
| map00980 | Metabolism of xenobiotics by cytochrome P450 | 3 | 0.005521821 | 0.044542686 |

Table S15: Two cluster analysis of mustache toad and other species. The evolutionary rates were calculated by LINTRE through the super-genes (protein) which produced by orthoMCL. The branch length was calculated using zebrafish as the outgroup. Delta = | bA - bB |; Z = delta/s.e. (delta/standard error); CP (confident probability) = 1 - pvalue.

| Outgroup | Ingroup1 | Ingroup2 | bA | bB | delta | s.e. | Z | CP |
| --- | --- | --- | --- | --- | --- | --- | --- | --- |
| *D. rerio* | *G. gallus* | *V. ailaonica* | 0.203008 | 0.314883 | 0.111874 | 0.003857 | 29.008163 | 99.96% |
| *D. rerio* | *A. sinensis* | *V. ailaonica* | 0.199044 | 0.306048 | 0.107004 | 0.003847 | 27.817449 | 99.96% |
| *D. rerio* | *A. carolinensis* | *V. ailaonica* | 0.237145 | 0.324800 | 0.087654 | 0.004064 | 21.570278 | 99.96% |
| *D. rerio* | *X. tropicalis* | *V. ailaonica* | 0.129560 | 0.234508 | 0.104948 | 0.003386 | 30.994675 | 99.96% |
| *D. rerio* | *H. sapiens* | *V. ailaonica* | 0.248182 | 0.330928 | 0.082747 | 0.004046 | 20.451058 | 99.96% |
| *D. rerio* | *N. parkeri* | *V. ailaonica* | 0.133430 | 0.224846 | 0.091416 | 0.003358 | 27.219744 | 99.96% |
| *D. rerio* | *R. marina* | *V. ailaonica* | 0.158464 | 0.234383 | 0.075919 | 0.003805 | 19.951580 | 99.96% |
| *D. rerio* | *R. catesbeiana* | *V. ailaonica* | 0.174937 | 0.193342 | 0.018405 | 0.004422 | 4.161979 | 99.96% |
| *D. rerio* | *A. mexicanum* | *V. ailaonica* | 1.714469 | 0.288089 | 1.426381 | 0.016294 | 87.540282 | 99.96% |

Table S16: The relative evolutionary rate of mustache toad and other species analyzed by Tajima’s Test.

| Outgroup | Ingroup A | Ingroup B | Identical | Ingroup A specific | Ingroup B specific | Chi-score | P-value |
| --- | --- | --- | --- | --- | --- | --- | --- |
| *D. rerio* | *G. gallus* | *V. ailaonica* | 49087 | 6103 | 9809 | 863.15 | P<0.00001 |
| *D. rerio* | *A. sinensis* | *V. ailaonica* | 48266 | 5901 | 9381 | 792.46 | P<0.00001 |
| *D. rerio* | *A. carolinensis* | *V. ailaonica* | 47213 | 6709 | 9473 | 472.11 | P<0.00001 |
| *D. rerio* | *X. tropicalis* | *V. ailaonica* | 51383 | 4148 | 7550 | 989.37 | P<0.00001 |
| *D. rerio* | *H. sapiens* | *V. ailaonica* | 49017 | 7303 | 10011 | 423.55 | P<0.00001 |
| *D. rerio* | *N. parkeri* | *V. ailaonica* | 51068 | 4456 | 7461 | 757.74 | P<0.00001 |
| *D. rerio* | *R. marina* | *V. ailaonica* | 45118 | 4618 | 6761 | 403.59 | P<0.00001 |
| *D. rerio* | *R. catesbeiana* | *V. ailaonica* | 29941 | 3477 | 3833 | 17.34 | P = 0.00003 |
| *D. rerio* | *A. mexicanum* | *V. ailaonica* | 23500 | 27357 | 5826 | 13970.53 | P<0.00001 |


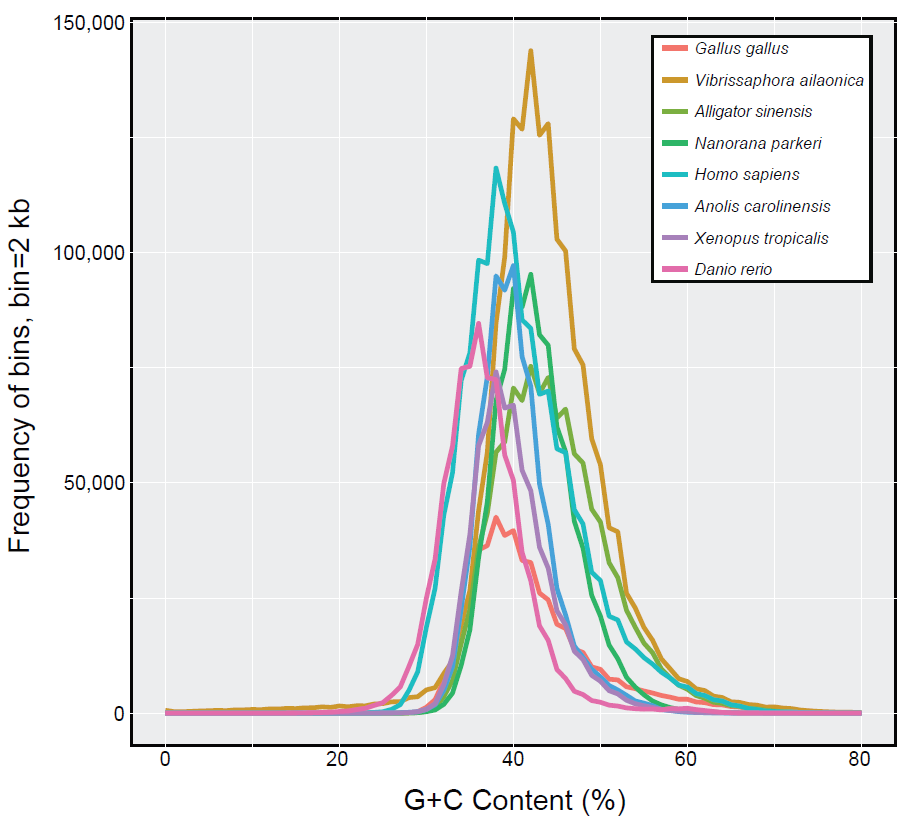


Figure S1: The GC content in these genomes. The species including *Gallus gallus*, *Vibrissaphora ailaonica*, *Alligator sinensis*, *Nanorana parkeri*, *Homo sapiens*, *Anolis carolinensis*, *Xenopus tropicalis*, and *Danio rerio*.


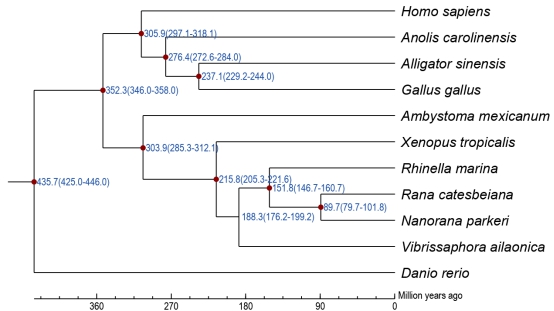
Figure S2: The divergence time of these species (using first-codon sites). The species including *Danio rerio*, *Rana catesbeiana*, *Rhinella marina*, *Vibrissaphora ailaonica*, *Nanorana parkeri*, *Homo sapiens*, *Gallus gallus*, *Anolis carolinensis*, *Xenopus tropicalis*, *Ambystoma mexicanum*, and *Alligator sinensis*.


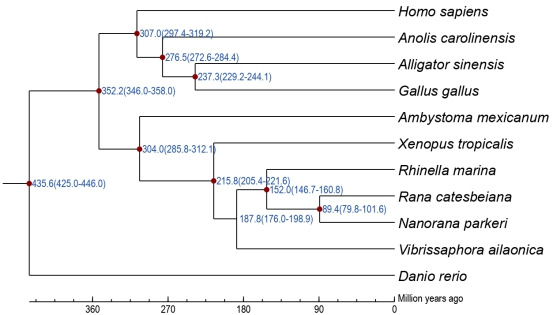


Figure S3: The divergence time of these species (using second-codon sites). The species including *Danio rerio*, *Rana catesbeiana*, *Rhinella marina*, *Vibrissaphora ailaonica*, *Nanorana parkeri*, *Homo sapiens*, *Gallus gallus*, *Anolis carolinensis*, *Xenopus tropicalis*, *Ambystoma mexicanum*, and *Alligator sinensis*.
