# Supplementary material for: Chromosome-level assembly of the mustache toad genome using third-generation DNA sequencing and Hi-C analysis
Source: Gigascience. 2019 Sep 23;8(9):giz114. doi: 10.1093/gigascience/giz114 (PMC6755253; doi:10.1093/gigascience/giz114)
Supplement: giz114_Response_to_Reviewer_Comments_Original_Submission [file giz114_response_to_reviewer_comments_original_submission.pdf]

Author's Response To Reviewer Comments

Close

Responses to Reviewers

The authors would like to thank the reviewers for the helpful comments, and we have revised our manuscript accordingly. At this time, we hope to have an opportunity to publish this paper in GigaScience.

Reviewer 1 Comments for the Author...

Reviewer #1: Li et al report the first genome assembly of a mustache toad. They used a combination of PacBio and HiC to generate a highly-contiguous assembly.
They used RNA-seq data, ab intio gene prediction and homology to annotate ~26000 genes, analyzed gene family contractions and expansions, and estimated the phylogenetic relationship to other amphibians. Given the sparsity of amphibian genomes, this assembly will be valuable for the community. I recommend accepting the manuscript after a few issues have been addressed, most of which are minor.

Major comments:

1) Since k-mer based genome size estimation is often not very precise, I find the redundancy reduction of the assembly potentially problematic. The authors removed contigs that overlap with at least 70% another contig using an alignment identity cutoff of 70%. It feels a bit like these parameters were optimized such that the final assembly matches the k-mer predicted size. E.g. the 70% identity cutoff is not compatible with the error rate of PacBio reads, unless the purpose of the Redundans run was to remove single reads that contain much more than the ~15% expected error rate. Also, heterozygosity and alt haplotypes should not result in 30% divergence.
I wonder if the authors can check which contigs were removed in this step and ensure that no real sequences were removed. If there is any doubt that some of the contigs may contain functionally important sequences (genes, etc), then I would suggest to provide the removed contigs with the redundancy-filtered assembly as an extra fasta file. Specifically, I wonder if the slightly lower BUSCO scores can be explained by removing real contigs based on 70% similarity.

Response: To make sure all the removed contigs not contain real sequences, we checked the BUSCO of the raw genome and the redundancy-filtered genome with eukaryote and metazoan as datasets at the same time. Besides, we also checked the mapping ratio of Illumina reads on the raw genome and the redundancy-filtered genome. The BUSCO results shown that most of the genes were not removed and the mapping ratio results shown that both coding region and non-coding region were remained (Table S5). All these results indicated that the redundancy-filtered step not removed many real sequences. However, as you have said before, we further checked the contigs that were removed in this step, all the removed contigs, the BUSCO results of genome and the redundancy-filtered assembly have been uploaded to GigaDB FTP server. Thank you for your suggestions.

2) The manuscript 'undersells' the contiguity of mustache toad assembly, which has *substantially higher* contig and scaffold N50 values than any other amphibian genome.
I therefore recommend to place Table S8 in the main text.

Response: Done. Table S8 has been removed to main text (Table 4). Thank you.

3) Table 3 is hard to understand as absolute numbers are reported. A much better way would be to report '%complete genes, %complete and duplicated genes, %fragmented genes, %missing genes' which sums to 100%. In addition, these 4 BUSCO percentages for the other amphibian genomes should be added to this table to provide a direct comparison of genome assembly completeness.

Response: Table 3 has been corrected and the BUSCO results of the other amphibian genomes were also

added.

4) I wonder how the divergence time estimates would change if first or second codon positions instead of four-fold degenerate sites were used. This may be relevant as four-fold degenerate sites are clearly saturated over these phylogenetic distances. Also, the divergence times shown in Figure 6 are quite different to the times from timetree, where e.g. the Rana - Nanorana split was 89 Mya (Figure 6, 44 Mya) and the Rana - Rhinella split was 160 Mya (Figure 6, 137 Mya).

Response: We added the fossil evidence between Rana - Nanorana and Rana - Rhinella from timetree to run mcmctree tree again, and both four-fold degenerate sites and first or second codon positions were used for the divergence time analysis (Figure 6; Figures S2 and S3). The results shown that these three results are much closed and the divergence time between Rana - Nanorana, Rana - Rhinella are similar to timetree results. We have revised the divergence time results in Figure 6. Thank you.
Besides, because the expansion and contraction analysis of gene family are related the divergence time result, so we updated the expansion and contraction results this time, including all the descriptions and Tables (Tables S11-S14). Thank you.

Minor comments:
1) The manuscript should be edited by a native speaker to improve the language.
A few examples: "Like other mustache toad species, V. ailaonica males develop temporary keratinized nuptial spines on their upper jaw during each breeding season and fall off when the breeding season ends, which probably lead to the reverse of the sexual size dimorphism, namely the size of the male get larger than female." should be improved to "Like other mustache toad species, V. ailaonica males temporarily develop keratinized nuptial spines on their upper jaw during each breeding season that fall off when the breeding season ends, which probably reversed the sexual size dimorphism with males being larger than females."
"To investigate the genetic mechanism of the repeatedly develop the keratinized spines"
-->
"To investigate the genetic mechanism of the repeatedly developed keratinized spines"

"Another unique aspect of the mustache toad is that breeding occurs during the cold season, unlike most frogs and toads which breed in the warmer months"
-->
"Another unique aspect of the mustache toad is that breeding occurs during the cold season, whereas most frogs and toads breed in the warmer months"
etc.

Response: Done. We have corrected the above mistakes and other mistakes in our manuscript. Thank you.

2) Please reference Figure 1 in line 55, where the temporary spines are described.

Response: Corrected.

3) Line 75-76: I find this outlook that we will learn from the toad genome (sex dimorphism) how body size control works in general a bit far-stretched. This could be removed.

Response: Corrected.

4) Line 94/95: Please mention the Illumina read length (paired end 150 bp reads). I find this information more important than library size.

Response: Corrected.

5) Line 164/165: The conclusion that the toad assembly is very complete is justified based on the high percentage of mapping RNA-seq reads and transcripts. However, this sentence should be moved to Line 161 (after "Table S8).", where this analysis is done.

Response: Corrected.

6) Line 180: Please replace 'closely-related' with 'vertebrate' as zebrafish, lamprey and amphibians are

not really closely related.

Response: Corrected.

7) Line 179: Would an Augustus model trained from an amphibian (e.g. xenopus) be not more appropriate than a zebrafish model?

Response: The official website of Augustus not included the amphibian species as model. So we selected zebrafish that has high-quality gene set as model. Thank you for your suggestions.

8) Table 4: Please round the percentage to 2 digits (9.94%).

Response: Corrected.

9) Line 204/205: The references don't match: Reference 34 (www.axolotl-omics.org) and 36 refer to the Ambystoma genome assembly. The Rhinella reference is missing.

Response: Corrected.

Reviewer 2 Comments for the Author...

Reviewer #2: Here, Yongxin Li and colleagues reported the chromosome-level genome with the full annotation of the mustache toad, Vibrissaphora ailaonica, using conventional paired-end short read, sufficient amount of PacBio long reads and chromosome conformation capture (Hi-C) data. Although there are several amphibian genomes reported previously, many of them do not have chromosome-level genomes, so I think this is definitely a valuable resource to the community, especially to study the synteny of amphibian genome. So I would like to recommend accepting this manuscript for publication after resolving some issues as mentioned below:

1) On page 5, more details for RNA-Seq library prep construction method should be provided (poly-A capturing or ribosome-depletion? Which library kit do they use?). Also, even the authors mentioned that 9 tissues were dissected from the biospecimen they sequenced the genome (page 4, line 85), it is not clear whether all those tissues were used in this 'mixed RNA-Seq' experiment. Please provide more details for this experiment.

Response: The datails for RNA-seq library construction method were added. About the RNA-seq experiment, after equally mixed the DNA of the 9 tissues, the mixed DNA sample was used for library construction and RNA-seq experiments. Thank you.

2) On page 5, the authors mentioned that they used four Hi-C libraries. Are they constructed from the same samples (blood), with the same parameter (four technical replicates)? Or using different samples? If they used a different parameter to construct these four libraries, it should be specified.

Response: Yes, all these four Hi-C libraries were used the same samples with the same parameter. We also clarify these informations in the main text this time. Thank you for your suggestions.

3) Authors claimed that they deposited the data on PRJNA523649, but it looks like they uploaded one single file for each data set. Because they used different libraries, at least for paired-end seq (Table S1) and HiC-seq (Table S4), it would be better to provide those raw data separately.

Response: Thank you for your suggestions, we uploaded these sequencing data in the same PROJECT ID, but with different SRA IDs, you could see this by this link (https://www.ncbi.nlm.nih.gov/sra/?term=PRJNA523649). Thank you.

Close
