# Supplementary material for: Chromosome-level assembly of the mustache toad genome using third-generation DNA sequencing and Hi-C analysis
Source: Gigascience. 2019 Sep 23;8(9):giz114. doi: 10.1093/gigascience/giz114 (PMC6755253; doi:10.1093/gigascience/giz114)
Supplement: giz114_Response_to_Reviewer_Comments_Revision_1 [file giz114_response_to_reviewer_comments_revision_1.pdf]

Author's Response To Reviewer Comments

Close

All the comments in this version have been corrected.

Close
