# Supplementary material for: Chromosome-level assembly of the mustache toad genome using third-generation DNA sequencing and Hi-C analysis
Source: Gigascience. 2019 Sep 23;8(9):giz114. doi: 10.1093/gigascience/giz114 (PMC6755253; doi:10.1093/gigascience/giz114)
Supplement: giz114_Reviewer_1_Report_Original_Submission — Michael Hiller -- 5/4/2019 Reviewed [file giz114_reviewer_1_report_original_submission.pdf]

**Reviewer Report**

**Title: Chromosome-level assembly of the mustache toad genome using third-generation DNA sequencing and Hi-C analysis**

**Version: Original Submission** **Date:** 5/4/2019

**Reviewer name: Michael Hiller**

**Reviewer Comments to Author:**

Li et al report the first genome assembly of a mustache toad. They used a combination of PacBio and HiC to generate a highly-contiguous assembly.
They used RNA-seq data, ab intio gene prediction and homology to annotate ~26000 genes, analyzed gene family contractions and expansions, and estimated the phylogenetic relationship to other amphibians. Given the sparsity of amphibian genomes, this assembly will be valuable for the community. I recommend accepting the manuscript after a few issues have been addressed, most of which are minor.
Major comments:
1)
Since k-mer based genome size estimation is often not very precise, I find the redundancy reduction of the assembly potentially problematic.
The authors removed contigs that overlap with at least 70% another contig using an alignment identity cutoff of 70%.
It feels a bit like these parameters were optimized such that the final assembly matches the k-mer predicted size.
E.g. the 70% identity cutoff is not compatible with the error rate of PacBio reads, unless the purpose of the Redundans run was to remove single reads that contain much more than the ~15% expected error rate. Also, heterozygosity and alt haplotypes should not result in 30% divergence.
I wonder if the authors can check which contigs were removed in this step and ensure that no real sequences were removed.
If there is any doubt that some of the contigs may contain functionally important sequences (genes, etc), then I would suggest to provide the removed contigs with the redundancy-filtered assembly as an extra fasta file.
Specifically, I wonder if the slightly lower BUSCO scores can be explained by removing real contigs based on 70% similarity.
2)
The manuscript 'undersells' the contiguity of mustache toad assembly, which has *substantially higher* contig and scaffold N50 values than any other amphibian genome.
I therefore recommend to place Table S8 in the main text.
3)
Table 3 is hard to understand as absolute numbers are reported. A much better way would be to report '%complete genes, %complete and duplicated genes, %fragmented genes, %missing genes' which sums

to 100%.
In addition, these 4 BUSCO percentages for the other amphibian genomes should be added to this table to provide a direct comparison of genome assembly completeness.
4)
I wonder how the divergence time estimates would change if first or second codon positions instead of four-fold degenerate sites were used.
This may be relevant as four-fold degenerate sites are clearly saturated over these phylogenetic distances.
Also, the divergence times shown in Figure 6 are quite different to the times from timetree, where e.g. the Rana - Nanorana split was 89 Mya (Figure 6, 44 Mya) and the Rana - Rhinella split was 160 Mya (Figure 6, 137 Mya).
Minor comments:
1)
The manuscript should be edited by a native speaker to improve the language.
A few examples:
"Like other mustache toad species, V. ailaonica males
develop temporary keratinized nuptial spines on their upper jaw during each breeding
season and fall off when the breeding season ends, which probably lead to the reverse
of the sexual size dimorphism, namely the size of the male get larger than female."
should be improved to
"Like other mustache toad species, V. ailaonica males
temporarily develop keratinized nuptial spines on their upper jaw during each breeding
season that fall off when the breeding season ends, which probably reversed the sexual size dimorphism with males being larger than females."
"To investigate the genetic mechanism of the repeatedly develop the keratinized spines"
-->
"To investigate the genetic mechanism of the repeatedly developed keratinized spines"
"Another unique aspect of the mustache toad is that breeding occurs during the cold season, unlike most frogs and toads which breed in the warmer months"
-->
"Another unique aspect of the mustache toad is that breeding occurs during the cold season, whereas most frogs and toads breed in the warmer months"
etc.
2)
Please reference Figure 1 in line 55, where the temporary spines are described.
3)
Line 75-76: I find this outlook that we will learn from the toad genome (sex dimorphism) how body size control works in general a bit far-stretched. This could be removed.
4)
Line 94/95: Please mention the Illumina read length (paired end 150 bp reads). I find this information more important than library size.
5)

Line 164/165: The conclusion that the toad assembly is very complete is justified based on the high percentage of mapping RNA-seq reads and transcripts. However, this sentence should be moved to Line 161 (after "Table S8).", where this analysis is done.
6)
Line 180: Please replace 'closely-related' with 'vertebrate' as zebrafish, lamprey and amphibians are not really closely related.
7) Line 179: Would an Augustus model trained from an amphibian (e.g. xenopus) be not more appropriate than a zebrafish model?
8) Table 4: Please round the percentage to 2 digits (9.94%).
9) Line 204/205: The references don't match: Reference 34 (www.axolotl-omics.org) and 36 refer to the Ambystoma genome assembly.
The Rhinella reference is missing.

**Level of Interest**

Please indicate how interesting you found the manuscript: Choose an item.

**Quality of Written English**

Please indicate the quality of language in the manuscript: Choose an item.

**Declaration of Competing Interests**

Please complete a declaration of competing interests, considering the following questions:

- Have you in the past five years received reimbursements, fees, funding, or salary from an organisation that may in any way gain or lose financially from the publication of this manuscript, either now or in the future?
- Do you hold any stocks or shares in an organisation that may in any way gain or lose financially from the publication of this manuscript, either now or in the future?
- Do you hold or are you currently applying for any patents relating to the content of the manuscript?
- Have you received reimbursements, fees, funding, or salary from an organization that holds or has applied for patents relating to the content of the manuscript?
- Do you have any other financial competing interests?
- Do you have any non-financial competing interests in relation to this paper?

If you can answer no to all of the above, write 'I declare that I have no competing interests' below. If your reply is yes to any, please give details below.

I declare that I have no competing interests

I agree to the open peer review policy of the journal. I understand that my name will be included on my report to the authors and, if the manuscript is accepted for publication, my named report including any attachments I upload will be posted on the website along with the authors' responses. I agree for my report to be made available under an Open Access Creative Commons CC-BY license (http://creativecommons.org/licenses/by/4.0/). I understand that any comments which I do not wish to be included in my named report can be included as confidential comments to the editors, which will not be published.

Choose an item.

To further support our reviewers, we have joined with Publons, where you can gain additional credit to further highlight your hard work (see: https://publons.com/journal/530/gigascience). On publication of this paper, your review will be automatically added to Publons, you can then choose whether or not to claim your Publons credit. I understand this statement.

Yes Choose an item.
