# Supplementary material for: Chromosome-level assembly of the mustache toad genome using third-generation DNA sequencing and Hi-C analysis
Source: Gigascience. 2019 Sep 23;8(9):giz114. doi: 10.1093/gigascience/giz114 (PMC6755253; doi:10.1093/gigascience/giz114)
Supplement: giz114_Reviewer_2_Report_Original_Submission — Taejoon Kwon -- 5/6/2019 Reviewed [file giz114_reviewer_2_report_original_submission.pdf]

Reviewer Report

Title: Chromosome-level assembly of the mustache toad genome using third-generation DNA sequencing and Hi-C analysis

Version: Original Submission **Date:** 5/6/2019

Reviewer name: Taejoon Kwon

Reviewer Comments to Author:

Here, Yongxin Li and colleagues reported the chromosome-level genome with the full annotation of the mustache toad, Vibrissaphora ailaonica, using conventional paired-end short read, sufficient amount of PacBio long reads and chromosome conformation capture (Hi-C) data. Although there are several amphibian genomes reported previously, many of them do not have chromosome-level genomes, so I think this is definitely a valuable resource to the community, especially to study the synteny of amphibian genome. So I would like to recommend accepting this manuscript for publication after resolving some issues as mentioned below:
- On page 5, more details for RNA-Seq library prep construction method should be provided (poly-A capturing or ribosome-depletion? Which library kit do they use?). Also, even the authors mentioned that 9 tissues were dissected from the biospecimen they sequenced the genome (page 4, line 85), it is not clear whether all those tissues were used in this 'mixed RNA-Seq' experiment. Please provide more details for this experiment.
- On page 5, the authors mentioned that they used four Hi-C libraries. Are they constructed from the same samples (blood), with the same parameter (four technical replicates)? Or using different samples? If they used a different parameter to construct these four libraries, it should be specified.
- Authors claimed that they deposited the data on PRJNA523649, but it looks like they uploaded one single file for each data set. Because they used different libraries, at least for paired-end seq (Table S1) and HiC-seq (Table S4), it would be better to provide those raw data separately.

Level of Interest

Please indicate how interesting you found the manuscript: Choose an item.

Quality of Written English

Please indicate the quality of language in the manuscript: Choose an item.

Declaration of Competing Interests

Please complete a declaration of competing interests, considering the following questions:

- Have you in the past five years received reimbursements, fees, funding, or salary from an organisation that may in any way gain or lose financially from the publication of this manuscript, either now or in the future?
- Do you hold any stocks or shares in an organisation that may in any way gain or lose financially from the publication of this manuscript, either now or in the future?
- Do you hold or are you currently applying for any patents relating to the content of the manuscript?
- Have you received reimbursements, fees, funding, or salary from an organization that holds or has applied for patents relating to the content of the manuscript?
- Do you have any other financial competing interests?
- Do you have any non-financial competing interests in relation to this paper?

If you can answer no to all of the above, write 'I declare that I have no competing interests' below. If your reply is yes to any, please give details below.

I declare that I have no competing interests.

I agree to the open peer review policy of the journal. I understand that my name will be included on my report to the authors and, if the manuscript is accepted for publication, my named report including any attachments I upload will be posted on the website along with the authors' responses. I agree for my report to be made available under an Open Access Creative Commons CC-BY license (http://creativecommons.org/licenses/by/4.0/). I understand that any comments which I do not wish to be included in my named report can be included as confidential comments to the editors, which will not be published.

Choose an item.

To further support our reviewers, we have joined with Publons, where you can gain additional credit to further highlight your hard work (see: https://publons.com/journal/530/gigascience). On publication of this paper, your review will be automatically added to Publons, you can then choose whether or not to claim your Publons credit. I understand this statement.

Yes Choose an item.
